# Anthelmintic A-Type Procyanidins and Further Characterization of the Phenolic Composition of a Root Extract from *Paullinia pinnata*

**DOI:** 10.3390/molecules25102287

**Published:** 2020-05-13

**Authors:** Verena Spiegler

**Affiliations:** Institute for Pharmaceutical Biology and Phytochemistry, University of Münster, Corrensstraße 48, 48149 Münster, Germany; verena.spiegler@uni-muenster.de

**Keywords:** anthelmintic, *Paullinia pinnata*, coumarin, cleomiscosin, procyanidin, phenolic, furanone, phenylhexanoid, *Caenorhabditis elegans*

## Abstract

Extracts from the roots of *Paullinia pinnata* L. are used in West Africa as traditional remedies for a variety of diseases including infestations with soil-transmitted helminths. Based on the results of an ethnopharmacological survey in Ghana, an aqueous acetone (70%) extract was investigated for its anthelmintic and phytochemical properties. Partitioning of the crude extract followed by several fractionation steps of the ethyl acetate phase using Sephadex^®^ LH-20, fast centrifugal partition chromatography, RP-18-MPLC and HPLC led to isolation of six oligomeric A-type procyanidins (**1** to **6**). To determine the anthelmintic activity, the crude extract, fractions and isolated compounds were tested in vitro against the model organism *Caenorhabditis elegans*. A significantly better activity was observed for the trimeric A-type procyanidin (**1**) compared to a B-type trimer. However, this effect could not be generalized for the tetrameric procyanidins, for which the type of the interflavan-linkage (4→6 vs. 4→8) had the greatest impact on the bioactivity. Besides the procyanidins, three novel compounds, isofraxidin-7-*O-*α-l-rhamnopyranosyl-(1″→6′)-β-d-glucopyranoside (**17**), 4-methoxycatechol-2-*O*-(5′′-*O*-vanilloyl-β-apiofuranosyl)-(1′′→2′)-β-glucopyranoside (**18**) and a 6-(3-methoxy-4-hydroxyphenyl)-hexane-2,4-diol-2-*O*-hexoside (**19**) were isolated together with further ten known compounds (**7** to **16**), mainly coumarins and coumarinolignans. Except for 3-β-d-glucopyranosyloxy-4-methyl-2(5*H*)-furanone (**15**), none of the isolated compounds has previously been described for *P. pinnata*. The anthelmintic activity was attributed to the presence of procyanidins, but not to any of the other compound classes. In summary, the findings rationalize the traditional use of *P. pinnata* root extracts as anthelmintic remedies.

## 1. Introduction

*Paullinia pinnata* L. (Sapindaceae) is a woody climber growing in tropical regions worldwide. In West Africa and Tanzania, preparations from different parts of the plant have traditionally been used as remedies for the treatment of various diseases, such as dysenterie, nausea, bacterial and parasitic infections, wounds, infertility, cancer and neurological disorders [1,2,3,4,5]. In addition, extracts of leaves and roots have been described for the treatment of helminth infestations, particularly ancylostomiasis [4,6]. Due to the large variety of traditional indications, the number of in vitro investigations is accordingly high: anticancer, antimicrobial [7], anthelmintic [5,8], antiprotozoal [5], molluscicidal [9], vasorelaxant [10], analgesic and anti-inflammatory [11,12] activities have been described.

Regarding their phytochemical composition, extracts from leaves and roots of *P. pinnata* have been quite extensively investigated, however, the polarity of these extracts was mainly in the semi-polar to non-polar range. Thus, several lupane-type triterpenoids [13,14,15] with fibroblast stimulatory [13] and moderate antibacterial activities [15] have been isolated from the roots. Further, leaves and roots were found to contain the phytosterols friedelin, β-sitosterol and β-sitosterolglucoside [16,17] as well as a variety of fatty acids [16]. In addition, a cardiotonic tannin [18], ceramides and cerebrosides [19], several triterpenoids, l-quebrachitol, a trioxaphenanthrenone [17] and two unusual flavone glycosides [20] have been isolated from the leaves. Fewer investigations focused on secondary metabolites from the stem, however, it has been reported to contain oleanane-type triterpenoid saponins with antibacterial and antifungal properties [21,22].

As on the other hand, the main applications in traditional medicine are decoctions or tinctures [3,6], the composition of more polar extracts are of particular interest. Up to now, the presence of condensed tannins in *P. pinnata* has been described several times [3,8,10] for semi-polar to polar extracts. These condensed tannins were found to be mainly A-type procyanidins [8], i.e., condensed tannins that are characterized by an additonal ether linkage between C-2 and a hydroxyl group of the A-ring in the next flavanol unit [23], however, their extact structures have not yet been elucidated. Regarding the bioactivity, a hydroethanolic root extract enriched in these A-type procyanidins exerted anthelmintic activity in vitro against the model organism *Caenorhabditis elegans* as well as the parasitic nematodes *Ancylostoma caninum*, *Toxocara cati* and *Trichuris vulpis* [8].

The aim of the current study was therefore to phytochemically characterize a proanthocyanidin-rich acetone-water (7:3) extract from the roots of *P. pinnata* with respect to tannin composition and presence of other (semi-)polar constituents. In addition, the contribution of A-type procyanidins to the anthelmintic activity of the crude extract was to be further clarified.

## 2. Results

### 2.1. Phytochemical Characterization of the Aqueous Acetone Root Extract

#### 2.1.1. Isolation and Identification of Oligomeric Proanthocyanidins

In order to further investigate the phytochemical composition of semi-polar extracts from *P. pinnata* particularly with respect to condensed tannins, an acetone-water (7:3) extract was prepared from the powdered and defatted roots. The crude extract was partitioned between ethyl acetate and water, separating lower oligomeric proanthocyanidins (PACs) from higher oligomeric and polymeric derivatives. The yield of the aqueous phase which was mainly composed of polymeric PACs as well as mono- and disaccharides, exceeded that of the ethyl acetate (EtOAc) phase by far (161 g vs. 9.3 g).

**Figure 1:**
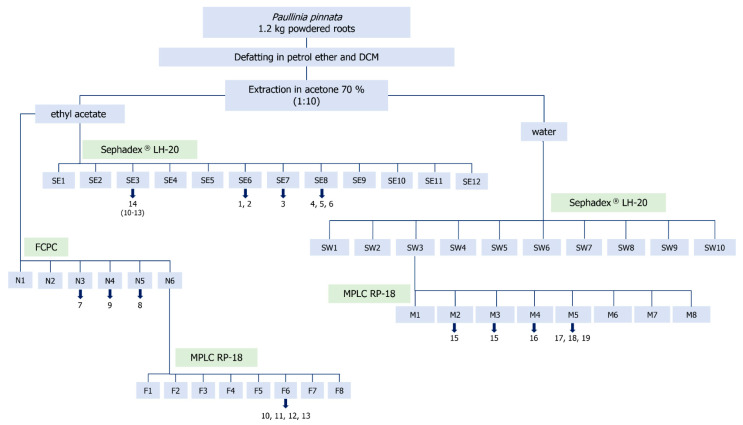
Extraction and fractionation scheme of roots and root extract from *Paullinia pinnata*.

Fractionation of the EtOAc partition on Sephadex^®^ LH20 afforded 12 fractions, most of which exclusively consisted of oligomeric PACs (SE4 to SE12). Figure 1 shows the complete fractionation scheme.

**Figure 2:**
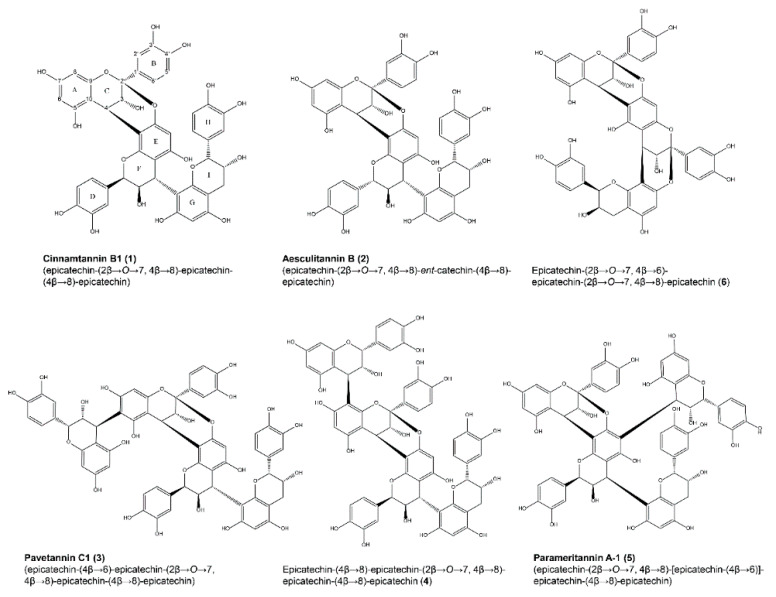
Structures of oligomeric procyanidins isolated from the acetone-water (7:3) extract of the roots of *P. pinnata*. The numeration of carbons and designation of rings in PACs is exemplified for compound **1**.

The major oligomeric PACs shown in Figure 2, also representing the major compounds within the EtOAc partition, were subsequently purified by preparative HPLC and identified as the known compounds cinnamtannin B1 (**1**) [24,25], aesculitannin B (**2**) [25], pavetannin C1 (**3**) [26], epicatechin-(4β→8)-epicatechin-(2β→O→7, 4β→8)-epicatechin-(4β→8)-epicatechin (**4**) [26] and parameritannin A-1 (**5**) [27].

In addition, small amounts of a trimeric A-type PAC (**6**) were isolated, showing an *m/z* value of 861.2 ([M − H]^−^), unlike *m/z* 863.2 as measured for compounds **1** and **2**. Characteristic fragments of *m/z* 285.0 were observed, resulting from a quinone methide cleavage of the doubly linked top unit [28,29] (Figure S1). Sequential cleavage of the next flavan-3-ol unit connected by a single interflavan linkage to the bottom unit yields fragments of *m/z* 287.1 which were detected for compounds **1** and **2**, but not in **6**. Further, fragments of *m/z* 451.1 formed by heterocyclic ring fission in the middle unit [28] indicated a single interflavan linkage between middle and lower unit in **1** and **2**, whereas in **6**, only the fragment *m/z* 449.1 was detected, corroborating the structure of a trimeric flavan-3-ol with dihydroxylated B-ring, but two additional 2β→O→7 interflavan bonds. Due to the low yield of this substance, ^13^C NMR data could not be obtained, but the ^1^H spectrum matched that of previously published data [25]. Thus, compound **6** was tentatively identified as epicatechin-(2β→O→7, 4β→6)-epicatechin-(2β→O→7, 4β→8)-epicatechin, although the exact structure including the stereochemistry will have to be confirmed in future studies.

#### 2.1.2. Identification of Phytochemical Constituents Other than PACs

In order to characterize the phytochemical composition of the root extract beyond the active compounds (see Section 2.2), also fractions showing weak to no anthelmintic effects were further subfractionated.

**Figure 3:**
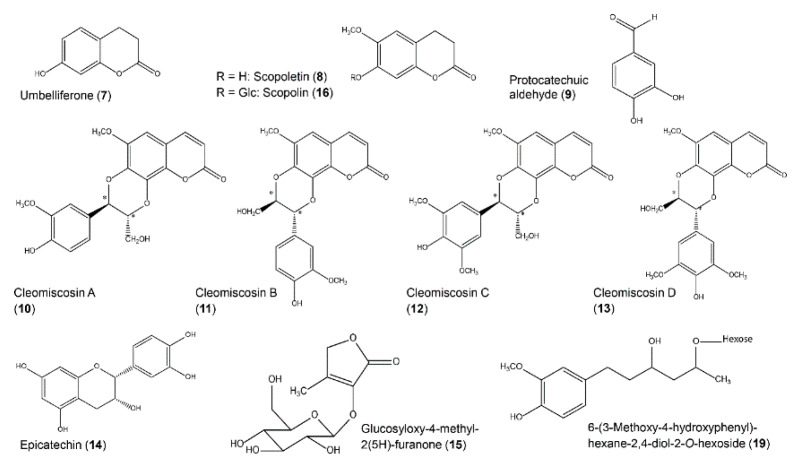
Structures of compounds **7**–**16** and **19** isolated from the acetone-water (7:3) extract of the roots of *P. pinnata*.

Fraction SE1 to SE3 obtained by separation of the EtOAc phase on Sephadex^®^ LH20 seemed to contain coumarins as indicated by strongly fluorescent zones on TLC at 365 nm. The remaining EtOAc partition was therefore fractionated by FCPC to more efficiently separate the small lipophilic constituents. Further purification of subfractions N3 to N5 by preparative HPLC afforded compounds **7**, **8** and **9** which were identified as umbelliferone, scopoletin [30,31] and protocatechuic aldehyde [32] (Figure 3). However, a few weakly fluorescent substances remained uneluted in the stationary phase together with the highly abundant oligomeric PAC. The (lower) stationary phase was therefore subjected to MPLC by flash chromatography which effectively yielded a PAC-free fraction accumulating these coumarin-like structures. As summarized in Figure 1, compounds **10** to **13** were finally isolated from F6 by preparative TLC. Interestingly, **10** and **12** as well as **11** and **13** seemed to be isomers possessing the same calculated sum formulas and UV-spectral properties respectively. *m/z* values of 417 ([M + H]^+^) for **11** and **13** versus *m/z* 387 for **10** and **12** indicated the presence of an additional methoxy group. All four substances shared the common fragment of *m/z* 207 representing a coumarin moiety [33], the corresponding neutral losses of *m/z* 180 (cmpd. **10** and **12**) and *m/z* 210 (cmpd. **11** and **13**) thus derived from a Retro-Diels Alder-like cleavage of a phenyl propanoid moiety [33] differing in one methoxy group. MS data therefore suggested compounds **10** to **13** to be the coumarinolignans cleomiscosin A, B, C and D, of which A and B as well as C and D represent structural isomers (Figure 3). Regarding the stereochemistry at position 7′ and 8′ of the molecules, coupling constants between H-7′ and H-8′ of 8 Hz indicated *trans*-configuration of the substituents. However, as previously reported [34], also the cleomiscosins from *P. pinnata* were obtained as a racemic mixture as determined by CD spectroscopy. In order to unambigously assign the correct isomeric structure, ideally, long-range correlations between H-7′ or H-8′ and C-7/C-8 resp. are observed in the HMBC spectra. Unfortunately, due to the low yield, these couplings were impossible to obtain. To increase the yield, cmpds. **10** to **13** were re-isolated from fraction SE3, but the amounts obtained were still insufficient to determine the isomeric structure directly. On the other hand, Sajeli et al. observed characteristic differences of 0.3 ppm for the ^13^C signals of C-8′ in hyosgerin and its isomer ventakasin, which were also reported for cleomiscosins A and B [34,35,36]. Similarly, the signal of C-8′ for compound **12** appeared at a lower field by δ 0.2 ppm than for **10**, whereas the signal for C-7′ appeared at a higher field by δ 0.3 ppm. Thus, **10** could be indirectly identified as cleomiscosin A and **12** as cleomiscosin B. In the same way, also the isomers cleomiscosin C and D could be discriminated [36,37,38]: The signal of C-8′ for compound **13** appeared at δ 0.3 ppm downfield compared to compound **11**, while C-7′ appeared at δ 0.4 ppm upfield, which allowed to indirectly identify **11** as cleomiscosin C and **13** as cleomiscosin D.

Along with the cleomiscosins, compound **14** was purified from SE3 and identified as epicatechin [39].

Beside the more lipophilic EtOAc phase, characteristic blue zones were also present in TLCs of the aqueous partition which was therefore subjected to fractionation on Sephadex^®^ LH20 in order to separate higher oligomeric PACs from hydrophilic small molecules. Fraction SW3 contained the major amount of fluorescent compounds, but also mono- and disaccharides in high abundance and was therefore subfractionated by MPLC column chromatography. Subsequently, this led to isolation of compounds **15** to **19** from these subfractions by preparative HPLC (Figure 1). Cmpd. **15** (*m/z* 277.1 ([M + H]^+^)) showed neutral losses characteristic of a hexose (*m/z* 162.1 and 180.1). The remaining fragments supported a furanone structure which pointed to 3-β-d-glucopyranosyloxy-4-methyl-2(5*H*)-furanone first isolated from the bug *Leptocoris isolata* [40], but also found in a methanolic extract from the stem of *P. pinnata* [41]. Since NMR spectra available were recorded in CDCl_3_ without complete assignments of sugar protons, and ^13^C-NMR data have only been published for the peracetylated compound, complete assignments for **15** were newly established for spectra recorded in D_2_O due to its excellent water solubility. A coupling constant of *J*_1,2_ 7.8 Hz confirmed β-configu-ration of the hexose. After hydrolysis using 0.1 M TFA, the monosaccharide was identified as glucose by TLC and the absolute configuration was then determined by CZE after derivatization with (S)-(−)-1-phenylethylamine. The structure of **15** was thus confirmed as 3-β-d-glucopyranosyloxy-4-methyl-2(5H)-furanone.

Cmpd. **16** was identified as scopolin by comparison of spectroscopic data with literature [42].

**Figure 4:**
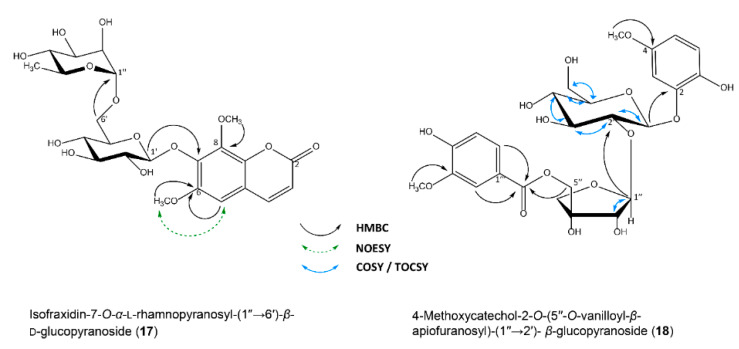
HMBC, NOESY and COSY/TOCSY key correlations for compounds **17** and **18**.

For cmpd. **17** (*m/z* 531.1775 [M + H]^+^; C_23_H_31_O_14_) also showing a UV spectrum typical for coumarins, two major fragments were detected: *m/z* 223.0643 and *m/z* 309.1214 corresponding to a dimethoxylated hydroxychromene-2-one and a disaccharide resp., of which the disaccharide consisted of a hexose and a desoxyhexose moiety. The respective monosaccharides were preliminarily identified as glucose and rhamnose by TLC after acidic hydrolysis. ^1^H NMR and COSY spectra showed two proton doublets (*δ* 7.99, d, H-4 and *δ* 6.50, d, H-3) and a singlet at *δ* 7.14 (H-5) without any COSY correlations, belonging to the chromenone residue. All remaining proton signals, including those for the methoxy groups, appeared further upfield (*δ* 5.14 to 1.00). Long-range correlations from *δ*_H_ 4.05 (3H) to *δ*_c_ 140.40 (C-8) and *δ*_H_ 3.92 (3H) to *δ*_c_ 149.86 (C-6) suggested the two methoxy groups to be linked to the aromatic moiety, however, the singlet proton at *δ* 7.14 (H-5) showed an HMBC crosspeak only to C-6, but not to C-8. Proximity of H-5 (*δ* 7.14) to the methoxy group at C-6 (*δ* 3.92) was confirmed by NOESY correlation, which additionally supported the structure of the coumarin residue to be isofraxidin. ^1^H and ^13^C NMR data for **17**, **18** and **19** are given in Table 1, key correlations are shown in Figure 4. ^1^H NMR signals of the respective carbohydrate moieties were distinguished by COSY and TOCSY spectra. The anomeric proton of the glucose moiety (*δ* 5.14, d, H-1′) correlated to position C-7 (*δ* 141.07) of the coumarin residue, whereas both protons at position 6′ of the glucose moiety (*δ* 3.92/3.73) correlated to C-1′′ (*δ* 101.37) of the rhamnose. A coupling constant of *J*_1,2_ 7.8 Hz (*δ* 5.14, d, H-1′) indicated equatorial position of the anomeric hydroxyl group and thus β-configuration of the glucose residue. A distinct three-proton singlet (*δ* 1.00, H-6′′) which could be assigned to the spin system of the rhamnose moiety by TOCSY data, was indicative for a methyl residue at position C-6′′ and pyranose form of the carbohydrate. This was supported by COSY correlations.

In case of rhamnose, axial position of the anomeric hydroxy group and thus α-configuration was deduced from the coupling constant *J*_1,2_ 1.5 Hz. Absolute configuration of the sugar residues was determined by CZE after derivatization and **17** was defined as isofraxidin-7-*O*-α-l-rhamnopyranosyl-(1″→6′)-β-d-glucopyranoside. To the best of my knowledge, **17** is a novel compound.

HR-ESIMS data of compound **18** showed an [M + H]^+^ ion at *m/z* 565.1615 corresponding to C_26_H_33_O_15_. A fragment of *m/z* 169.0506 pointed towards a methoxylated hydroxybenzoic acid (C_8_H_9_O_4_) which had been attached to a pentose moiety (*m/z* 283.0842). ^1^H NMR data revealed two AMX spin systems (*δ* 7.50, dd, H-6′′′; *δ* 7.47, d, H-2′′′; *δ* 6.78, d, H-5′′′ and *δ* 6.65, d, H-3; *δ* 6.53, d, H-6; *δ* 6.44, dd, H-5). Two singlets (*δ* 3.87, 3H and *δ* 3.74, 3H) could be assigned to two methoxy groups attached to C-3′′′ and C-4 of the aromatic rings resp. via HMBC data. Two methylene groups and a TOCSY spectrum showing a spin system of seven protons only for the hexose moiety strongly suggested an apiofuranosyl moiety as the pentose residue which was attached to C-2′ of the hexosyl group and esterified with vanillic acid at position C-5′′ (see Figure 4 for relevant HMBC correlations).

Further signals of the disaccharide were assigned by HMBC, COSY and TOCSY experiments. Coupling constants of *J*_1,2_ 1.3 Hz and *J*_1,2_ 7.5 Hz indicated β-configuration of both, the apiofuranosyl and the hexopyranosyl moiety respectively. Finally, HMBC correlations from *δ*_H_ 4.79 (H-1′) to *δ*_c_ 152.42 (C-2) revealed the linkage position of the hexosyl group to the methoxycatechol. Complete assignments of ^1^H and ^13^C NMR signals are given in Table 1. After acidic hydrolysis, the hexose was identified as glucose, but unfortunately, enzymatic sugar hydrolysis failed to yield the pentose. However, due to its characteristic features in NMR, the pentosyl moiety is assumed to be apiose. Cmpd. **18** is thus tentatively identified as 4-methoxycatechol-2-*O*-(5′′-*O*-vanilloyl-β-apiofuranosyl)-(1′′→2′)-β-glucopyranoside and represents a novel natural substance.

Finally, **19** (*m/z* 453.1392 [M + H]^+^; C_21_H_25_O_11_) fragmenting to *m/z* 241.1457 [M—hexose] ^+^ and *m/z* 163.0752, seemed to be a glycosylated hydroxyphenolic compound. Again, a three-proton singlet (*δ* 3.87, H-3′) showing a long-range correlation to an aromatic ring as indicated by an AMX spin system (*δ* 6.97, d, H-2′; *δ* 6.88, d, H-5′; *δ* 6.80, dd, H-6′) pointed to the presence of an aromatic methoxy group. A similar chemical shift in the ^13^C spectrum as observed for the methoxylated aromatic carbon (C-3′) and the absence of a signal in the HSQC spectrum suggested an additional aromatic hydroxy group at position C-4′. The HSCQ spectrum also showed the presence of four methylene groups in total, three of which appeared upfield as part of a hexyl chain and one further downfield within the carbohydrate moiety as determined via COSY, HSQC and HMBC data. The absence of a methylene group and a downfield shift of C-4 (*δ* 68.30) and C-2 (*δ* 76.09) indicated the presence of a hydroxyl group at these positions. Additionally, an HMBC correlation from the anomeric proton of the hexosyl residue to C-2 supports an *O*-glycosylation at this site. Due to the small amount of substance available, the identitiy of the hexosyl moiety as well as stereochemical properties of the compound could not be further determined. Based on the data obtained, **19** was tentatively identified as 6-(3-methoxy-4-hydroxyphenyl)-hexane-2,4-diol-2-*O*-hexoside and also represents a novel compound. Assignments of ^1^H and ^13^C NMR signals are given in Table 1. Structures of all isolated compounds other than PAC are summarized in Figure 3 and Figure 4.

### 2.2. Anthelmintic Activity of Extract and Isolated A-Type PACs

**Figure 5:**
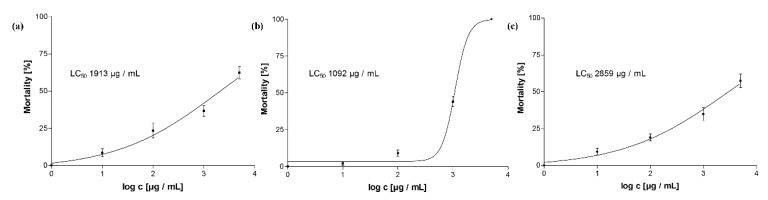
Dose-response curves of (**a**) the crude acetone-water (7:3) extract, (**b**) the ethyl actetate partition, and (**c**) the aqueous partition. Corresponding LC_50_ values as obtained by nonlinear regression are given within the respective curve.

Regarding the bioactivity, the EtOAc partition showed much stronger anthelmintic effects (LC_50_ 1.1 mg/mL) than the crude extract (LC_50_ 1.9 mg/mL) and the aqueous partition (LC_50_ 2.9 mg/mL). The respective dose-response curves are shown in Figure 5. Anthelmintic screening of fractions SE1 to SE12 obtained from fractionation of the EtOAc partition on Sephadex^®^ LH20 pointed to oligomeric PACs as the main active compounds. A mortality rate of at least 70% was observed for all PAC containing fractions at 1 mg/mL while the activity did not vary significantly among these fractions.

As one major aim of the study was not only to identify active compounds, but to determine the effects of A-type PACs in particular, cinnamtannin B1 (**1**) representing the major A-type trimer was assayed in direct comparison to procyanidin C1 (epicatechin-(4β→8)-epicatechin-(4β→8)-epicatechin), a B-type trimer previously isolated within another study [43]. Activity of the compounds was compared at a concentration of 1 mM since lethal effects were not observed in the worms at lower concentrations.

**Figure 6:**
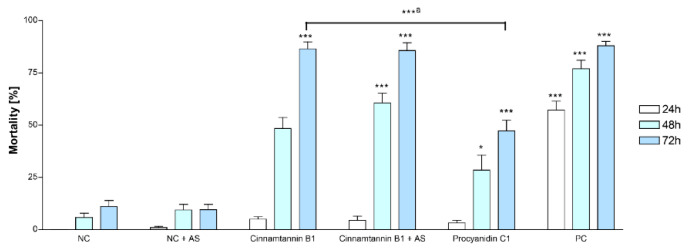
Effects of isolated cinnamtannin B1 (**1**) versus procyanidin C1 (epicatechin-(4β→8)-epicatechin-(4β→8)-epicatechin) at 1 mM on the viability of adult *C. elegans* with and without addition of 0.01% ascorbic acid (AS). NC: negative control (DMSO 1%), PC: positive control (levamisole-HCl 40 mM). Bars represent mean [%] ± SEM from at least 3 independent experiments (*n* = 12). ***: *p* < 0.001; *: *p* < 0.05 compared to NC, or „a“: compared to procyanidin C1 (One-way ANOVA, Tukey-post-test).

Interestingly, as shown in Figure 6, the mortality rate caused by the A-type PAC (**1**) was significantly higher (86.5% compared to 47.3% after 72 h) than that of the B-type trimer possessing the same sequence of epicatechin units, but unlike **1**, only single interflavan linkages between the flavan-3-ol units. In comparison, the standard anthelmintic levamisol-HCl, which was included as positive control (40 mM), caused mortality in 87.2% in the worms after 72 h.

A-and B-type PACs have been reported to easily isomerize in common aqueous assay media [44,45]. To rule out that the difference in activity was caused by a different degradation rate or formation of different degradation products, the supernatants of both test solutions were collected after the assay had been completed and subjected to LC-MS. In addition, **1** was tested with and without addition of 0.01% ascorbic acid (0.57 mM) to the test medium which reduces degradation of flavan-3-ols [46].

Fortunately, no major side products had formed during the incubation period of 72 h (Supplementary Materials Figure S2) and the addition of ascorbic acid had no impact on the anthelmintic activity of cinnamtannin B1 (**1**; Figure 6).

**Figure 7:**
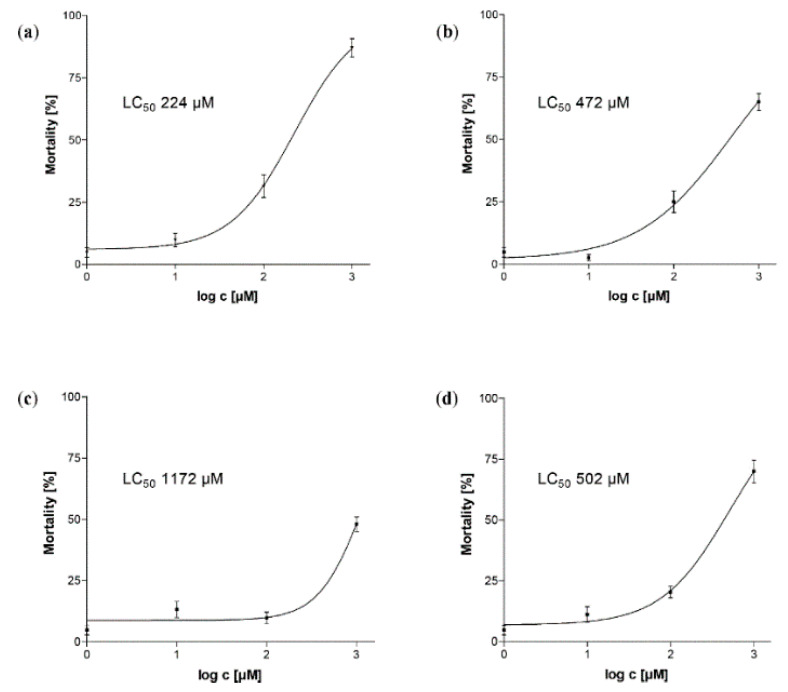
Dose-response curves of tetrameric PACs (**a**) pavetannin C1 (**3**), (**b**) epicatechin-(4β→8)-epicatechin-(2β→*O*→7, 4β→8)-epicatechin-(4β→8)-epicatechin (**4**), (**c**) parameritannin A-1 (**5**) and (**d**) cinnamtannin A2. Corresponding LC_50_ values as obtained by nonlinear regression are given within the respective curve.

As displayed in Figure 7, the anthelmintic activity of the tetrameric PAC showed a slightly different picture: Pavetannin C1 (**3**; LC_50_ 224 µM) possessing a 4β→6 linkage between the upper and the middle epicatechin unit II was superior compared to the other tested PACs. On the other hand, parameritannin A-1 (**5**; LC_50_ 1172 µM) which is a ”branched“ A-type tetramer with an epicatechin unit linked 4β→6 to unit II (Figure 2) was markedly less effective than the other tetramers. Unlike the trimers, the tetrameric B-type procyanidin cinnamtannin A2 [43] was not inferior compared to the A-type PACs, showing a similar LC_50_ value like **4** (LC_50_ 502 µM vs. 472 µM, resp.).

## 3. Discussion

Aim of the current study was to phytochemically characterize the acetone-water (7:3) extract from the roots of *P. pinnata* and to reveal the compounds responsible for the anthelmintic activity.

Proanthocyanidins of the A-type were the predominant class of secondary metabolites within the extract and also represented the major bioactive class of compounds. In particular, the tetrameric PACs pavetannin C1 (**3**) and epicatechin-(4β→8)-epicatechin-(2β→*O*→7, 4β→8)-epicatechin-(4β→8)-epicatechin (**4**) showed the strongest anthelmintic activity. Generally, in line with previous findings, oligomeric PACs present in the EtOAc partition, showed superior nematicidal effects compared to polymeric structures of the aqueous partition (for review see [47]).

With respect to their anthelmintic activity, A-type PACs have not yet been explored in detail. Except for one study reporting in vitro anthelmintic effects of an extract from cinnamon bark containing 21% A-type PACs beside the more common B-types against *Ascaris suum* [48], and previous investigations of a root extract from *P. pinnata* [8], this is one of the only studies directly comparing the anthelmintic activity of PAC molecules of the same flavan-3-ol composition, but different types of interflavan linkages (i.e., 4β→6 vs. 4β→8 linkages, additional 2β→*O*→7 linkages, a “branched” sequence of flavan-3-ol units).

Similar to the current findings for the trimeric PACs, a purified fraction of oligomeric PACs from a cranberry extract containing predominantly A-types has been reported to agglutinate extra-intestinal pathogenic *E. coli* and to inhibit bacterial invasion to a significantly higher extent than a fraction of purified B-type PACs from apples. The molecular size of the oligomers was roughly equally distributed in both fractions [49]. Other structural features such as (4β→6) versus (4β→8) linkages or the position of the additional (2β→*O*→7) bond could not be assessed. In addition to an improved antiadhesive activity in vitro of a cranberry juice compared to several food products containing mainly B-type PACs, the observed effects remained also superior when human urine samples were assayed after consumption of the respective foods or beverages [50]. On the other hand, regarding antioxidant or membrane protective effects, the difference in the activity of the two types of PAC is not as clear [51,52].

No significant difference was found for tetrameric A-type vs. B-type PACs. On the other hand, similar to this study, Kiuichi et al. observed a superior activity of fatty acids when combined with 4β→6 linked B-type procyanidins from *Areca catechu* compared to the 4β→8 linked derivatives against larvae of the dog roundworm *Toxocara canis*. Also, the A-type trimer enhanced the anthelmintic effect of decanoic acid, whereas the B-types were inactive [53]. Why a trimeric A-type PAC with a more rigid scaffold is more active than the more flexible B-types, whereas linear 4β→6 linkages cause a higher mortality than 4β→8 linkages, despite a less hindered rotation around the 4→6 bond, remains speculative. The weakest effect among the tetramers was surprisingly found for parameritannin A-1, despite its branched 4β→6 linked unit and an A-type linkage. Beside the degree of polymerization and the number of B-ring hydroxyl groups, the interflavan linkage seems to have a major impact on the anthelmintic activity of PACs. The current study further underlines the importance to perform studies with isolated compounds in order to consider the role of the interflavan linkages in structure-activity-relationships of PACs.

With the exception of one *ent*-catechin unit (**2**), all PACs were composed of epicatechin as the monomeric flavan-3-ol. Given that epicatechin has been found to isomerize to *ent*-catechin ((−)-catechin) in hot aqueous solutions by inversion of the aryl group at position C-2 [54,55], it seems likely that the small proportion of **2** compared to **1** results from an epimerization of **1** at C-2 during the extraction or purification procedure. Due to their readiness to isomerize also in aqueous culture media [44,45], the stability of cinnamtannin B1 (**1**) was assessed after the incubation period of 72 h. Fortunately, the degradation rate in M9 buffer at ambient temperature was by far not as high as described by Lu et al. [44].

Regarding the coumarins, due to their high structural variability, a plethora of bioactivities have been described, ranging from antimicrobial, antiviral, antiproliferative to anti-inflammatory, analgesic and anti-hypertensive effects [56]. These activities have mainly been ascribed to the aglyca. The two non-glycosylated coumarins found in the *P. pinnata* root extract, umbelliferone and scopoletin, are simple coumarins which can ubiquitously be found throughout the plant kingdom [57]. Beside anti-inflammatory, anti-proliferative and antioxidant activities [56] both compounds also exterted moderate antibacterial effects in vitro [56,58]. In plants, the main functions of these coumarins involve the aquisition of ferric iron from the environment and the defense against phyto-pathogens [59].

Also the coumarinolignans are widely distributed among plant families, including different species of Sapindaceae [60]. Similar to simple coumarins, cleomiscosins A to D are as well secreted in response to iron deficiency at high pH [61]. Further biological activities with respect to their potential medicinal use include liver protection, vasorelaxation, anti-inflammatory, antiviral, immunmodulatory and lipid peroxidation inhibiting effects [60]. A cytotoxic activity is reported for one cell line, but has not been confirmed in others [60].

The furanone glucoside (**15**) which has previously been found in the roots of *P. pinnata* [41] has originally been isolated from the Neo-Guinean bug *Leptocoris isolata* and was speculated to be involved in the defense of the bug against ants [40]. Lunga et al. demonstrated antibacterial and antifungal effects of this compound against selected gram-negative bacteria, yeasts and dermatophytes [41]. β-miroside, a 3-hydroxymethyl-2(5*H*) furanone glucoside from *Prumnopitys ferruginea* was additionally reported to exert cytotoxicity against monkey kidney cells [62], ranunculin and siphenoside, varying in the position of the glucosylated hydroxymethyl chain, showed similar cytotoxic effects [62,63]. On the other hand, psydrin (5-methyl-4-hydroxy-3(2*H*)furanone glucoside), a rare structural isomer of **15** found in the leaves of *Psydrax livida* [64] and *Juniperus phaenicea* [65] was found to be non toxic against human tumoral and leucemic cell lines [65].

Hydroxybenzoic acid derivatives frequently occur in plants in small amounts, mainly as glycosides or esters to function in microbial defense and growth regulation [66]. Given the structural variability of hydroxybenzoic acids and phenolic natural products, the number of possible bonds with mono- and disaccharides is accordingly high. Compound **18** ist thus the result of vanillic acid, apiosyl glucose and methoxycatechol in a novel arrangement, similar to the milletiaspecosides [67], reevesianin B [68] or seguinoside K [69].

Also glycosides connected to a phenylalkyl residue are generally widespread among different plant families. The majority of these compounds comprises phenylethanoids consisting of an aromatic C_6_ moiety usually substituted with at least one hydroxyl or methoxy group, and an aliphatic C_2_ side chain [70,71]. Biosynthesis occurs via tyrosine/tyramine as precursors of tyrosol [72] which is then glycosylated at the ethyl hydroxyl group. A variety of in vitro effects has been described for phenylethanoids, most frequently antioxidant, anti-inflammatory, but also cytotoxic [71] and antibacterial properties [70]. Similar activities have also been reported for arylbutanoids [73,74] and their glycosides as well as diarylheptanoids [75,76]. Phenylbutanoids which occur much less frequently in plants than phenylethanoids, were first isolated from *Rhododendron chrysanthemum* (syn. *R. aureum*) [77] and later found mainly in rhododendron [78,79] and betula species [75,80,81,82,83], however, their occurence is not limited to these genera [74,84,85,86,87]. Biosynthetically, these stuctures are derived from a cinnamic acid derivative which is subsequently reduced to a dihydrocinnamoyl alcohol. Finally, an additional methyl group is transferred enzymatically from SAM [88]. Similar to the biosynthesis of diarylheptanoids, e.g., acerogenin A [89], the curcuminoids [90,91,92] or gingerols [91,92,93], the aglycon of cmpd. **19** could be biosynthesized from either ferulic or cinnamic acid derived from phenylalanine, which is linked to malonic acid in the first step [89,90,91,93]. While in gingerols, this intermediate subsequently condenses with a fatty acid of a C_6_ to C_10_ chain length, a likewise reaction with acetic acid would form cmpd. **19** from the same intermediate. In addition, the respective glycosides could function as intermediates as hypothesized for 6-gingerdiol glucosides possessing a structure very similar to **19** [94]. To my knowledge, only one phenylhexanoid found in ginger [95] has been described at all so far, the hypothesized biosynthesis therefore needs further experimental confirmation.

None of the compounds other than PAC, or the fractions containing the resp. compounds, contributed to the anthelmintic activity. On the other hand, anti-inflammatory, anti-proliferative and particularly antimicrobial effects have most frequently been reported for these structures.

While the ethnopharmacological use of the roots against nausea and vomiting [4], cannot be directly correlated to the in vitro data obtained for the respective compound classes, the presence of PAC could be responsible for the traditional use of root preparations from *P. pinnata* as a styptic while an anti-inflammatory activity could be beneficial against eczema [4].

## 4. Materials and Methods

### 4.1. Plant Materials and Chemicals

Roots from *Paullinia pinnata* L. were collected between April and June 2014 from the Bosomtwi-Atwima-Kwanwoma area in the Ashanti region of Ghana, located between 0.15–2.25 °W and 5.50–7.46 °N. After botanical identification, a voucher specimen (IPBP No. 328) has been deposited in the herbarium of the Institute of Pharmaceutical Biology and Phytochemistry, University of Münster, Germany.

If not stated otherwise, all chemicals were purchased from VWR.

### 4.2. General Analytical Techniques

Analytical thin layer chromatography (TLC) was generally performed on silica gel plates 60 F_254_ (0.2 mm; Merck, Darmstadt, Germany) using ethyl acetate/water/formic acid (90:5:5 *v*/*v*/*v*) as the mobile phase. Compounds were visualized under UV-light (254 nm or 365 nm resp.) and at daylight after spraying with anisaldehyde/sulphuric acid reagent followed by heating the plate to approx. 105 °C, or with vanillin-HCl reagent. Mono- and disaccharides were analyzed using n-propanol/water/ethanol (7:2:1 *v*/*v*/*v*) as mobile phase and detected at daylight after derivatization with thymol/sulphuric acid reagent after heating to 105° C.

Acquity™ Ultra Performance LC, PDA λe Detector and QDa™ Detector, autosampler, in-line degasser, and Waters Empower 3^®^ Software (Waters, Milford, MA, USA) were used for analytical UPLC.

Separation was performed on a RP-18 stationary phase (HSS T3, 1.8 μm, 2.1 × 100 mm) using a binary gradient of 0.1% formic acid (A) and acetonitrile/0.1% formic acid (B) at 0.5 mL/min.

Analysis by UPLC-qTOF-MS was carried out as follows: Separation was performed on a Dionex Ultimate 3000 RS Liquid Chromatography System over a Dionex Acclaim RSLC 120, C18 column (2.1 × 100 mm, 2.2 µm) with a binary gradient (A: water with 0.1% formic acic; B: acetonitrile with 0.1% formic acid) at 0.4 mL/min: *t*_0min_ 5% B, *t*_0.4min_ 5% B, *t*_9.9min_ 100% B, *t*_15min_ 100% B, *t*_15.1min_ 5% B, *t*_20min_ 5% B. The injection volume was 2 µL. Eluted compounds were detected using a Dionex Ultimate DAD-3000 RS over a wavelength range of 200–400 nm and a Bruker Daltonics micrOTOF-QII time-of-flight mass spectrometer equipped with an Apollo electrospray ionization source in positive or negative mode at 3 Hz over a mass range of *m/z* 50–1500 or *m/z* 300–3000 using the following instrument settings: nebulizer gas nitrogen, 3.5 bar; dry gas nitrogen, 9 L/min, 200 °C; capillary voltage 4500 V; end plate offset −500 V; transfer time 100 µs, prepulse storage 6 µs; collision energy and collision RF settings were combined to each single spectrum of 2500 summations. MS/MS scans were triggered by AutoMS2 settings within a range of *m/z* 200–1500 or 500–3000 resp. Internal dataset calibration (HPC mode) was performed for each analysis using the mass spectrum of a 10 mM solution of sodium formiate in isopropanole-water-formic acid-1M NaOH solution (50 + 50 + 0, 2 + 1), that was infused during LC reequilibration using a divert valve equipped with a 20 µL sample loop.

NMR spectra were recorded on an Agilent DD2 spectrometer (Agilent Technologies, Santa Clara, USA) at 600 MHz (^1^H) or 150 MHz (^13^C). Depending on their solubility, samples were dissolved in chloroform-*d*_1_ (7.26; 77.16 ppm), methanol-*d*_4_ (3.31; 49.00 ppm), deuterium oxide (4.79 ppm) or acetone-*d*_6_ (2.84; 206.26 ppm) and chemical shifts were referenced to the respective residual solvent signals (^1^H and ^13^C shifts in brackets). In case of D_2_O, ^13^C spectra were referenced to the signal of methanol (49.00 ppm) which was added in low amounts to the sample. Spectra were recorded at 299 K, except for compounds **1** to **6** which were recorded at 280 K due to a better resolution and sharper signals at lower temperature [27,96].

**3**, possessing a 4→6 interflavan linkage, had to be measured as its peracetate to obtain interpretable data of signal splitting in ^1^H NMR. Peracetylation of **3** was peformed in pyridine/acetic acid anhydride (1:1) at room temperature for 24 h in the dark [97].

CD spectra were recorded between 190 and 400 nm on a Jasco CD-spectropolarimeter J-815. Samples were dissolved in methanol to 1 mM, further diluted to final concentrations from 125 µM to 670 µM and measured in a quartz cell with a path length of 0.1 cm.

### 4.3. Preparation of the Plant Extract

1.6 kg powdered root material were sequentially defatted by Soxhlet extraction in petroleum ether for 4 h (yield: 4 g) followed by dichloromethane for 5 h (yield: 3.5 g). 1.2 kg of the remaining material were successively extracted three times with acetone/water (7:3 *v*/*v*) in a drug-solvent ratio of 1:10 by Ultra-Turrax^®^ (IKA, Staufen, Germany) at 9500 rpm for 10 min under ice cooling. The suspension was centrifuged at 3000× *g* for 10 min, concentrated *in vacuo* and lyophilized. The crude extract (yield: 150.2 g; 12.5% related to the dried plant material) and all fractions obtained from the extract by the following fractionation were stored at −20 °C.

### 4.4. Fractionation of the Acetone-Water (7:3) Extract

A portion of the acetone-water extract (95 g) was partitioned between ethyl acetate (EtOAc) and water by dissolving portions of 15 g of extract in 500 mL of water and extracting three times with 500 mL of EtOAc. The aqueous and organic phases were filtered (filter paper 595, S & S, Dassel, Germany) and lyophilized. Yield: 9.3 g of the EtOAc phase and 161 g of the aqueous phase, corresponding to 10.2% and 72.9% of the crude extract respectively.

#### 4.4.1. Fractionation on Sephadex^®^ LH20

4 g of the ethyl acetate partition were fractionated on Sephadex^®^ LH-20 (General Electrics, Munich, Germany) using a column size of 665 × 30 mm i.d. and a step gradient of EtOH (6.5 L), MeOH (1 L) and acetone-H_2_O (3.5 L, 7:3 *v*/*v*), fraction size 15 mL. Every third fraction was monitored by TLC and fractions with comparable composition were combined to afford fractions SE1–SE12.

In addition, the aqueous partition (30 g) was fractionated on Sephadex^®^ LH-20 using a column size of 350 × 70 mm i.d. and a step gradient of EtOH (1.4 L), MeOH-H_2_O (2.9 L, 1:1 *v*/*v*), MeOH (8 L) and acetone-H_2_O (3 L, 7:3 *v*/*v*), fraction size 15 mL. Every third fraction was monitored by TLC and fractions with comparable composition were combined to afford fractions SW1–SW10.

#### 4.4.2. Fast Centrifugal Partition Chromatography

2.5 g of the lyophilized EtOAc partition were further fractionated in portions of 500 mg by Fast Centrifugal Partition Chromatography (FCPC) (Kromaton, Kromaton Technologies, Angers, France), mobile phase: EtOAc/hexane 1:1 (*v*/*v*), stationary phase: MeOH/H_2_O 1:1 (*v*/*v*); ascending mode, flow 5 mL/min; 1210 rpm; fraction size 10 mL). The fractions obtained were investigated by TLC and fractions with similar composition were combined to fractions N1 to N6.

#### 4.4.3. Medium Pressure Liquid Chromatography (MPLC)

Fraction N6 (500 mg) corresponding to the extruded stationary phase (FCPC), was further fractionated by Flash Chromatography (SPOT Liquid chromatography Flash, Armen Instrument, Saint-Avé, France) using an SVF D26-RP18 column (30 g; 25–40 µm particle size) and a gradient of water (A) and methanol (B): *t*_0min_ 5% B, *t*_65min_ 100% B, *t*_75min_ 100%; flow rate 8 mL/min, fraction size 5 mL. Each fraction was monitored by TLC and fractions with similar composition were combined to afford fractions F1–F8.

4.5 g of fraction SW3 were further fractionated in portions of 1.5 g by MPLC on an RP-18 stationary phase (RP-18, 18–32 µm, 100 Å, 36 × 500 mm (BESTA Technik, Wilhelmsfeld, Germany), flow 8 mL/min, step gradient MeOH 5% (30 min), MeOH 10% (30 min), MeOH 30% (1 h), MeOH 50% (1 h), MeOH (1 h), fraction size 16 mL). Every second fraction was monitored by TLC and fractions with comparable composition were combined to afford fractions M1−M7.

#### 4.4.4. Preparative TLC

Fraction F6 (8 mg) were dissolved in 1 mL acetone and applied to a silica gel TLC plate (60 F₂₅₄, 0.5 mm; Merck, Darmstadt, Germany. The plate was developed in EtOAc/H_2_O/HCOOC (98:1:1 *v*/*v*/*v*) over a distance of 17 cm and fluorescent zones detected at 366 nm were removed and collected. Compounds were desorbed from silica gel in 15 mL acetone per sample and shaking for 1.5 h at 120 rpm (GFL Reciprocating Shaker 3018, Gesellschaft für Labortechnik, Burgwedel, Germany). The suspensions were then centrifuged (8000 rpm, 10 min) and the supernatants were dried under reduced pressure to yield compounds **10** to **13** (yield: 1.5 mg; 0.5 mg; 0.2 mg and 0.2 mg respectively).

#### 4.4.5. Preparative HPLC

Preparative HPLC was generally carried out using Quaternary Gradient Module 2545, Photodiode Array Detector 2998, Autosampler 2707, Waters Prep Degasser and Waters Fraction Collector III. Software: Waters ChromScope v1.40 Beta (Waters, Milford, MA, USA). Stationary phase: Nucleodur^®^ C18 HTec, 5 µm, 250 × 21 mm, mobile phase: binary gradient of water (A) and acetonitrile (B) at a flow rate of approx. 15.5 mL/min.

Separation of fraction SE6 yielded **1** (118 mg) and **2** (1.2 mg). **3** (11 mg) was obtained from SE7, **4** (5.3 mg), **5** (13 mg) and **6** (0.65 mg) from fraction SE8. Subfractionation of MPLC fraction M2 and M3 led to **15** (39 mg). **16** (0.5 mg) was obtained from M4, and M5 yielded **17** (0.6 mg), **18** (0.9 mg) and **19** (0.3 mg).

Fractions N3 to N5 obtained from FCPC and SE3 were further purified using the following system: Two Waters 515 HPLC Pumps, Waters Pump Control Module II, Degasys DG-2410 (Uniflows, Tokyo, Japan), Waters 996 PDA Detector, software: Waters ChromScope v1.40 Beta Software (Waters, Milford, MA, USA), stationary phase: Eurospher 100 C18, 250 × 21 mm; 7 µm (VDS Optilab, Germany), mobile phase gradient: water (A), acetonitrile (B), flow: 10 mL/min, injection volume: 1 mL. **7** (0.12 mg), **8** (0.9 mg) and **9** (1.7 mg) were isolated from N3, N5 and N4 resp. Compound **14** (11 mg) was isolated from SE3 along with **10** (3.9 mg), **11** and **12** as a mixture (4.3 mg) and **13** (2.7 mg).

### 4.5. Hydrolysis of Glycosides

Mono- and disaccharides were hydrolysed as described by Albersheim et al. [98]. Trifluoroacetic acid (TFA) was removed by at least three washing steps with 2 mL MeOH 50%. Hydrolysis of **18** was performed enzymatically as described by [99]. Hydrolysed sugars were identified by TLC compared to reference compounds.

### 4.6. Capillary Zone Electrophoresis (CZE)

The carbohydrates for CZE electrophoresis were derivatized as described by Noe and Freissmuth [100]. CZE equipment: Beckman Coulter P/ACE MDQ (Beckman Coulter, Brea, USA); fused silica capillary, 70/77 cm × 50 μm i.d.; running buffer, 50 mM Na_2_B_2_O_7_ pH 10.3, MeCN 4.4 mol/L added; injection, 5−10 s at 0.5 psi; voltage, 30 kV; detection, λ = 200 nm; software, 32 Karat software version 5.0 (Beckman Coulter).

### 4.7. Identification of Isolated Compounds

Compounds were identified by their spectroscopic and ESI-MS data, and known compounds were compared to previously published data. For **15** and novel compounds **17**, **18** and **19,** all NMR spectra relevant for structure elucidation were given as Supplementary Materials Figures S3–S6. For known compounds (**1** to **16**) ^1^H NMR spectra were given as Supplementary Materials Figures S7–S22.

Cinnamtannin B1 (**1**), epicatechin-(2β→*O*→7, 4β→8)-epicatechin-(4β→8)-epicatechin): off-white amorphous powder. CD (MeOH) *λ*_max_ [nm] (Δ*ε*): 210 (−37.48), 230 (+18.62), 270 (−2.25). ESITOFMS *m/z* 863.1813 [M − H]^−^ (calcd for C_45_H_35_O_18_, 863.1829). ^1^H and ^13^C NMR see Supplementary Materials Table S1.
Aesculitannin B (**2**), epicatechin-(2β→*O*→7, 4β→8)-*ent*-catechin-(4β→8)-epicatechin): off-white amorphous powder. CD (MeOH) *λ*_max_ [nm] (Δ*ε*): 208 (−34.56), 234 (+37.16), 273 (−1.72). ESITOFMS *m/z* 863.1860 [M − H]^−^ (calcd for C_45_H_35_O_18_, 863.1829). ^1^H and ^13^C NMR see Supplementary Materials Table S1.
Pavetannin C1 (**3**), epicatechin-(4β→6)-epicatechin-(2β→*O*→7, 4β→8)-epicatechin-(4β→8)-epi- catechin. Light brown amorphous powder. CD (MeOH) *λ*_max_ [nm] (Δ*ε*): 208 (−114.87), 234 (+37.16), 278 (−8.85). ESITOFMS *m/z* 1153.2819 [M + H]^+^ (calcd for C_60_H_49_O_24_, 1153.2608). ^1^H and ^13^C NMR after peracetylation (**3a**) see Supplementary Materials Table S2.
Epicatechin-(4β→8)-epicatechin-(2β→*O*→7, 4β→8)-epicatechin-(4β→8)-epicatechin (**4**), off-white amorphous powder. CD (MeOH) *λ*_max_ [nm] (Δ*ε*): 205 (−29.70), 234 (+33.51), 274 (−5.05). ESITOFMS *m/z* 1151.2468 [M − H]^−^ (calcd for C_60_H_47_O_24_, 1151.2463). ^1^H and ^13^C NMR see Supplementary Materials Table S2.
Parameritannin A-1 (**5**), epicatechin-(2β→*O*→7, 4β→8)-[epicatechin-(4β→6)]-epicatechin- (4β→8)-epicatechin): off-white amorphous powder. CD (MeOH) *λ*_max_ [nm] (Δ*ε*): 212 (−33.14), 235 (+22.77), 279 (−2.06). ESITOFMS *m/z* 1151.2605 [M − H]^−^ (calcd for C_60_H_47_O_24_, 1151.2463). ^1^H and ^13^C NMR see Supplementary Materials Table S2.
Epicatechin-(2β→*O*→7, 4β→6)-epicatechin-(2β→*O*→7, 4β→8)-epicatechin (**6**), off-white amorphous powder. ESITOFMS *m/z* 861.1719 [M − H]^−^ (calcd for C_45_H_33_O_18_, 863.1672). ^1^H NMR (methanol-*d*4) *δ*: 7.43 (dd, *J* = 9.1, 2.2 Hz, 1H; H-6′ (E)), 7.41 (d, *J* = 2.2 Hz, 1H; H-2′ (E)), 7.17 (d, *J* = 2.0 Hz, 1H; H-2′ (H)), 7.09 (d, *J* = 2.1 Hz, 1H; H-2′ (B)), 7.03 (dd, *J* = 8.3, 2.1 Hz, 1H; H-6′ (H)), 6.98 (dd, *J* = 8.5, 2.3 Hz, 1H; H-6′ (B)), 6.88 (d, *J* = 8.1 Hz, 1H; H-5′ (E), 6.82 (d, *J* = 8.2 Hz, 1H; H-5′ (H)), 6.78 (d, *J* = 8.4 Hz, 1H; H-5′ (B)), 6.16 (s, 1H; H-8 (D)), 6.09 (s, 1H; H-6 (G)), 6.06 (d, *J* = 2.4 Hz, 1H; H-8 (A)), 6.02 (d, *J* = 2.4 Hz, 1H; H-6 (A)), 4.68 (d, *J* = 2.9 Hz, 1H; H-4 (A)), 4.53 (d, *J* = 3.0 Hz, 1H; H-4 (F)), 4.26 (m, 1H; H-3 (I)), 4.10 (d, *J* = 3.0 Hz, 1H; H-3 (F)), 3.98 (d, *J* = 3.1 Hz, 1H; H-3 (C)), 2.96 (dd, *J* = 17.0, 4.6 Hz, 1H; H-4a (F)), 2.82 (dd, *J* = 17.9, 1.6 Hz, 1H; H-4b (F)).
Umbelliferone (**7**), brownish yellow amorphous powder. UV (MeCN, H_2_O) λ_max_ 220, 324. ESITOFMS *m/z* 163.0400 [M + H]^+^ (calcd for C_9_H_7_O_3_, 163.0390). ^1^H NMR (chloroform-*d*) *δ*: 7.63 (d, *J* = 9.5 Hz, 1H; H-4), 7.36 (d, *J* = 8.3 Hz, 1H; H-5), 6.80 (d, *J* = 2.3 Hz, 1H; H-8), 6.78 (dd, *J* = 8.3, 2.4 Hz, 1H; H-6), 6.26 (d, *J* = 9.5 Hz, 1H; H-3). Data were identical to those of a reference substance (Carl Roth, Karlsruhe, Germany).
Scopoletin (**8**), yellow amorphous powder. UV (MeCN, H_2_O) λ_max_ 203, 227, 296s, 344. ESITOFMS *m/z* 193.0490 [M + H]^+^ (calcd for C_10_H_9_O_4_, 193.0495). ^1^H NMR (chloroform-*d*) *δ*: 7.60 (d, *J* = 9.2 Hz, 1H; H-4), 6.92 (s, 1H; H-8)), 6.85 (s, 1H; H-5), 6.27 (d, *J* = 9.5 Hz, 1H; H-3), 6.12 (s, 1H; 7-OH), 3.96 (s, 3H; 6-OCH_3_). ^13^C NMR (chloroform-*d*) *δ*: 161.51 (C-2), 150.32 (C-7), 149.87 (C-9), 144.06 (C-6), 143.25 (C-4), 113.42 (C-3), 111.60 (C-10), 107.44 (C-5), 103.27 (C-8), 56.43 (6-OCH_3_).
Protocatechuic aldehyde (**9**), light brown amorphous powder. UV (MeCN, H_2_O) λ_max_ 205, 230, 280, 311. ESITOFMS *m/z* 139.0378 [M + H]^+^ (calcd for C_7_H_7_O_3_, 139.0390). ^1^H NMR (methanol-*d*_4_) *δ*: 9.67 (s, 1H; CHO), 7.30 (dd, *J* = 8.0, 1.9 Hz, 1H; H-6), 7.29 (d, *J* = 1.9 Hz, 1H; H-2), 6.89 (d, *J* = 8.0 Hz, 1H; H-5). ^13^C NMR (methanol-*d*_4_) δ: 193.00 (CHO), 154.54 (C-4), 147.40 (C-3), 130.45 (C-1), 126.63 (C-6), 116.30 (C-5), 115.09 (C-2).
Cleomiscosin A (**10**), pale yellow amorphous powder. UV (MeCN, H_2_O) λ_max_ 206, 235s, 283s, 328. ESITOFMS *m/z* 387.1072 [M + H]^+^ (calcd for C_20_H_19_O_8_, 387.1074). ^1^H NMR (acetone-*d*_6_) *δ*: 7.88 (d, *J* = 9.5 Hz, 1H; H-4), 7.81 (s, 1H; OH-4′), 7.16 (d, *J* = 1.9 Hz, 1H; H-2′), 7.01 (dd, *J* = 8.1, 2.0 Hz, 1H; H-6′), 6.91 (d, *J* = 8.1 Hz, 1H; H-5′), 6.84 (s, 1H; H-5), 6.27 (d, *J* = 9.5 Hz, 1H; H-3), 5.10 (d, *J* = 8.0 Hz, 1H; H-7′), 4.25 (m, 1H; H-8′), 3.89 (overlapped with –OCH_3_; H-9′a) 3.88 (s, 3H; 3′-OCH_3_), 3.84 (s, 3H; 6-OCH_3_), 3.57 (m, 1H; H-9′b). ^13^C NMR (acetone-*d*_6_) *δ*: 160.93 (C-2), 148.63 (C-3′), 148.38 (C-4′), 146.97 (C-6), 145.22 (C-4), 139.77 (C-9), 138.72 (C-7), 133.29 (C-8), 128.51 (C-1′), 121.93 (C-6′), 115.96 (C-5′), 114.42 (C-3), 112.53 (C-10), 112.29 (C-2′), 101.79 (C-5), 79.60 (C-8′), 77.64 (C-7′), 61.55 (C-9′), 56.60 (3‘-OCH_3_), 56.46 (6-OCH_3_).
Cleomiscosin C (**11**), pale yellow amorphous powder. UV (MeCN, H_2_O) λ_max_ 210, 235s, 327. ESITOFMS *m/z* 417.1189 [M + H]^+^ (calcd for C_21_H_21_O_9_, 417.1180). ^1^H NMR (acetone-*d*_6_) *δ*: 7.88 (d, *J* = 9.6 Hz, 1H; H-4), 7.43 (s, 1H; OH-4′), 6.87 (s, 2H; H-2′/H-6′), 6.85 (s, 1H; H-5), 6.27 (d, *J* = 9.5 Hz, 1H; H-3), 5.08 (d, *J* = 8.0 Hz, 1H; H-7‘), 4.37 (s, 1H; OH-9′), 4.28 (ddd, *J* = 8.0, 3.6, 2.4 Hz, 1H; H-8‘), 3.90 (ddd, *J* = 12.4, 5.2, 2.4 Hz, 1H; H-9‘a), 3.86 (s, 6H; 3‘/5‘-OCH_3_), 3.84 (s, 3H, 6-OCH_3_), 3.58 (m, 1H; H-9‘b). ^13^C NMR (acetone-*d*_6_) *δ*: 160.96 (C-2), 149.00 (C-3‘/5‘), 147.01 (C-6), 145.25 (C-4), 139.75 (C-9), 138.76 (C-7), 138.62 (C-8) 137.76 (C-4‘), 133.37 (C-8), 114.49 (C-3), 112.50 (C-10), 127.35 (C-1‘), 106.57 (C-2‘/6‘), 101.84 (C-5), 79.59 (C-8‘), 77.96 (C-7‘), 61.62 (C-9‘), 56.88 (3‘/5‘-OCH_3_), 56.66 (6-OCH_3_).
Cleomiscosin B (**12**), pale yellow amorphous powder. UV (MeCN, H_2_O) λ_max_ 204, 235s, 280s, 328. ESITOFMS *m/z* 387.1080 [M + H]^+^ (calcd for C_20_H_19_O_8_, 387.1074). ^1^H NMR (acetone-*d*_6_) *δ*: ^1^H NMR (600 MHz, acetone-*d*_6_) δ 7.87 (d, *J* = 9.6 Hz, 1H; H-4), 7.80 (s, 1H; OH-4′), 7.17 (d, *J* = 2.0 Hz, 1H; H-2′), 7.02 (dd, *J* = 8.0, 2.0 Hz, 1H; H-6′), 6.90 (d, *J* = 8.1 Hz, 1H; H-5′), 6.86 (s, 1H, H-5), 6.23 (d, *J* = 9.5 Hz, 1H; H-3), 5.10 (d, *J* = 8.0 Hz, 1H; H-7′), 4.28 (ddd, *J* = 7.9, 3.7, 2.5 Hz, 1H; H-8′), 3.90 (s, 3H, 6-OCH_3_) 3.89 (overlapped with -OCH_3_; H-9′a), 3.88 (s, 3H; 3′-OCH_3_), 3.57 (m, H-9′b). ^13^C NMR (acetone-*d*_6_) *δ*: 160.83 (C-2), 148.71 (C-3′), 148.60 (C-4′), 147.09 (C-6), 145.15 (C-4), 138.59 (C-7), 139.91 (C-9), 139.84, 138.76, 137.78, 133.37 (C-8), 128.54 (C-1′), 121.97 (C-6′), 116.00 (C-5′), 114.48 (C-3), 112.45 (C-10), 112.29 (C-2′), 102.03 (C-5), 79.77 (C-8′), 77.28 (C-7′), 61.71 (C-9′), 56.72 (6-OCH_3_), 56.50 (3′-OCH_3_).
Cleomiscosin D (**13**), pale yellow amorphous powder. UV (MeCN, H_2_O) λ_max_ 210, 235s, 331. ESITOFMS *m/z* 417.1193 [M + H]^+^ (calcd for C_21_H_21_O_9_, 417.1180). ^1^H NMR (acetone-*d*_6_) *δ*: ^1^H NMR (acetone-*d*_6_) *δ*: 7.87 (d, *J* = 9.5 Hz, 1H; H-4), 6.87 (s, 2H; H-2′/H-6′), 6.87 (s, 1H; H-5), 6.24 (d, *J* = 9.5 Hz, 1H; H-3), 5.08 (d, *J* = 7.9 Hz, 1H; H-7′), 4.30 (m, 1H; H-8′), 3.89 (s, 3H; 6-OCH_3_), 3.88 (overlapped with OCH_3_; H-9′a), 3.86 (s, 6H; 3‘/5‘-OCH_3_), 3.57 (m, 1H, H-9′b). ^13^C NMR (acetone-*d*_6_) *δ*: 159.76 (C-2), 151.18, 149.02 (C-3′/C-5′), 146.59 (C-6), 147.10, 146.84, 145.19 (C-4), 139.88 (C-9), 138.58 (C-7), 137.75 (C-4′), 134.01 (C-8), 127.29 (C-1′), 114.45 (C-3), 111.94 (C-10), 108.36, 106.56 (C-2′/C-5′), 102.05 (C-5), 79.88 (C-8′), 77.55 (C-7′), 61.58 (C-9′), 56.87 (3‘/5‘-OCH_3_), 56.71 (6-OCH_3_).
Epicatechin (**14**), white amorphous powder. UV (MeCN, H_2_O) λ_max_ 220, 278. ESITOFMS *m/z* 291.0867 [M + H]^+^ (calcd for C_15_H_15_O_6_, 291.0863). ^1^H NMR (methanol-*d*_4_) δ 6.97 (d, *J* = 1.9 Hz, 1H; H-2′), 6.80 (dd, *J* = 8.2, 2.0 Hz, 1H; H-6′), 6.76 (d, *J* = 8.1 Hz, 1H; H-5′), 5.94 (d, *J* = 2.3 Hz, 1H; H-8), 5.91 (d, *J* = 2.3 Hz, 1H; H-6), 4.82 (s, 1H; H-2), 4.18 (ddd, *J* = 4.5, 3.0, 1.4 Hz, 1H; H-3), 2.86 (dd, *J* = 16.7, 4.6 Hz, 1H; H-4b), 2.74 (dd, *J* = 16.8, 2.9 Hz, 1H; H-4a).
3-β-d-Glucopyranosyloxy-4-methyl-2(5H)-furanone (**15**), white amorphous powder. UV (MeCN, H_2_O) λ_max_ 224. ESITOFMS *m/z* 277.0969 [M + H]^+^ (calcd for C_11_H_16_O_8_, 277.0918). ^1^H NMR (deuterium oxide) *δ*: 5.08 (d, *J* = 7.8 Hz, 1H; H-1′), 4.84 (s, 2H; H-5), 3.91 (dd, *J* = 12.5, 2.0 Hz, 1H; H-6′b), 3.75 (dd, *J* = 12.5, 5.2 Hz, 1H; H-6′a), 3.56 (br d, *J* = 8.7 Hz, 1H; H-3′), 3.54–3.46 (m, 3H: 3.51 H-2′; 3.50 H-5′; 3.49 H-4′), 2.09 (s, 3H; 4-CH_3_). ^13^C NMR (deuterium oxide) δ 172.20 (C-2), 144.42 (C-4), 136.95 (C-3), 101.50 (C-1′), 76.39 (C-5′), 75.47 (C-3′), 73.13 (C-2′), 71.36 (C-5), 69.35 (C-4′), 60.54 (C-6′), 9.98 (4-CH_3_).
Scopolin (**16**), yellow amorphous powder. UV (MeCN, H_2_O) λ_max_ 204, 226, 289, 339. ESITOFMS *m/z* 355.1065 [M + H]^+^ (calcd for C_16_H_19_O_9_, 355.1024). ^1^H NMR (deuterium oxide) *δ*: 8.00 (d, *J* = 9.5 Hz, 1H; H-4), 7.31 (s, 1H; H-5), 7.25 (s, 1H; H-8), 6.44 (d, *J* = 9.5 Hz, 1H; H-3), 5.27 (d, *J* = 7.2 Hz, 1H; H-1′), 3.97 (m, 1H; H-6′a), 3.95 (s, 3H; 6-OCH_3_), 3.79 (m, 3H; H-6′b), 3.74–3.64 (m, 3H: 3.68 H-2′; 3.71 H-3′; 3.66 H-5′), 3.57 (d, *J* = 9.1 Hz, 1H; H-4′). ^13^C NMR (deuterium oxide) *δ*: 165.19 (C-2), 149.28 (C-9), 146.45 (C-6), 146.24 (C-4), 114.33 (C-10), 113.75 (C-3), 110.31 (C-5), 104.02 (C-8), 100.39 (C-1′), 76.72 (C-3′), 75.87 (C-5′), 73.10 (C-2′), 69.63 (C-4′), 60.81 (C-6′), 56.72 (6-OCH_3_).
Isofraxidin-7-*O-*α-l-rhamnopyranosyl-(1”→6′)-β-d-glucopyranoside (**17**), yellow amorphous powder. UV (MeCN, H_2_O) λ_max_ 226, 291, 337. ESITOFMS *m/z* 531.1772 [M + H]^+^ (calcd for C_23_H_31_O_14_, 531.1708). ^1^H and ^13^C NMR see Table 1.
4-Methoxycatechol-2-*O*-(5′′-*O*-vanilloyl-β-apiofuranosyl)-(1′′→2′)-β-glucopyranoside (**18**), white amorphous powder. UV (MeCN, H_2_O) λ_max_ 220, 265, 290. ESITOFMS *m/z* 585.1850 [M + H]^+^ (calcd for C_26_H_33_O_15_, 585.1814). ^1^H and ^13^C NMR see Table 1.
6-(3-Methoxy-4-hydroxyphenyl)-hexane-2,4-diol-2-*O*-hexoside (**19**), UV (MeCN, H_2_O) λ_max_ 225, 280. ESITOFMS *m/z* 403.1993 [M + H]^+^ (calcd for C_19_H_31_O_9_, 403.1963). ^1^H and ^13^C NMR see Table 1.

### 4.8. Caenorhabditis Elegans Culture and Mortality Assay

Cultures of wildtype *C. elegans* (N2 Bristol strain) were maintained as described by Stiernagle [101]. Monoxenic cultures were grown at 20 °C on petri dishes containing Nematode Growth Medium (NGM) supplemented with 800 μL of *Escherichia coli* OP50 strain as a food source [102]. The in vitro assay was performed as previously described [8]. Crude extract, EtOAc and aqueous partition were assayed in concentrations from 0.01 to 5 mg/mL. Screening of fractions was performed at 0.1 and 1 mg/mL and pure compounds were tested at concentrations from 10 to 1000 µM. If necessary, samples were solublized in DMSO up to a maximum final concentration of 1% in the assay. A negative control of test medium containing 1% DMSO was therefore included. Levamisole-hydrochloride (40 mM) served as positive control.

To assess the stability of the tested procyanidin trimers, test solutions were collected after the incubation period, worms were allowed to sink and the supernatants were centrifuged at 10000 rpm 5 min prior to LC-MS analysis.

### 4.9. Statistical Analysis

Statistical analysis of the data obtained from the in vitro assay was performed using GraphPad Prism^®^ Ver. 3 (GraphPad Software, Inc., La Jolla, CA, USA). Mean values of mortality rates were compared by a one-way ANOVA test followed by a Tukey’s Test for multiple comparison. A *p*-value < 0.05 compared to the negative control was considered to be significant. Nonlinear regression (variable slope; top value fixed to 100) was performed to obtain best-fit values for the LC_50_.

## 5. Conclusions

Im summary, the current findings rationalize the traditional use of root extracts from *P. pinnata* as anthelmintic remedies. A-type procyanidins are not only the major class of secondary metabolites, but are also responsible for the anthelmintic activity of the extract. While A-type PACs are not generally superior compared to B-types, the interflavan linkage connecting the flavanol units seems to have a great impact on the bioactivity of the molecule.

Further substances identified, including the three novel compounds **17**, **18** and **19**, mainly belonged to the class of coumarins and phenolic glycosides. Despite its popular medicinal use and frequent phytochemical investigations of the drug, with exception of **15**, none of the isolated compounds has previously been described for *P. pinnata*.

## Figures and Tables

**Table 1:** ^1^H and ^13^C NMR data of compounds **17**, **18** and **19** in D_2_O (**17**, **19**) or CD_3_OD (**18**).

17			18			19		
No.	*δ*_C_ *m*	*δ*_H_*m* (*J*/Hz)	No.	*δ*_C_ *m*	*δ*_H_*m* (*J*/Hz)	No.	*δ*_C_ *m*	*δ*_H_*m* (*J*/Hz)
2	163.86, C		1	142.67, C		1	21.00, CH_3_	1.26, d (6.3)
3	114.88, CH	6.50, d (9.5)	2	152.42, C		2	76.09, CH	4.00, m
4	146.03, CH	7.99, d (9.5)	3	103.02, CH	6.65, d (2.7)	3	43.39, CH_2_	1.64, m
5	105.86, CH	7.14, s	4	149.19, C				1.83, m
6	149.86, C		5	109.38, CH	6.44, dd (8.6, 2.7)	4	68.30, CH	3.77, m
7	141.07, C		6	115.99, CH	6.53, d (8.6)	5	38.67, CH_2_	1.76, m
8	140.40, C		1′	101.82, CH	4.79, d (7.5)			1.83, m
9	142.21, C		2′	78.42, CH	3.63 *	6	30.67, CH_2_	2.62, m
10	116.68, C		3′	78.09, CH	3.35, d (7.8)			2.72, m
1′	102.95, CH	5.14, d (7.8)	4′	78.87, CH	3.59 *	1′	135.32, C	
2′	73.56, CH	3.64 *	5′	71.69, CH	3.35, d (7.8)	2′	113.10, CH	6.97, br s
3′	75.72, CH	3.57 *	6′	62.61, CH_2_	3.66 *	3′	147.60, C	
4′	70.19, CH	3.42 *			3.87 *	4′	142.95, C	
5′	75.31, CH	3.60 *	1′′	110.55, CH	5.50, d (1.3)	5′	115.69, CH	6.88, dd (8.0, 1.3)
6′	68.48, CH_2_	3.73 *	2′′	78.70, CH	4.05, d (1.3)	6′	121.31, CH	6.80, br d (8.0)
		3.92 *	3′′	79.23, C		1′′	101.90, CH	4.37, dd (8.0, 1.2)
1′′	101.37, CH	4.69, d (1.5)	4′′	75.39, CH_2_	3.91, d (9.6)	2′′	73.38, CH	3.20, ddd (9.3, 8.0, 1.2)
2′′	70.21, CH	3.73 *			4.31, d (9.7)	3′′	75.98, CH	3.43, dt (9.1, 1.4)
3′′	70.11, CH	3.51 *	5′′	67.96, CH_2_	4.28, d (11.3)	4′′	69.82, CH	3.36, m
4′′	71.72, CH	3.25 *			4.39, d (11.3)	5′′	76.01, CH	3.39, m
5′′	68.92, CH	3.26 *	1′′′	122.18, C		6′′	60.90, CH_2_	3.70, m
6′′	16.65, CH_3_	1.00, d (5.6)	2′′′	113.73, CH	7.47, d (1.9)			3.90, m
			3′′′	148.71, C		OCH_3_ (3′)	56.09	3.87, d (1.2)
OCH_3_ (8)	62.33	4.05, s	4′′′	152.98, C				
OCH_3_ (6)	56.53	3.92, s	5′′′	115.88, CH	6.78, d (8.3)			
			6′′′	125.27, CH	7.50, dd (8.3, 1.8)			
			COOH (1′′′)	167.75				
			OCH_3_ (6)	56.26	3.74			
			OCH_3_ (3′′′)	56.45	3.87			

* Multiplicity of sugar potons could not be determined due to signal overlap.

## Supplementary Materials

The following are available online. Table S1: ^1^H and ^13^C NMR data of **1** and **2** (CD_3_OD, 280 K). Table S2: ^1^H and ^13^C NMR data of **3a**, **4** and **5** (CD_3_OD, 280 K). Figure S1: MS/MS spectra and proposed fragmentation patterns (modified from [28]) of (**a**) compound **6** and (**b**) compound **1**. Figure S2: +ESI-qTOF chromatograms of (**a**) compound **1**, (**b**) **1** after 72 h of incubation during the anthelmintic assay without addition of ascorbic acid and (**c**) plus 0.01% ascorbic acid. Figure S3 (a)–(f): 1D- and 2D-NMR spectra of compound **15**. Figure S4 (a)–(f): 1D- and 2D-NMR spectra of compound **17**. Figure S5 (a)–(f): 1D- and 2D-NMR spectra of compound **18**. Figure S6 (a)–(d): 1D-and 2D-NMR spectra of compound **19**. Figure S7: ^1^H NMR spectrum of compound **1** (CD_3_OD, 600 MHz, 280 K). Figure S8: ^1^H NMR spectrum of compound **2** (CD_3_OD, 600 MHz, 280 K). Figure S9: ^1^H NMR spectrum of compound **3** (CD_3_OD, 600 MHz, 280 K). Figure S10: ^1^H NMR spectrum of compound **3a** (CDCl_3_, 600 MHz, 299 K). Figure S11: ^1^H NMR spectrum of compound **4** (CD_3_OD, 600 MHz, 280 K). Figure S12: ^1^H NMR spectrum of compound **5** (CD_3_OD, 600 MHz, 280 K). Figure S13: ^1^H NMR spectrum of compound **6** (CD_3_OD, 600 MHz, 280 K). Figure S14: ^1^H NMR spectrum of compound **7** (CDCl_3,_ 600 MHz). Figure S15: ^1^H NMR spectrum of compound **8** (CDCl_3_, 600 MHz). Figure S16: ^1^H NMR spectrum of compound **9** (CD_3_OD, 600 MHz). Figure S17: ^1^H NMR spectrum of compound **10** (acetone-*d*_6_, 600 MHz). Figure S18: ^1^H NMR spectrum of compound **11** (acetone-*d*_6_, 600 MHz). Figure S19: ^1^H NMR spectrum of compound **12** (acetone-*d*_6_, 600 MHz). Figure S20: ^1^H NMR spectrum of compound **13** (acetone-*d*_6_, 600 MHz). Figure S21: ^1^H NMR spectrum of compound **14** (CD_3_OD, 600 MHz). Figure S22: ^1^H NMR spectrum of compound **16** (D_2_O, 600 MHz).
