# Supplementary material for: Anthelmintic A-Type Procyanidins and Further Characterization of the Phenolic Composition of a Root Extract from Paullinia pinnata
Source: Molecules. 2020 May 13;25(10):2287. doi: 10.3390/molecules25102287 (PMC7287971; doi:10.3390/molecules25102287)

Supplementary Materials

Anthelmintic A-Type Procyanidins and Further Characterization of the Phenolic Composition of a Root Extract from *Paullinia pinnata*

Verena Spiegler[1*]

[1] Institute for Pharmaceutical Biology and Phytochemistry, University of Münster, Germany;
E-mail: verena.spiegler@uni-muenster.de

* Correspondence: verena.spiegler@uni-muenster.de

Table S1. ^{1}H and ^{13}C NMR data of **1** and **2** (CD_3OD, 280 K).

Table S2: ^{1}H and ^{13}C NMR data of **3a**, **4** and **5** (CD_3OD, 280 K).

Figure S1: MS/MS spectra and proposed fragmentation patterns (modified from [28]) of (**a**) compound **6** and (**b**) compound **1**.

Figure S2: +ESI-qTOF chromatograms of (**a**) compound **1**, (**b**) **1** after 72 h of incubation during the anthelmintic assay without addition of ascorbic acid and (**c**) plus 0.01 % ascorbic acid.

Figure S3 (a) – (f): 1D- and 2D-NMR spectra of compound **15**.

Figure S4 (a) – (f): 1D- and 2D-NMR spectra of compound **17**.

Figure S5 (a) – (f): 1D- and 2D-NMR spectra of compound **18**.

Figure S6 (a) – (d): 1D- and 2D-NMR spectra of compound **19**.

Figure S7: ^{1}H NMR spectrum of compound **1** (CD_3OD, 600 MHz, 280 K).

Figure S8: ^{1}H NMR spectrum of compound **2** (CD_3OD, 600 MHz, 280 K).

Figure S9: ^{1}H NMR spectrum of compound **3** (CD_3OD, 600 MHz, 280 K).

Figure S10: ^{1}H NMR spectrum of compound **3a** ($CDCl_3$, 600 MHz, 299 K).

Figure S11: ^{1}H NMR spectrum of compound **4** (CD_3OD, 600 MHz, 280 K).

Figure S12: ^{1}H NMR spectrum of compound **5** (CD_3OD, 600 MHz, 280 K).

Figure S13: ^{1}H NMR spectrum of compound **6** (CD_3OD, 600 MHz, 280 K).

Figure S14: ^{1}H NMR spectrum of compound **7** ($CDCl_3$, 600 MHz).

Figure S15: ^{1}H NMR spectrum of compound **8** ($CDCl_3$, 600 MHz).

Figure S16: ^{1}H NMR spectrum of compound **9** (CD_3OD, 600 MHz).

Figure S17: ^{1}H NMR spectrum of compound **10** (acetone-d_6, 600 MHz).

Figure S18: ^{1}H NMR spectrum of compound **11** (acetone-d_6, 600 MHz).

Figure S19: ^{1}H NMR spectrum of compound **12** (acetone-d_6, 600 MHz).

Figure S20: ^{1}H NMR spectrum of compound **13** (acetone-d_6, 600 MHz).

Figure S21: ^{1}H NMR spectrum of compound **14** (CD_3OD, 600 MHz).

Figure S22: ^{1}H NMR spectrum of compound **16** (D_2O, 600 MHz).

Table S1. ^{1}H and ^{13}C NMR data of **1** and **2** (CD_3OD, 280 K).

		**1**		**2**	
Ring	No.	δ_C *m*	δ_H *m* (*J*/Hz)	δ_C *m*	δ_H *m* (*J*/Hz)
Unit I					
C	2	100.0, C		100.1, C	
	3	67.2 , CH	3.27, d (3.5)	67.1, CH	3.31 *
	4	28.8, CH	4.14 d (3.5)	29.0, CH	3.95, d (3.5)
A	5	156.8, C		156.6, C	
	6	98.3, CH	5.96, d (2.4)	97.9, CH	5.84, d (2.4)
	7	157.8, C		158.0, C	
	8	96.6, CH	6.00, d (2.4)	96.5, CH	5.99, d (2.4)
	9	154.2, C		154.0, C	
	10	104.9, C		104.1, C	
B	1′	132.5, C		132.3, C	
	2′	115.8, CH	7.02, d (1.9)	115.7, CH	7.01, d (2.1)
	3′	145.8, C		145.3, C	
	4′	146.6, C		145.6, C	
	5′	115.7, CH	6.76*	115.7, CH	6.80, d (8.3)
	6′	119.9, CH	6.82, dd (8.2, 1.9)	119.8, CH	6.84 dd, (8.3, 2.3)
Unit II					
F	2	78.9, CH	5.70, br s	84.54, CH	4.61, d (9.6)
	3	72.6, CH	4.11 m	73.92, CH	4.56, d (9.5)
	4	38.3, CH	4.56 s	39.08, CH	4.51, d (8.8)
D	5	155.8, C		155.43, C	
	6	96.1, CH	5.79, s	97.2, CH	5.79, s
	7	151.1, C		151.2, C	
	8	106.4, C		106.9, C	
	9	151.8, C		152.3, C	
	10	106.7, C		109.0, C	
E	1′	131.8, C		131.1, C	
	2′	116.7, CH	7.32, d (2.0)	116.5, CH	7.19, d (2.0)
	3′	145.9, C		146.2, C	
	4′	146.3, C		146.7, C	
	5′	116.0, CH	6.84, d (8.2)	116.3, CH	6.89, d (8.1)
	6′	121.4, CH	7.20, dd (8.3, 2.0)	121.2, CH	7.14, dd (8.2, 2.0)
Unit III					
	2	80.3, CH	4.40, s	79.7, CH	4.37, brs
	3	67.5, CH	3.86, m	67.7, CH	4.07, d (4.4)
	4	29.9, CH_2	2.83, m	30.1, CH_2	2.87, dd (17.0, 4.9)<br>2.78, brd (16.9)
	5	156.0, C		156.25, C	
	6	96.4, CH	6.10, s	96.5, CH	6.08, s
	7	155.6, C		156.2, C	
	8	108.9, C		108.6, C	
	9	155.8, C		155.3, C	
	10	100.0, C		100.9, C	
	1′	133.2, C		133.0, C	
	2′	115.5, CH	6.83, d (1.7)	115.3, CH	6.98, d (1.9)
	3′	145.3, C		145.9, C	
	4′	145.5, C		146.7, C	
	5′	115.7, CH	6.82, d (8.2)	116.0, CH	6.83, d (8.2)
	6′	119.4, CH	6.73*	119.3, CH	6.88, dd (8.3, 1.9)

*Multiplicity not determined due to overlapping signals

Table S2: ^{1}H and ^{13}C NMR data of **3a**, **4** and **5** (CD$_3$OD, 280 K).

Ring	No.	**3a** δ_C *m*	**3a** δ_H *m* (*J*/Hz)	**4** δ_C *m*	**4** δ_H *m* (*J*/Hz)
Unit I					
C	2	74.1, CH	5.55, brs	77.0, CH	4.94, s
	3	70.8, CH	5.25, brt (1.5)	73.2, CH	3.86, d (3.8)
	4	34.7, CH	4.68, s	37.3, CH	4.71, brs
A	5	150.0, C[1]		157.9, C	
	6	110.2, CH	6.72, d (2.3)	96.4, CH	5.95, d (2.1)
	7	147.5, C[1]		158.2, C	
	8	107.9, CH	6.77, d (2.3)	96.0, CH	5.98, d (2.2)
	9	154.9, C		155.6, C	
	10	111.1, C		101.8, C	
B	1′	136.0, C		132.4, C	
	2′	123.2, CH	7.53, d (2.2)	115.3, CH	6.81*
	3′	142.0, C		145.5, C	
	4′	143.2, C		145.8, C	
	5′	123.5, CH	7.14, d (8.1)	115.8, CH	6.64, d (8.2)
	6′	124.5, CH	7.24, dd (8.4, 1.9)	119.2, CH	6.53, dd (8.3, 1.0)
Unit II					
F	2	97.3, C		100.3, C	
	3	67.4, CH	5.00, d (3.9)	66.5, CH	3.40, d (3.4)
	4	28.0, CH	4.55, d (3.9)	29.1, CH	4.23, d (3.4)
D	5	148.0, C		155.4, C	
	6	117.5, C		99.5, CH	5.93, s
	7	148.7, C		156.8, C	
	8	109.5, CH	7.01, s	108.3, C	
	9	152.3, C		155.3, C	
	10	113.4, C		105.0, C	
E	1′	135.3, C		132.4, C	
	2′	125.5, CH	7.64, d (2.0)	115.6, CH	7.24, d (1.7)
	3′	141.94, C		145.6, C	
	4′	142.8, C		146.6, C	
	5′	123.2, CH	7.27, d (8.6)	115.9, CH	6.83*
	6′	125.3, CH	7.58, dd (8.6, 2.2)	120.0, CH	6.97, dd (8.3, 1.7)

Ring	No.	**5** δ_C *m*	**5** δ_H *m* (*J*/Hz)
Unit I			
C	2	100.1, C	
	3	66.8, CH	3.28, d (3.6)
	4	28.9, CH	4.24, d (3.6)
A	5	156.7, C	
	6	98.3, CH	5.98, d (2.4)
	7	157.9, C	
	8	96.5, CH	6.06, d (2.3)
	9	154.2, C	
	10	104.9, C	
B	1′	132.3, C	
	2′	115.8, CH	7.15, d (1.9)
	3′	145.5, C	
	4′	146.7, C	
	5′	116.1, CH	6.89, d (8.3)
	6′	119.9, CH	6.91*
Unit II			
F	2	78.7, CH	5.65, s
	3	72.4, CH	4.06, brs
	4	38.4, CH	4.43, s
D	5	154.2, C	
	6	107.6, C	
	7	148.4, C	
	8	106.9, C	
	9	150.3, C	
	10	107.2, C	
E	1′	131.6, C	
	2′	116.7, CH	7.31, d (2.0)
	3′	145.9, C	
	4′	146.3, C	
	5′	115.9, CH	6.83, d (8.2)
	6′	121.4, CH	7.22, dd (8.3, 2.0)

Unit III						Unit III			
I	2	76.3, CH	5.49, d (2.0)	78.9, CH	5.71, s	I	2	80.1, CH	4.10, d (2.3)
	3	70.2, CH	5.21, m	72.6, CH	4.11, brs		3	67.4, CH	3.61, m
	4	33.7, CH	4.58, d (1.9)	38.4, CH	4.56, brs		4	29.7, CH_2	2.78, brs
G	5	150.0, C		155.9, C		G	5	155.5, C	
	6	104.9, CH	6.52, s	95.9, CH	5.74, s		6	96.5, CH	6.08, s
	7	150.4, C		151.0, C			7	156.1, C	
	8	108.8, C		106.3, C			8	108.8, C	
	9	151.7, C		151.8, C			9	155.6, C	
	10	108.1, C		106.7, C			10	99.8, C	
H	1′	134.6, C		131.7, C		H	1′	132.8, C	
	2′	121.9, CH	7.39, d (1.7)	116.7, CH	7.33, d (2.0)		2′	115.4, CH	6.64, d (1.9)
	3′	141.9, C		145.9, C			3′	145.7, C	
	4′	142.3, C		146.3, C			4′	145.4, C	
	5′	123.1, CH	7.11, d (8.3)	116.1, CH	6.84, d (8.3)		5′	115.8, CH	6.71, d (8.1)
	6′	125.7, CH	7.20, dd (8.5, 2.1)	121.4, CH	7.21, dd (8.3, 2.0)		6′	119.2, CH	6.25, dd (8.2, 1.7)
Unit IV						Unit II′			
L	2	76.8, CH	5.15, brs	80.2, CH	4.40, brs		2	76.6, CH	4.74, s
	3	65.5, CH	5.52, dt (4.7, 1.7)	67.6, CH	3.86, brs		3	71.2, CH	4.10, d (2.3)
	4	26.5, CH_2	3.05, dd (18.0, 4.6)	29.9, CH_2	2.83, brs		4	37.6, CH	4.38, s
			2.97, brd (18.0)						
J	5	148.7, C		156.0, C			5	159.4, C	
	6	111.0, CH	6.58, s	96.2, CH	6.10, s		6	96.6, CH	5.88, d (2.3)
	7	147.5, C		155.8, C			7	159.6, C	
	8	118.5, C		108.8, C			8	96.1, CH	5.93, d (2.3)
	9	151.9, C		155.9, C			9	158.0, C	
	10	110.3, C		99.8, C			10	99.2, C	
K	1′	135.6, C		133.1, C			1′	131.6, C	
	2′	121.6, CH	7.25, d (1.9)	115.3, CH	6.81*		2′	116.8, CH	7.11, d (1.3)
	3′	141.6, C		145.4, C			3′	145.9, C	
	4′	142.0, C		145.8, C			4′	146.3, C	
	5′	123.2, CH	7.15, d (8.0)	115.9, CH	6.80*		5′	116.1, CH	6.91*
	6′	123.5, CH	7.13, dd (8.4, 1.9)	119.3, CH	6.75, dd (8.3, 2.4)		6′	120.7, CH	6.92*

[1]Interchangeable. * Multiplicity not determined due to overlapping signals.

(**a**)

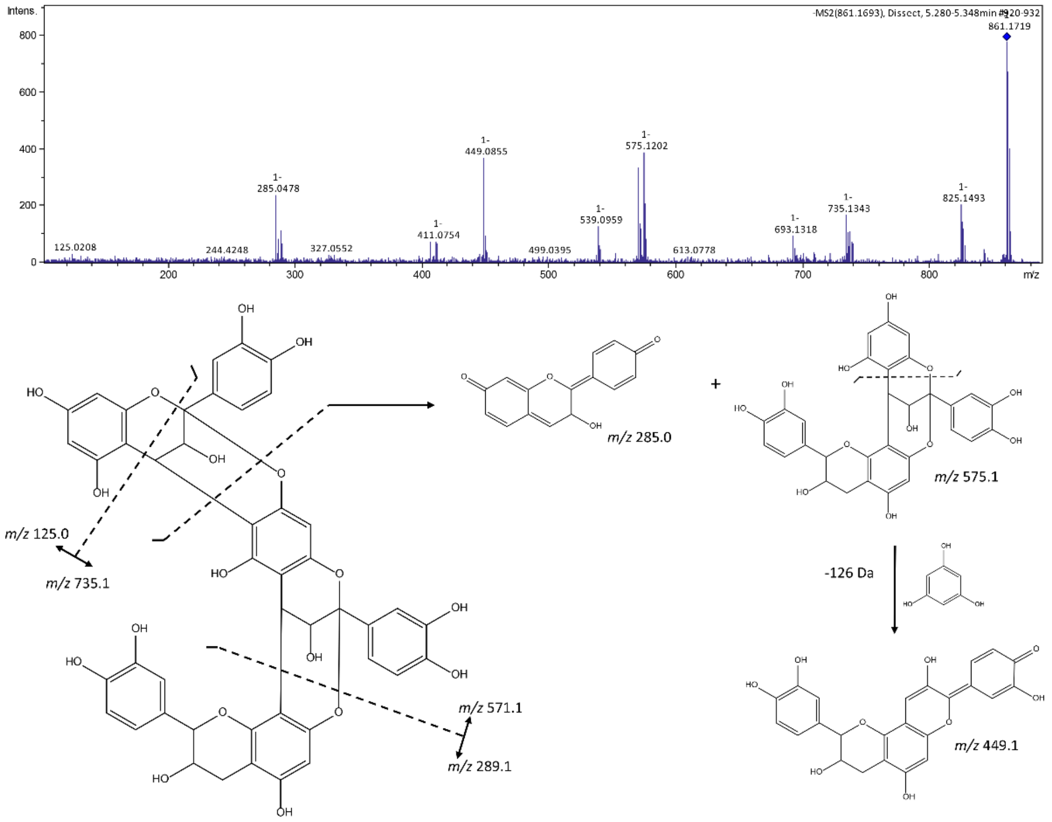

(**b**)

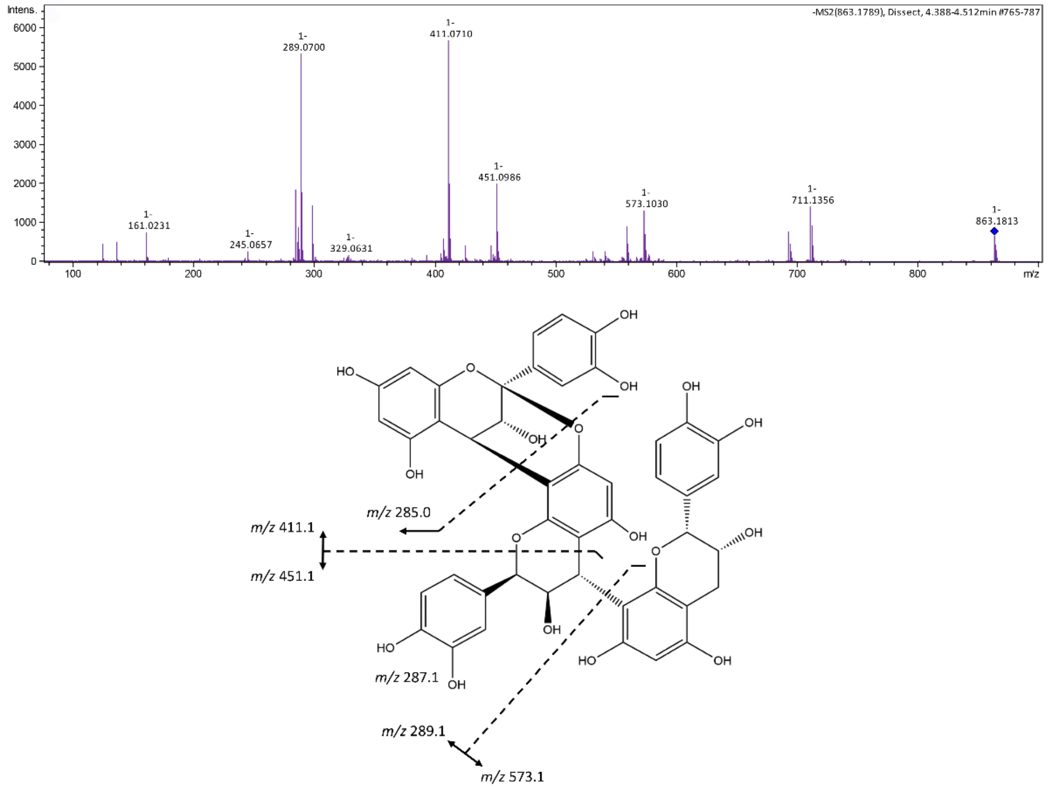

Figure S1: MS/MS spectra and proposed fragmentation patterns (modified from [28]) of (**a**) compound **6** and (**b**) compound **1**, showing characteristic fragments and respective measured *m*/*z* values.

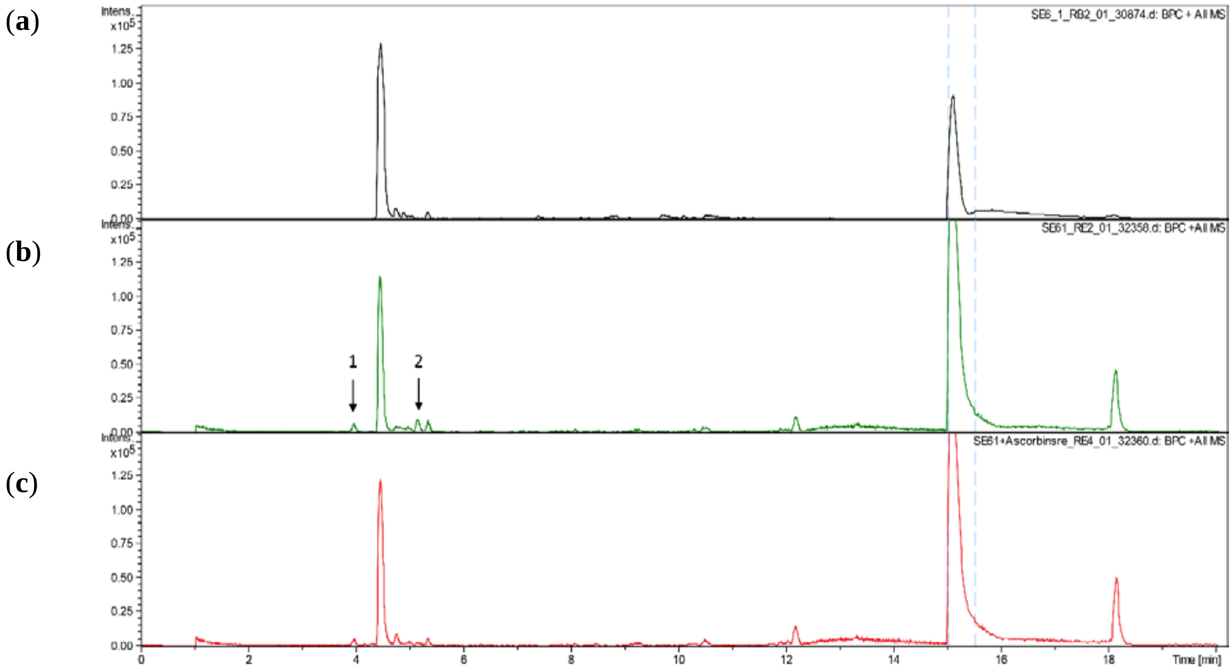

Figure S2: +ESI-qTOF chromatograms of (**a**) compound **1**, (**b**) **1** after 72 h of incubation during the anthelmintic assay without addition of ascorbic acid and (**c**) plus 0.01 % ascorbic acid. Arrows indicate formation of minor side products 1 and 2 during incubation time. Formation of 2 was generally prevented by addition of ascorbic acid.

Figure S3: 1D- and 2D-NMR spectra of compound **15**.

a) ^{1}H NMR spectrum of compound **15** (D_2O, 600 MHz).

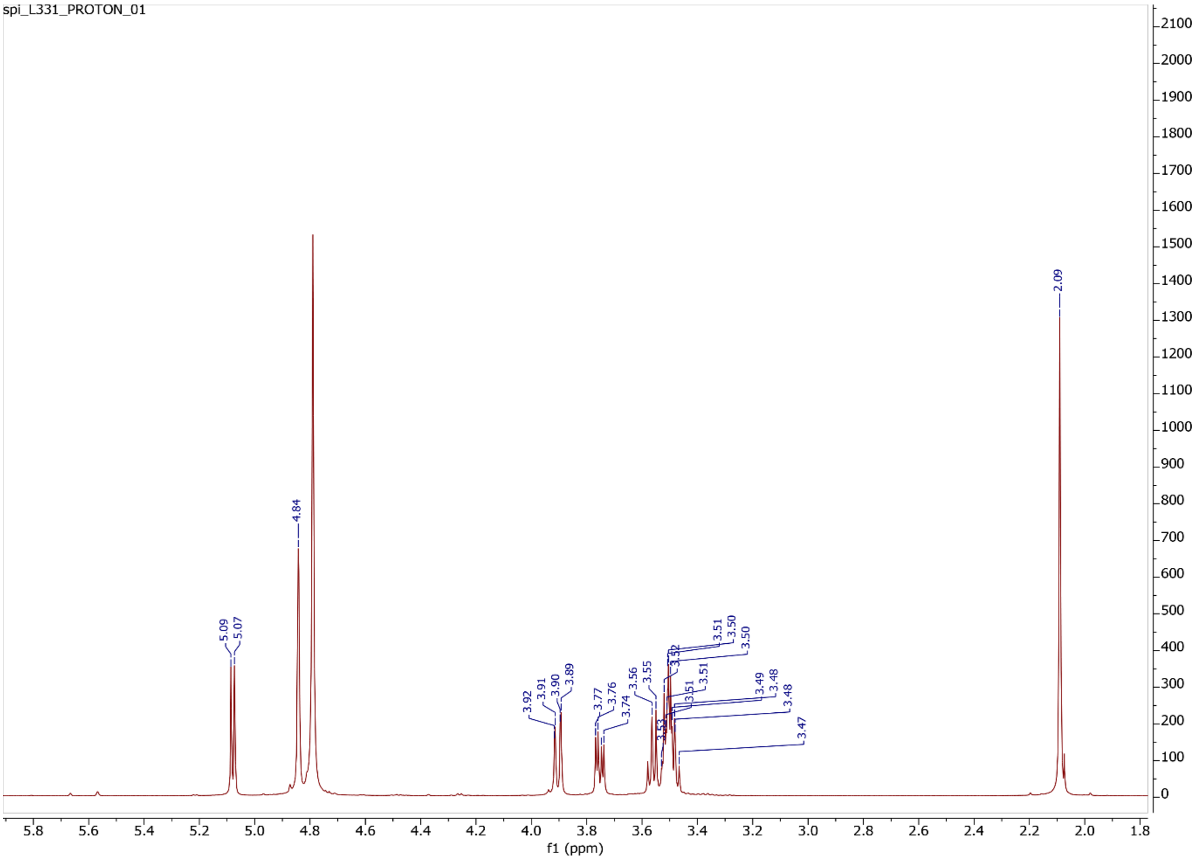

b) ^{13}C NMR spectrum of compound **15** (D_2O, 150 MHz).

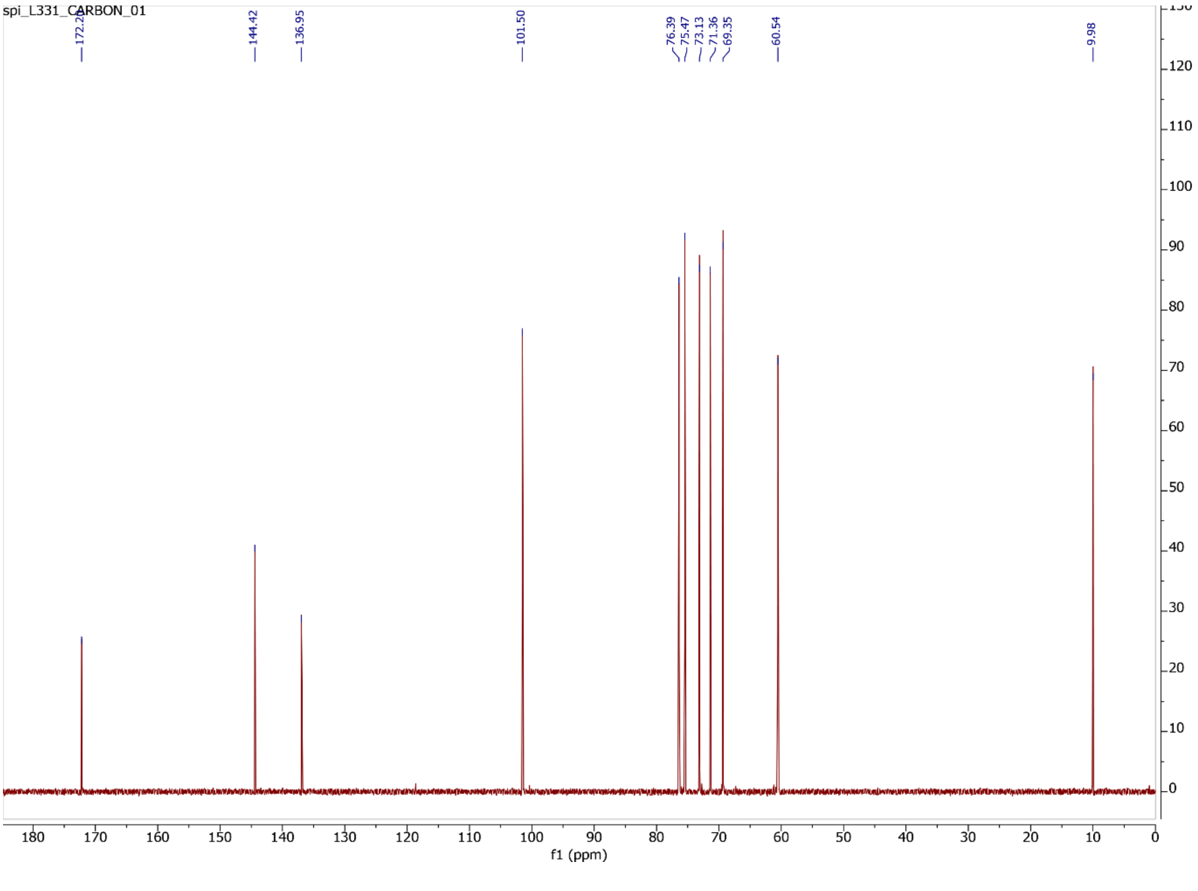

c) COSY spectrum of compound **15** (D_2O, 600 MHz).

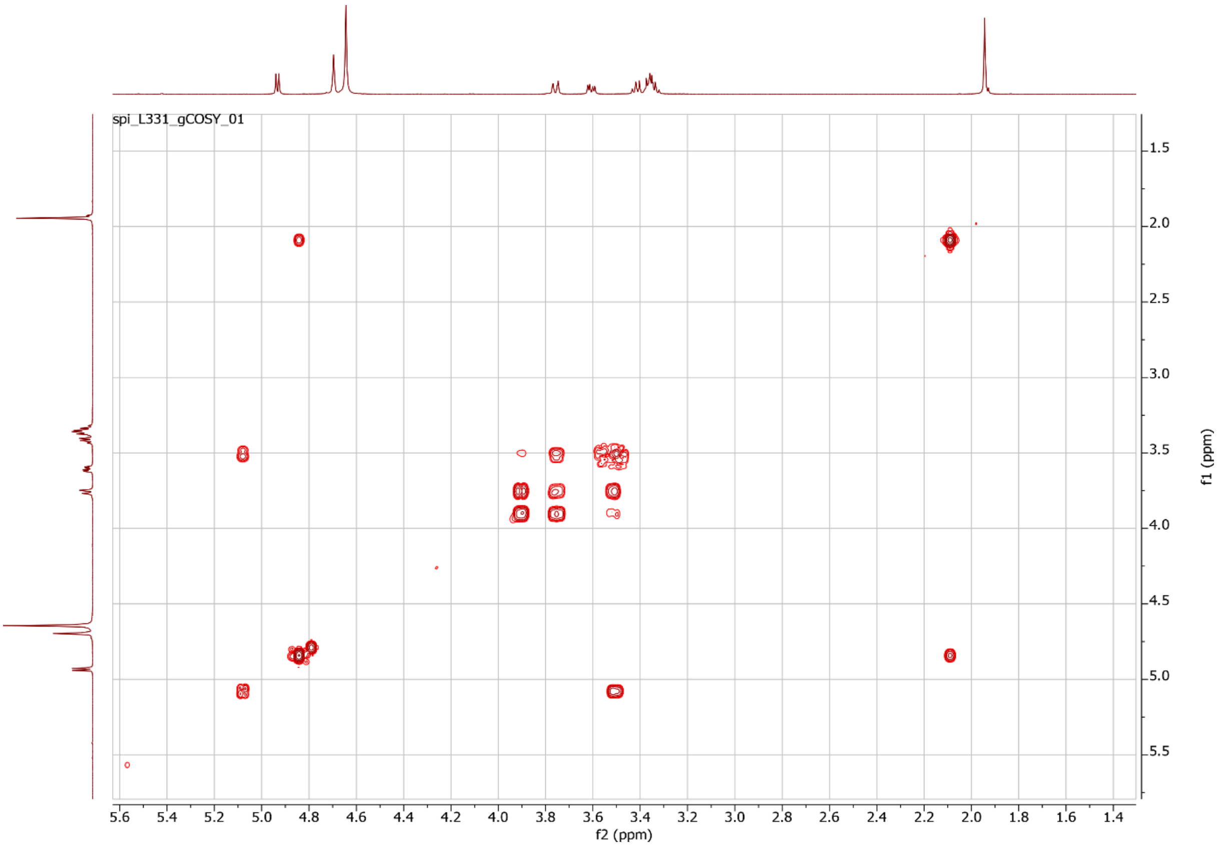

d) HSCQ spectrum of compound **15** (D_2O, 600 MHz).

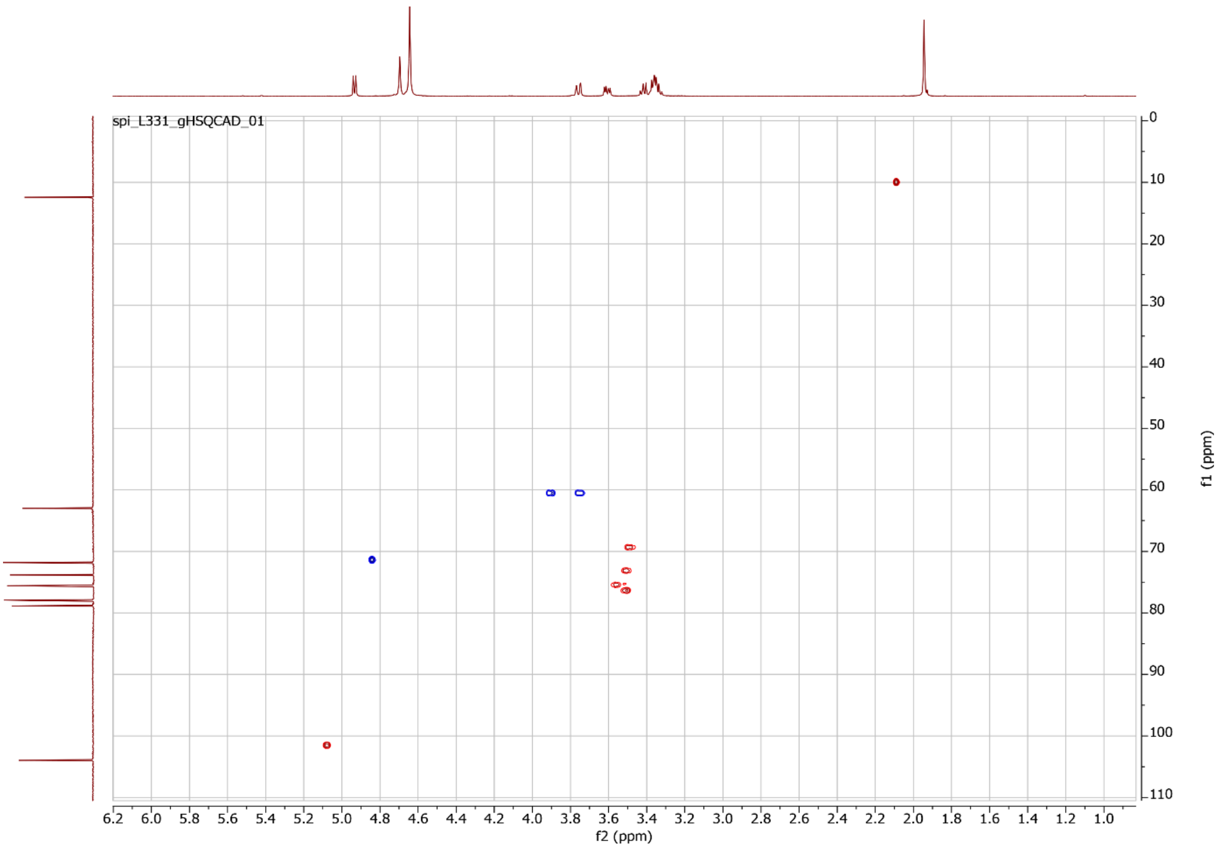

e) HMBC spectrum of compound **15** (D_2O, 600 MHz).

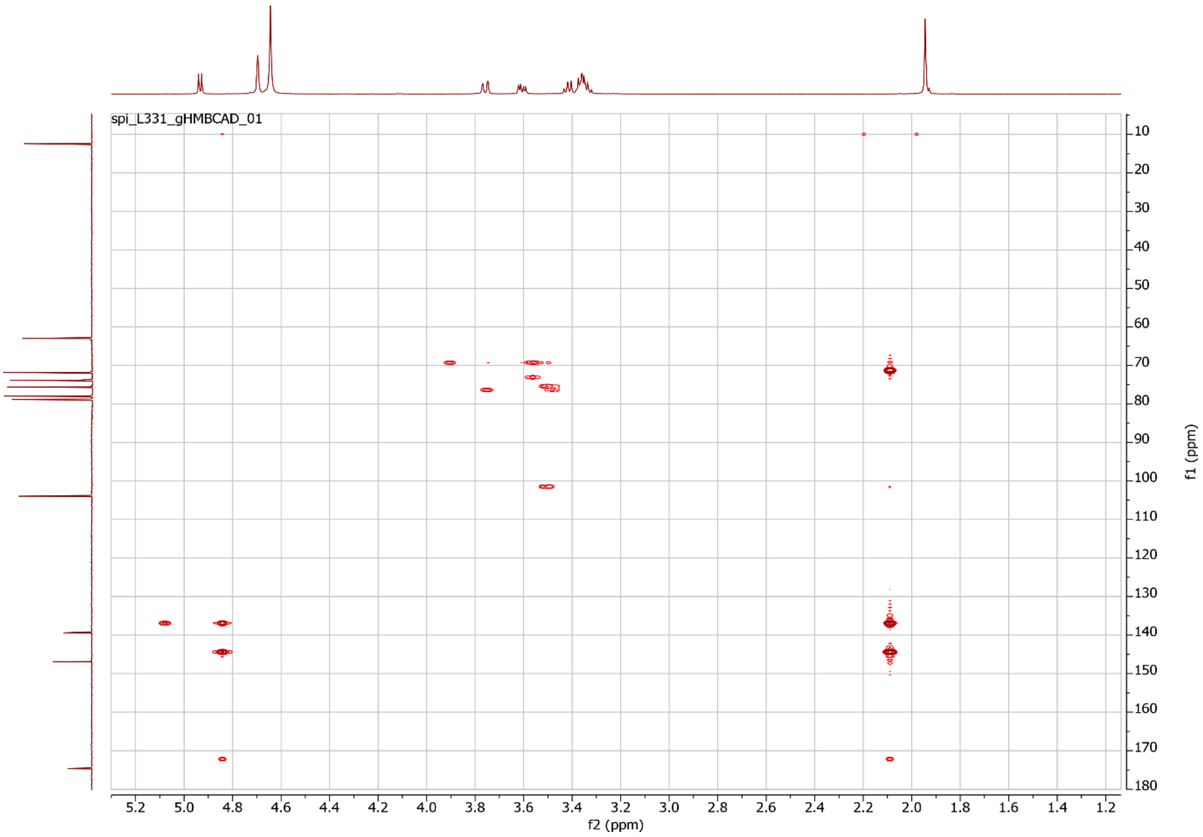

f) NOESY spectrum of compound **15** (D_2O, 600 MHz).

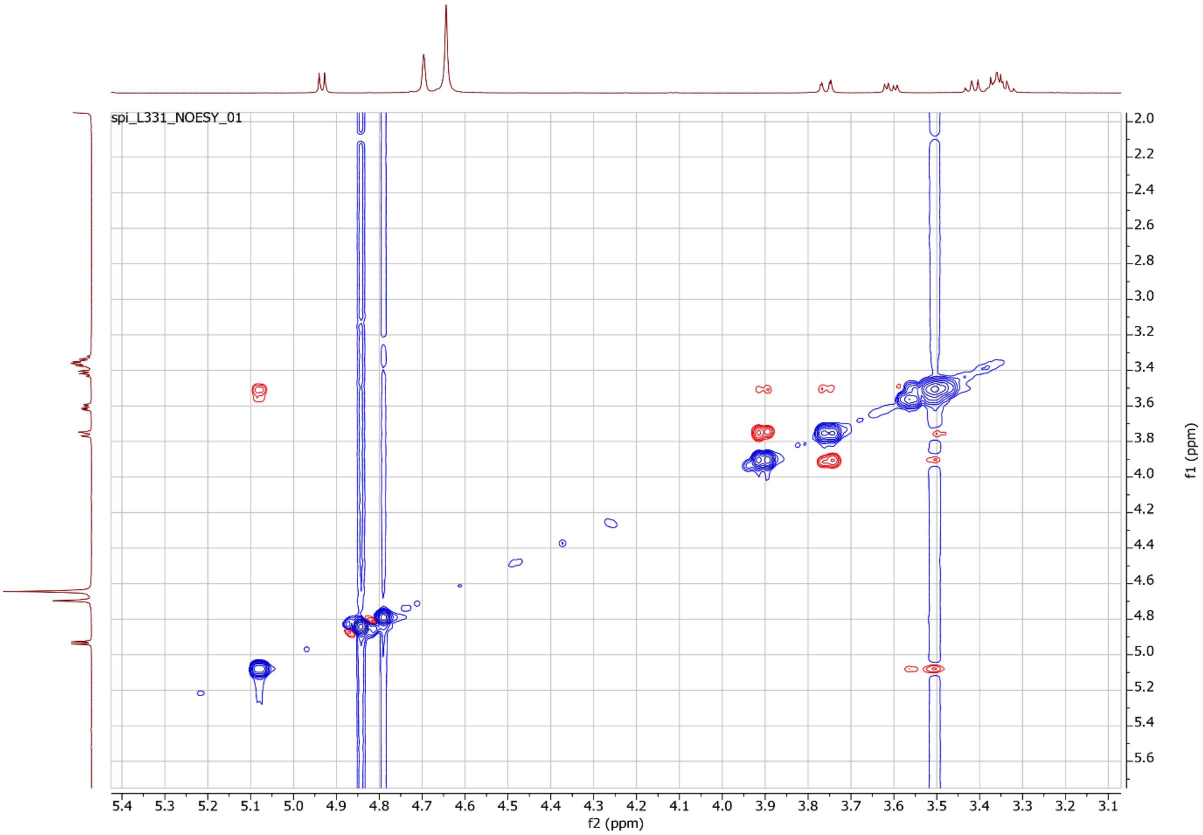

Figure S4: 1D- and 2D-NMR spectra of compound **17**.

a) ^{1}H NMR spectrum of compound **17** (D_2O, 600 MHz).

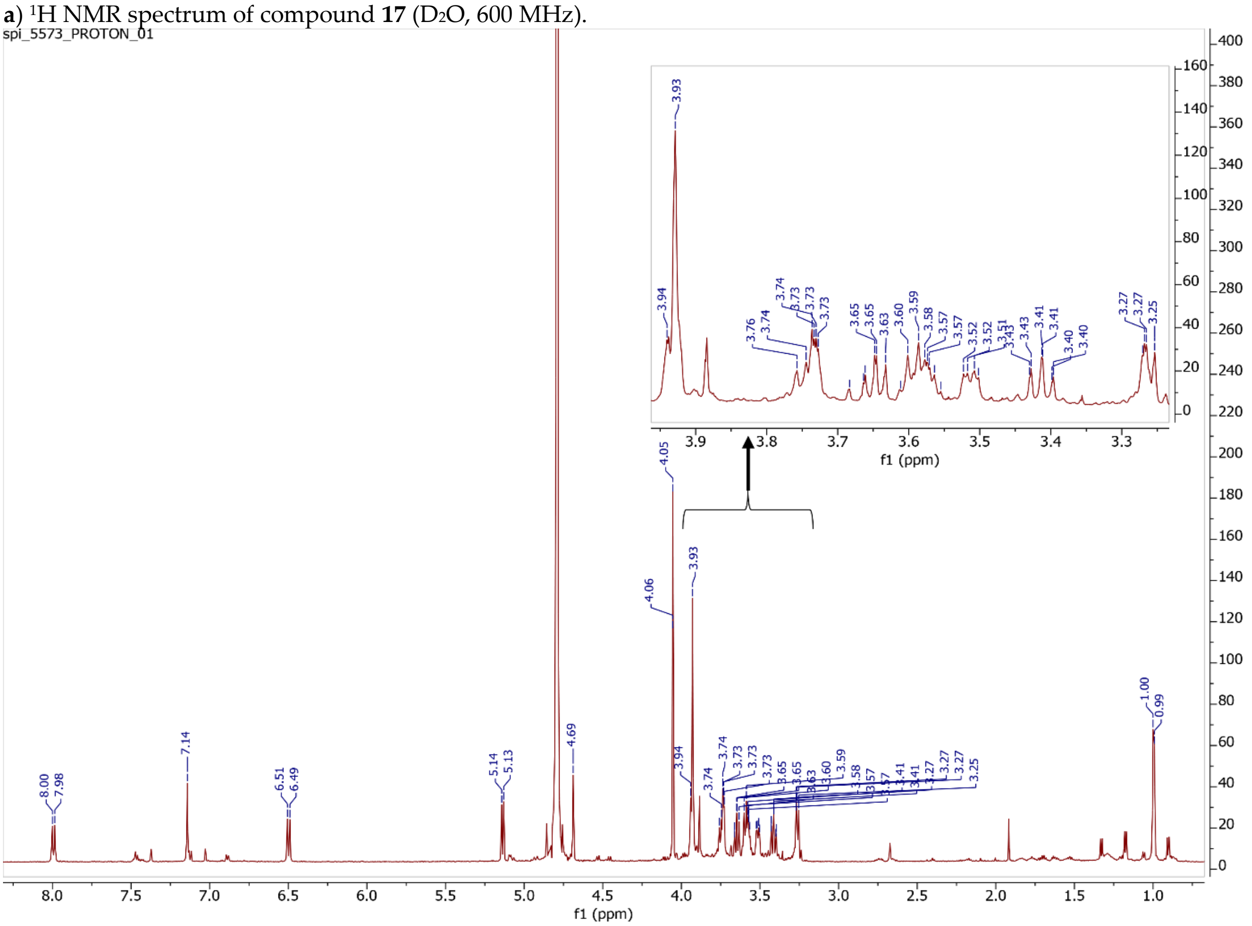

b) HSCQ spectrum of compound **17** (D_2O, 600 MHz).

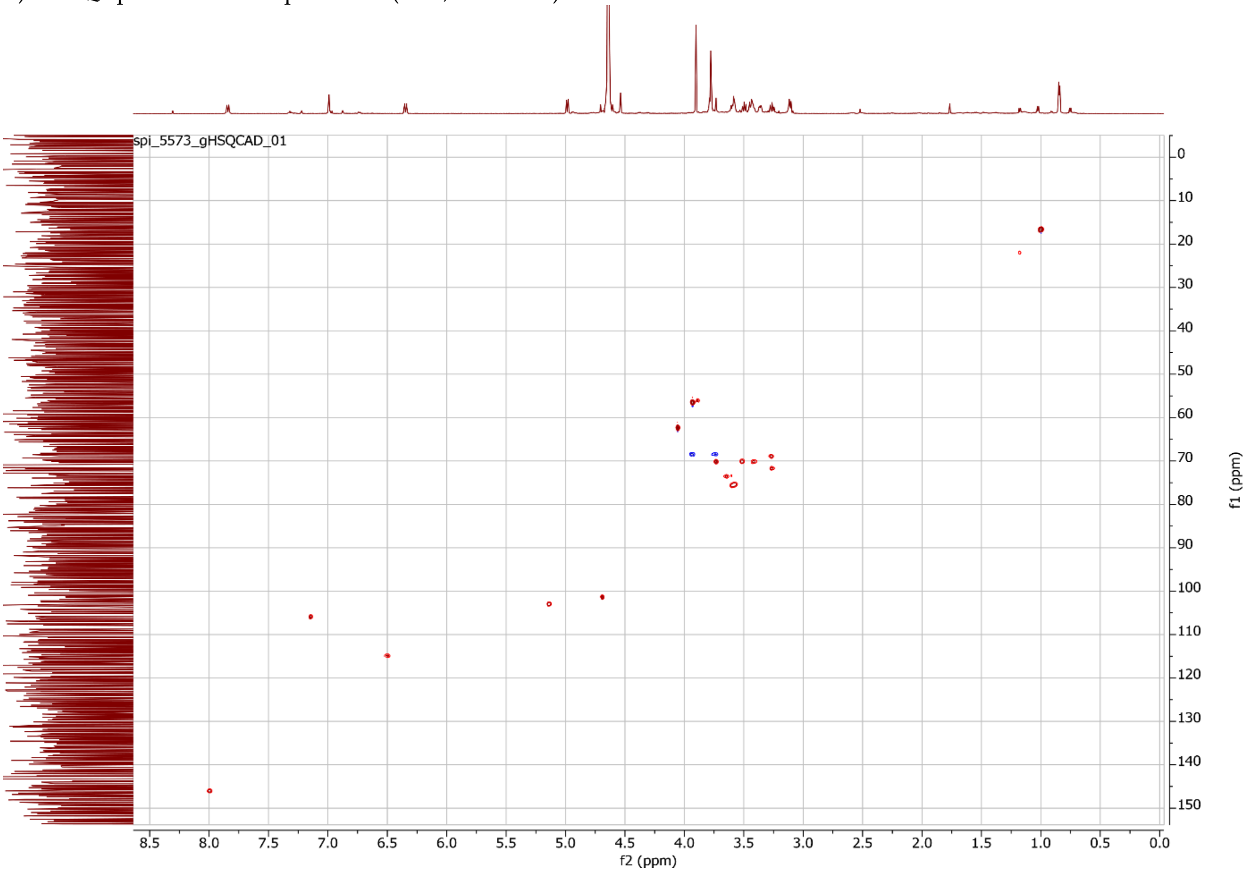

c) HMBC spectrum of compound **17** (D_2O, 600 MHz).

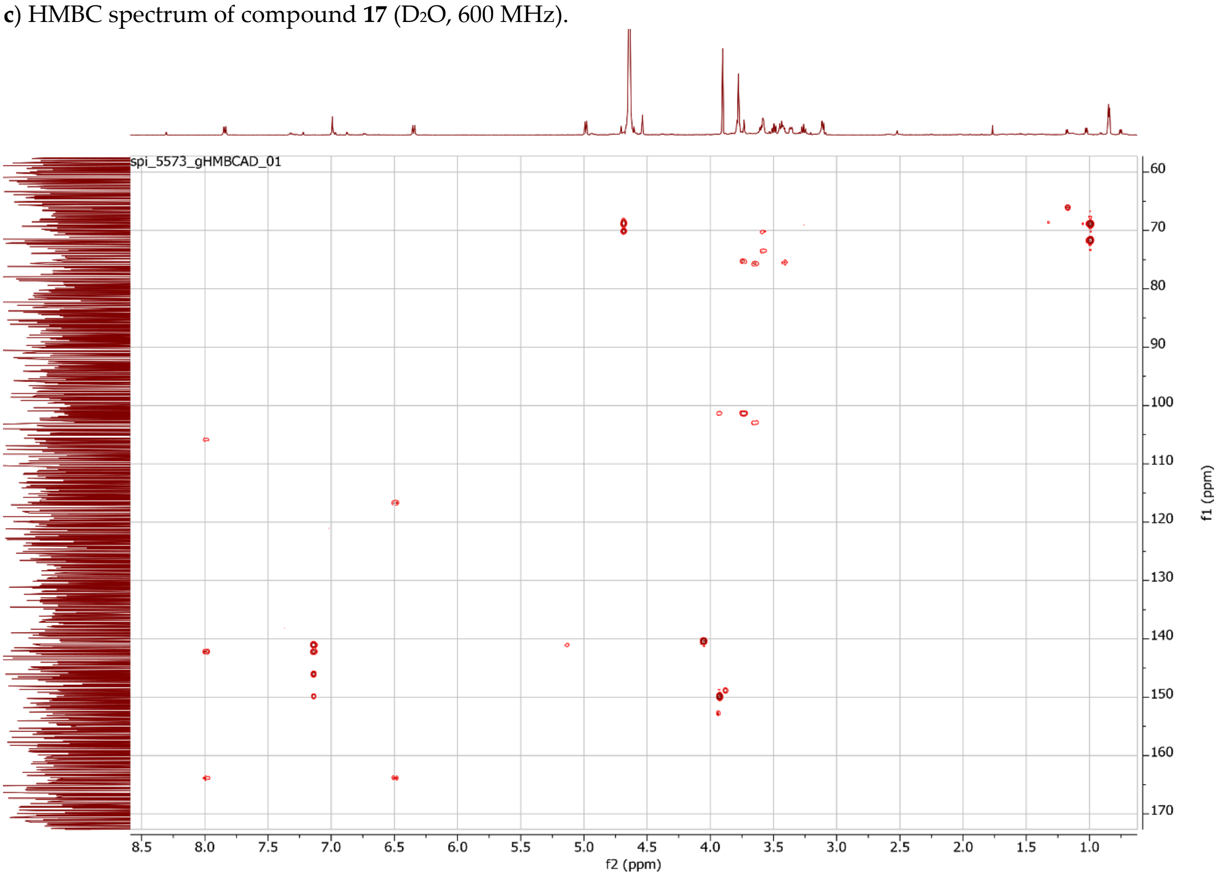

d) COSY spectrum of compound **17** (D_2O, 600 MHz).

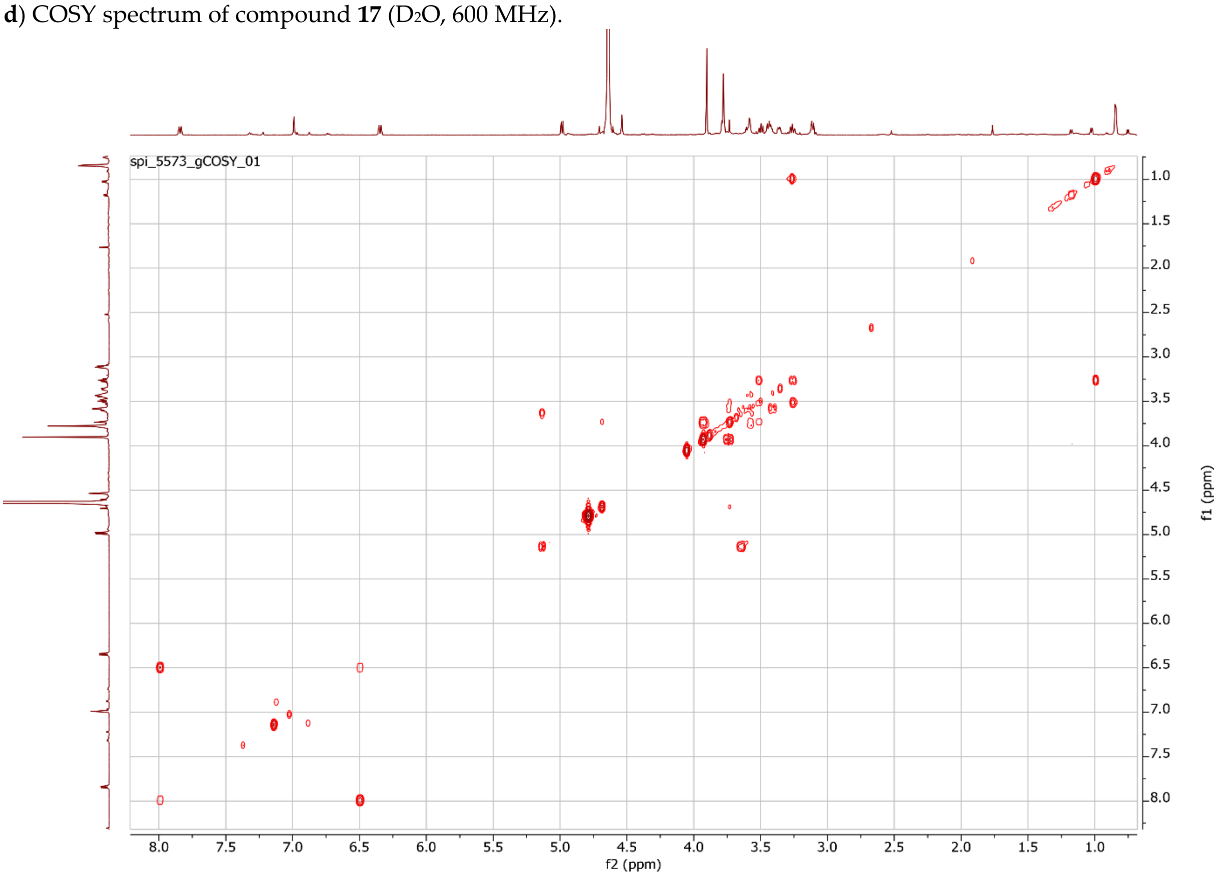

e) TOCSY spectrum of compound **17** (D_2O, 600 MHz).

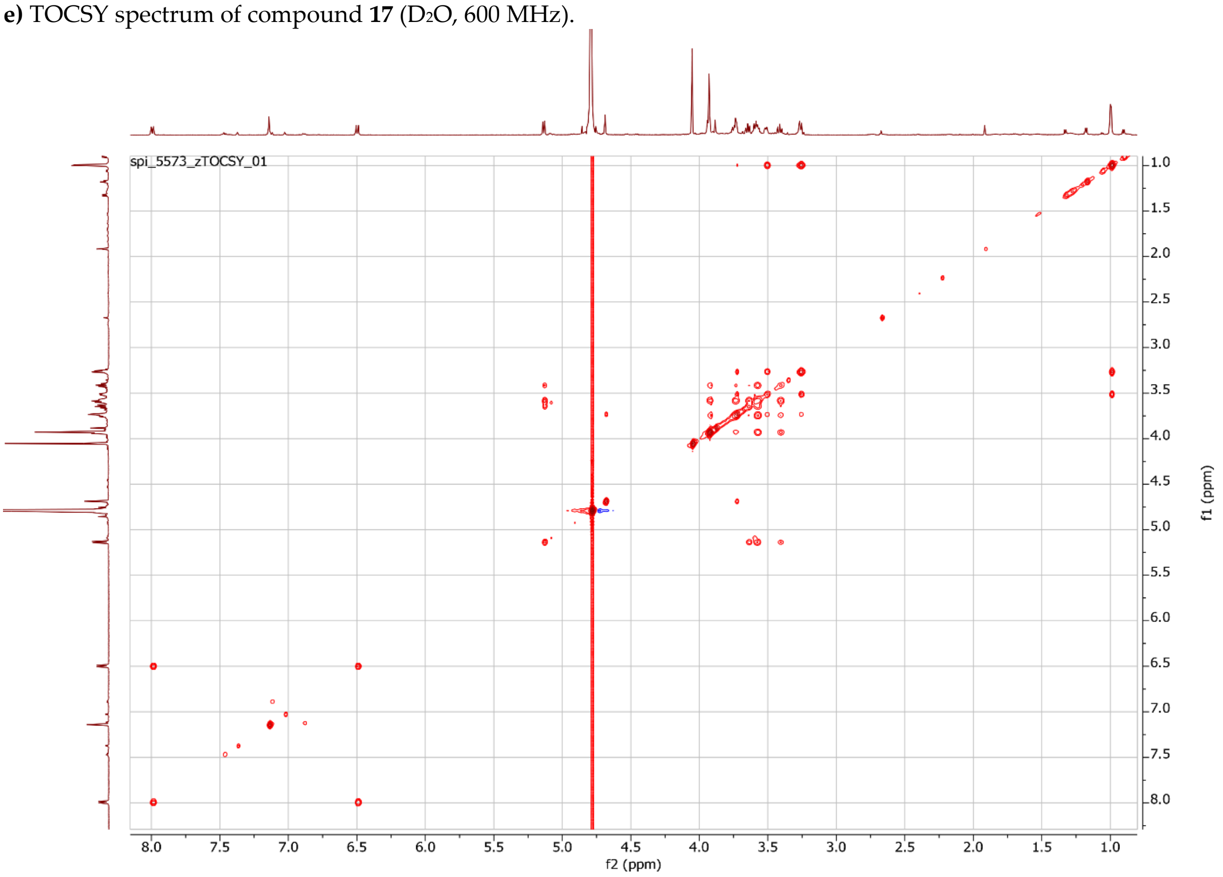

f) NOESY spectrum of compound **17** (D_2O, 600 MHz).

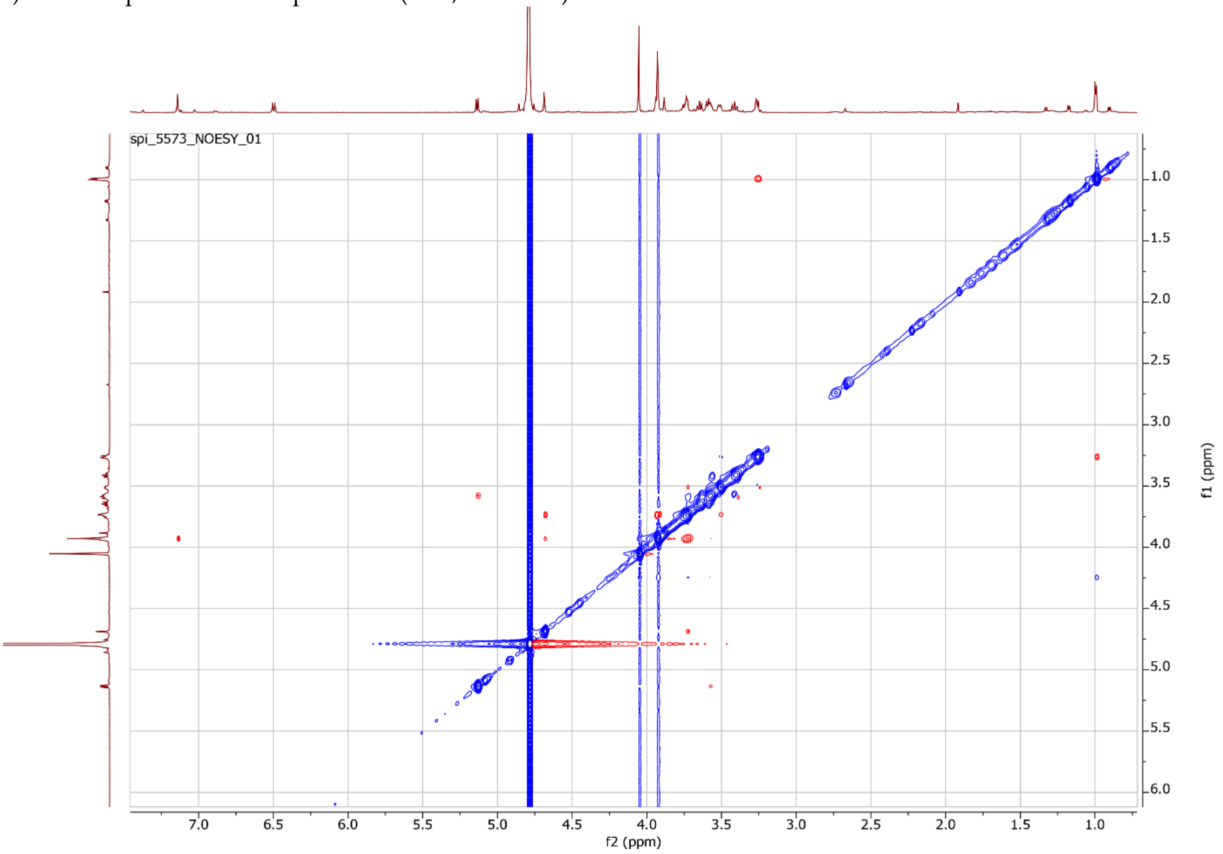

Figure S5: 1D- and 2D-NMR spectra of compound **18**.

a) ^{1}H NMR spectrum of compound **18** (D_2O, 600 MHz).

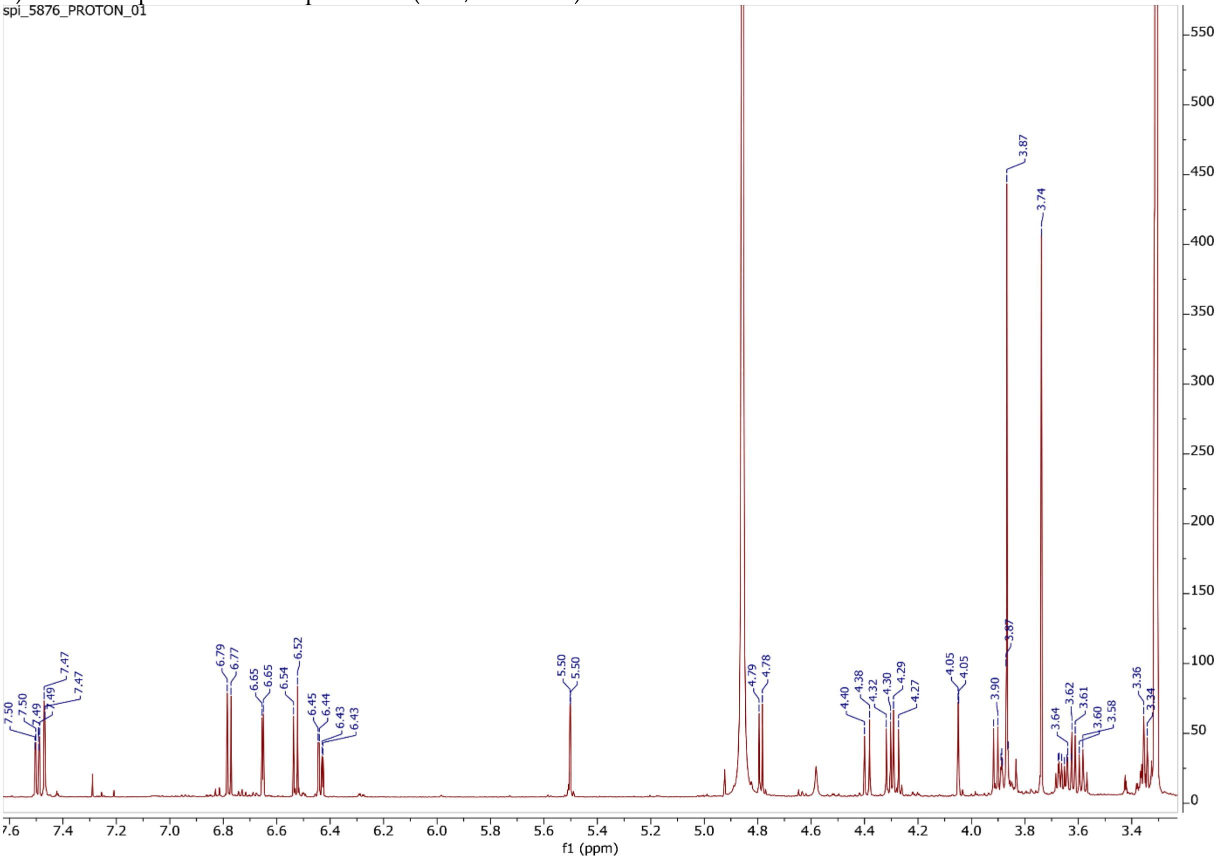

b) HSCQ spectrum of compound **18** (D_2O, 600 MHz).

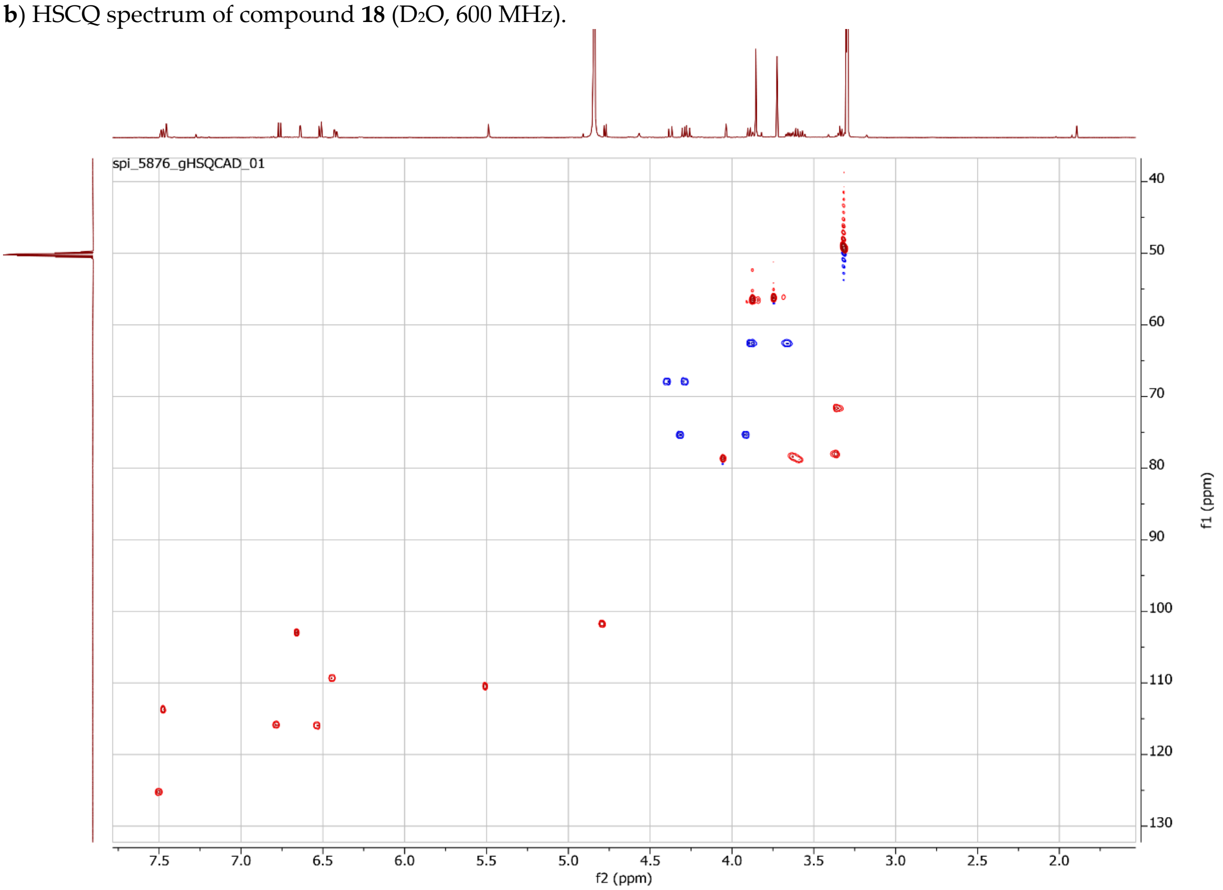

c) HMBC spectrum of compound **18** (D_2O, 600 MHz).

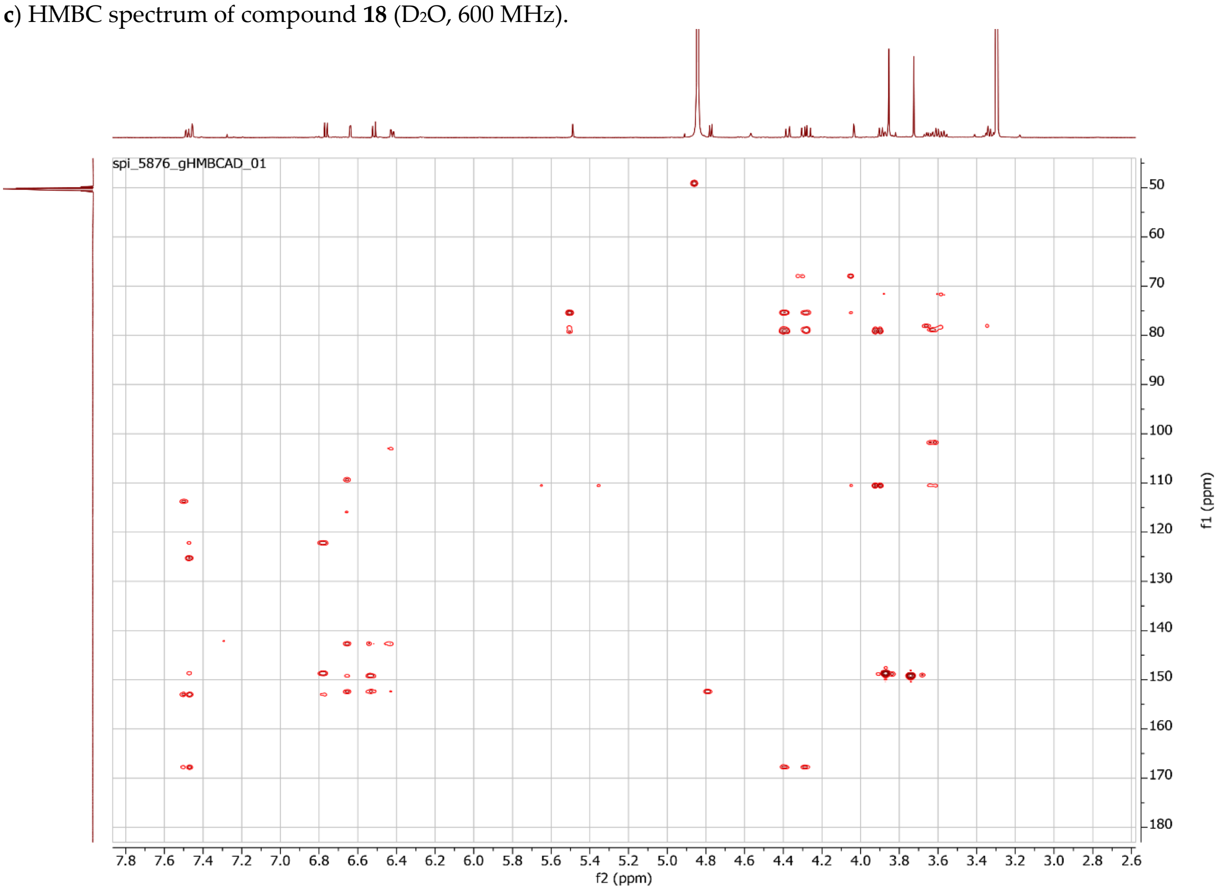

d) COSY spectrum of compound **18** (D_2O, 600 MHz).

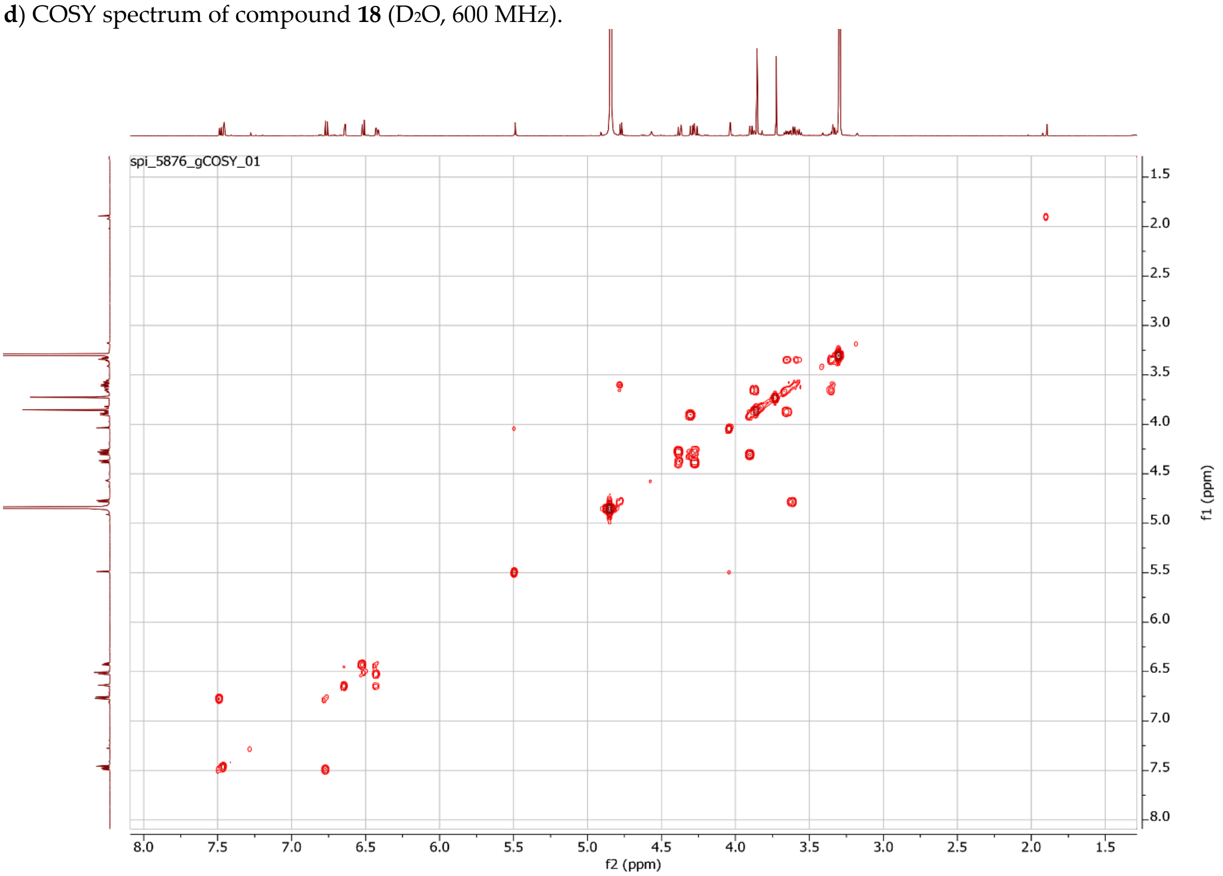

e) TOCSY spectrum of compound **18** (D_2O, 600 MHz).

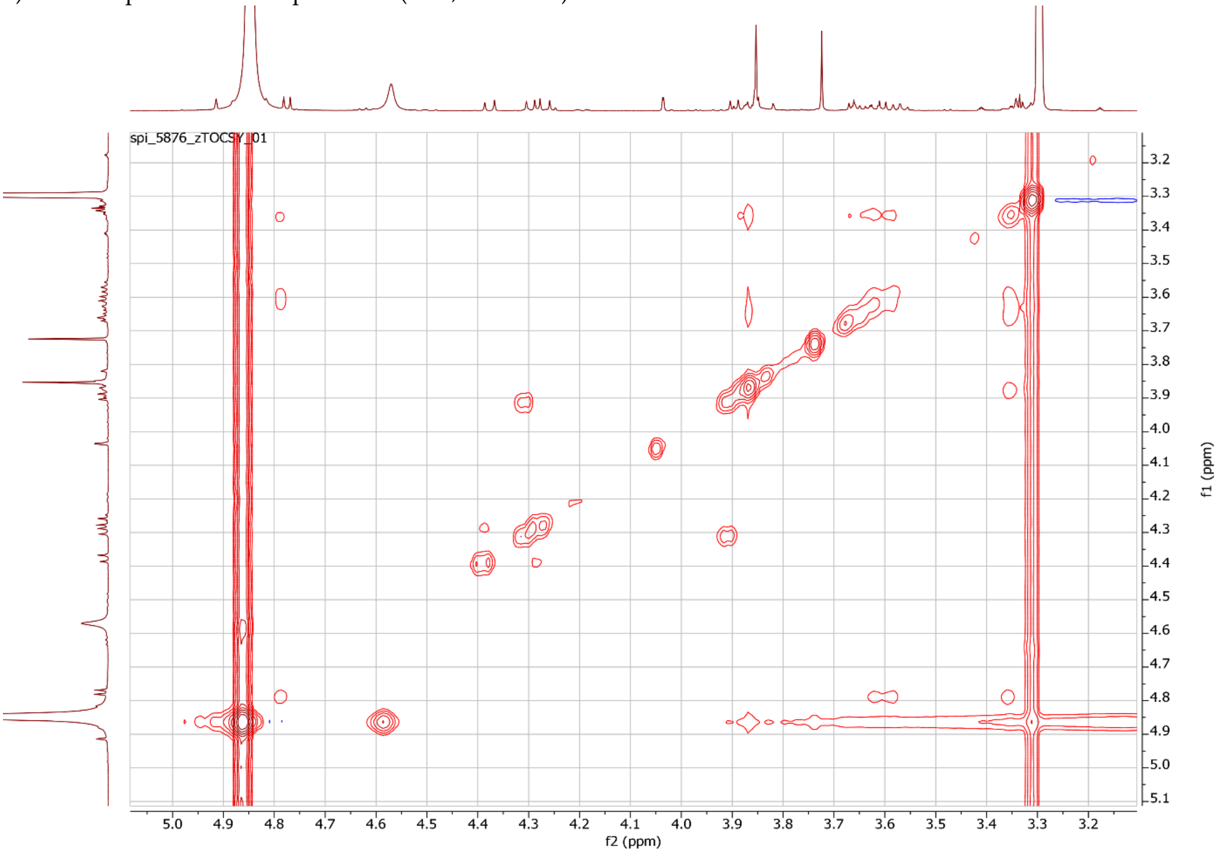

f) H2BCAD spectrum of compound **18** (D_2O, 600 MHz).

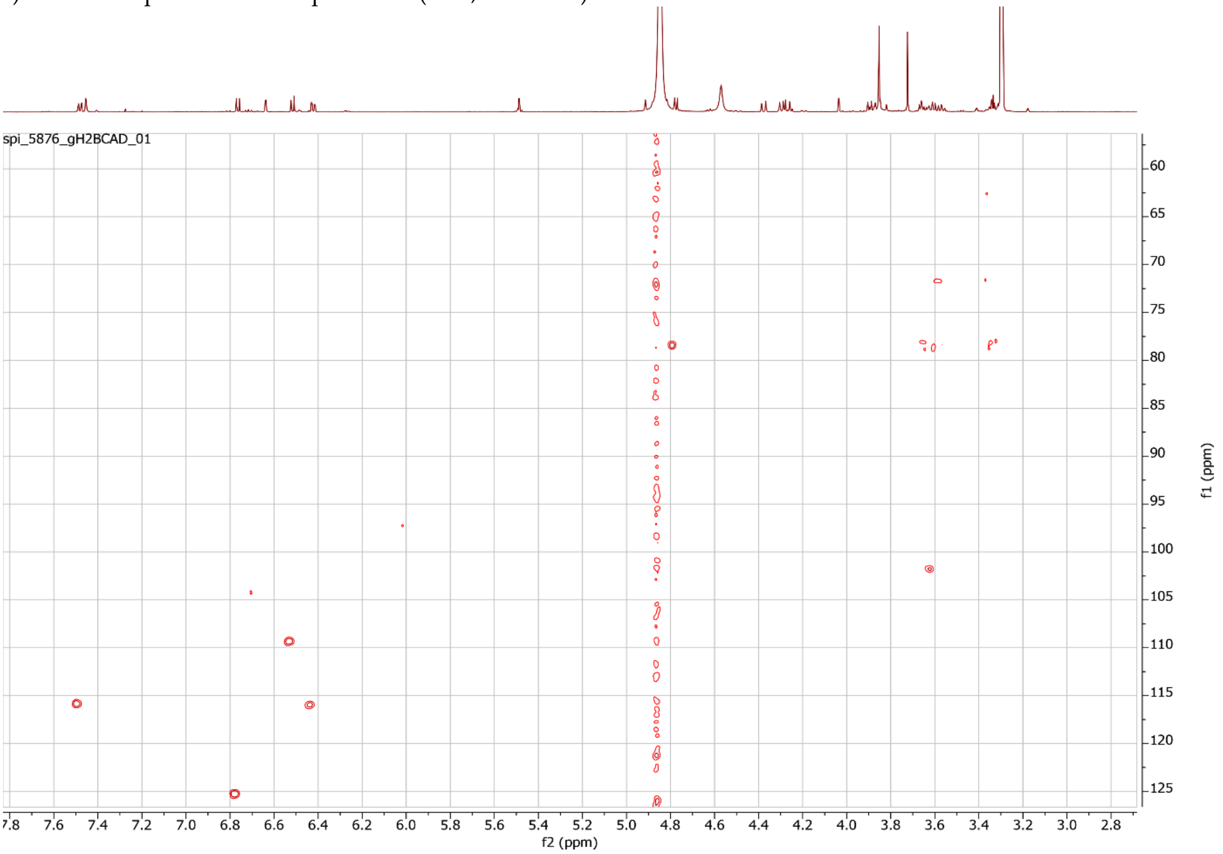

Figure S6: 1D- and 2D-NMR spectra of compound **19**.

a) ^{1}H NMR spectrum of compound **19** (D_2O, 600 MHz).

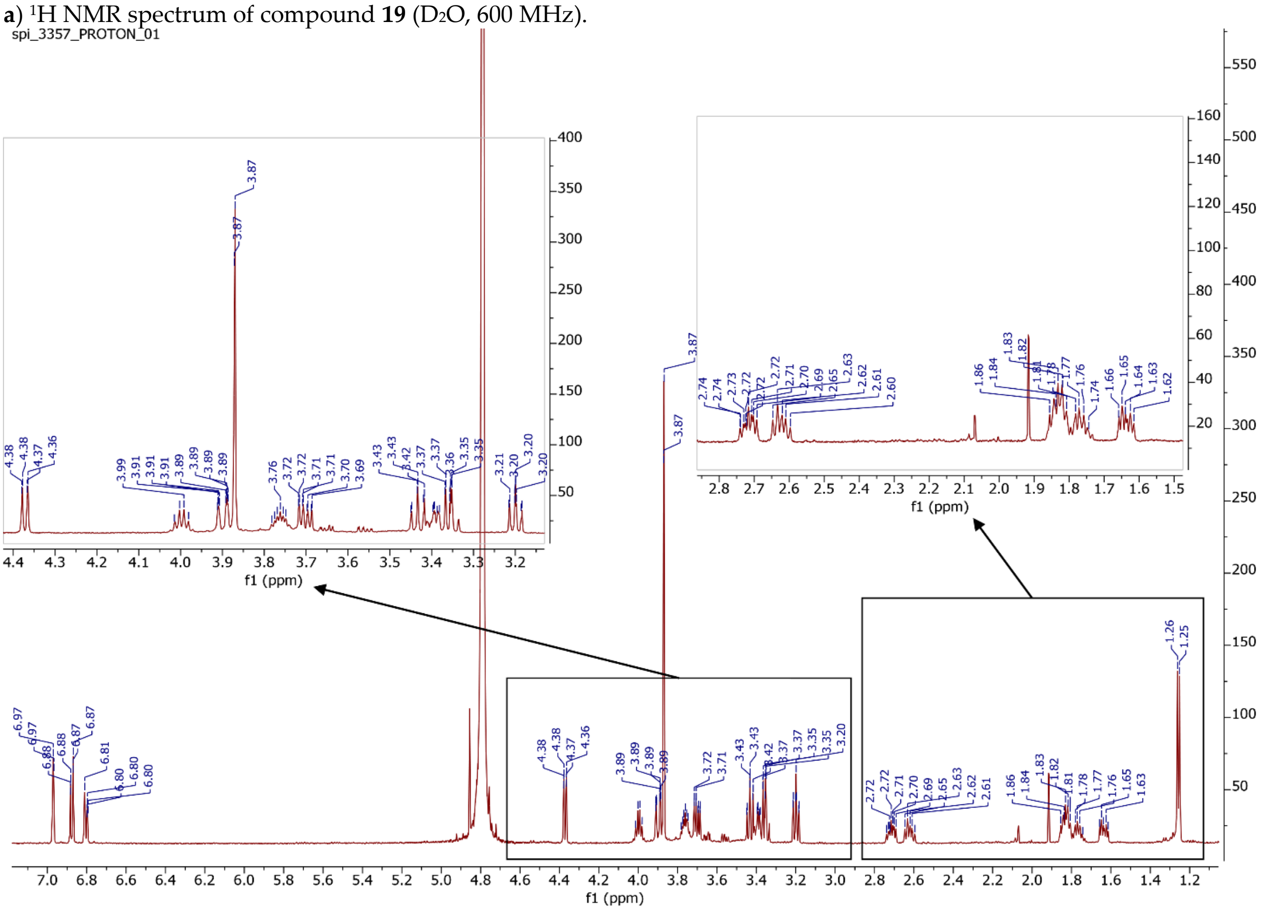

b) HSCQ spectrum of compound **19** (D_2O, 600 MHz).

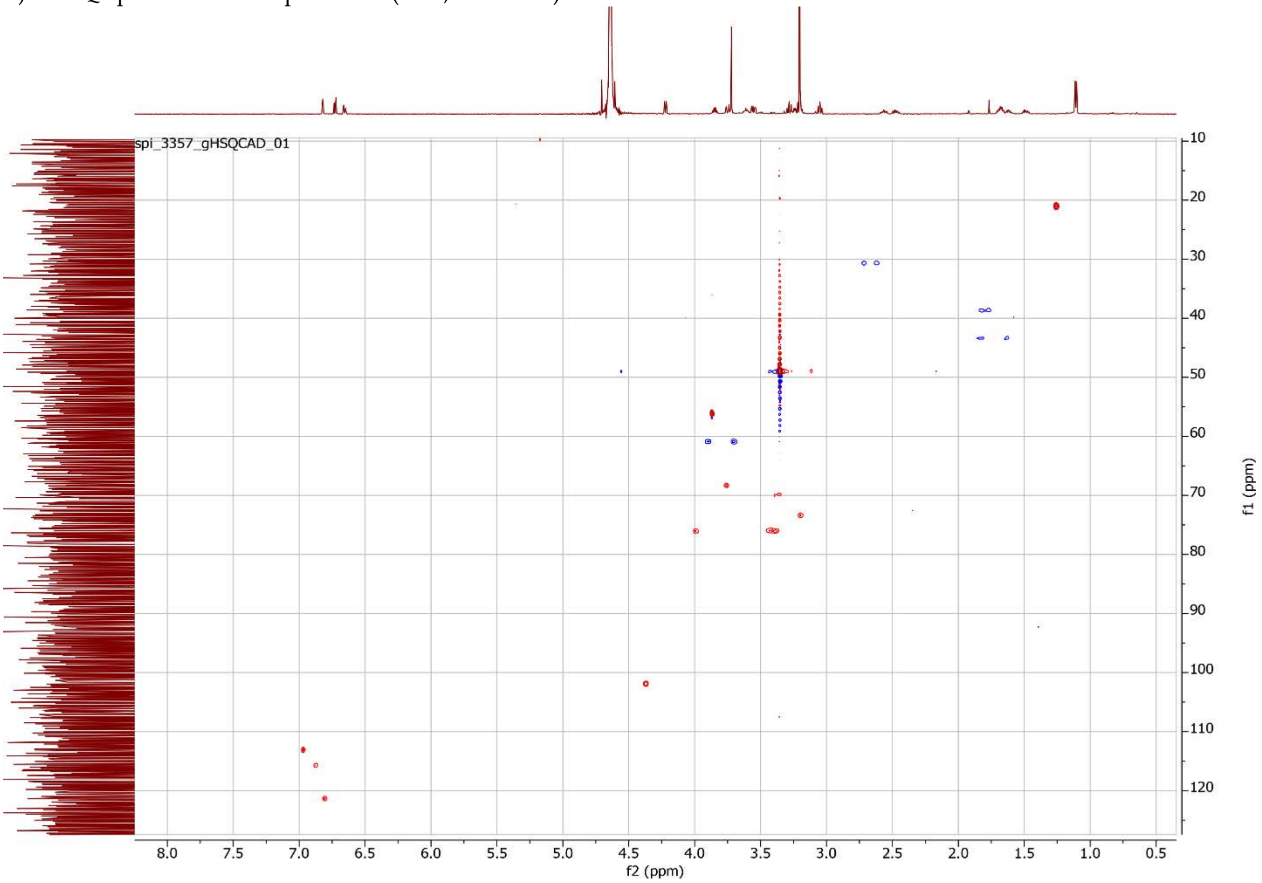

c) HMBC spectrum of compound **19** (D_2O, 600 MHz).

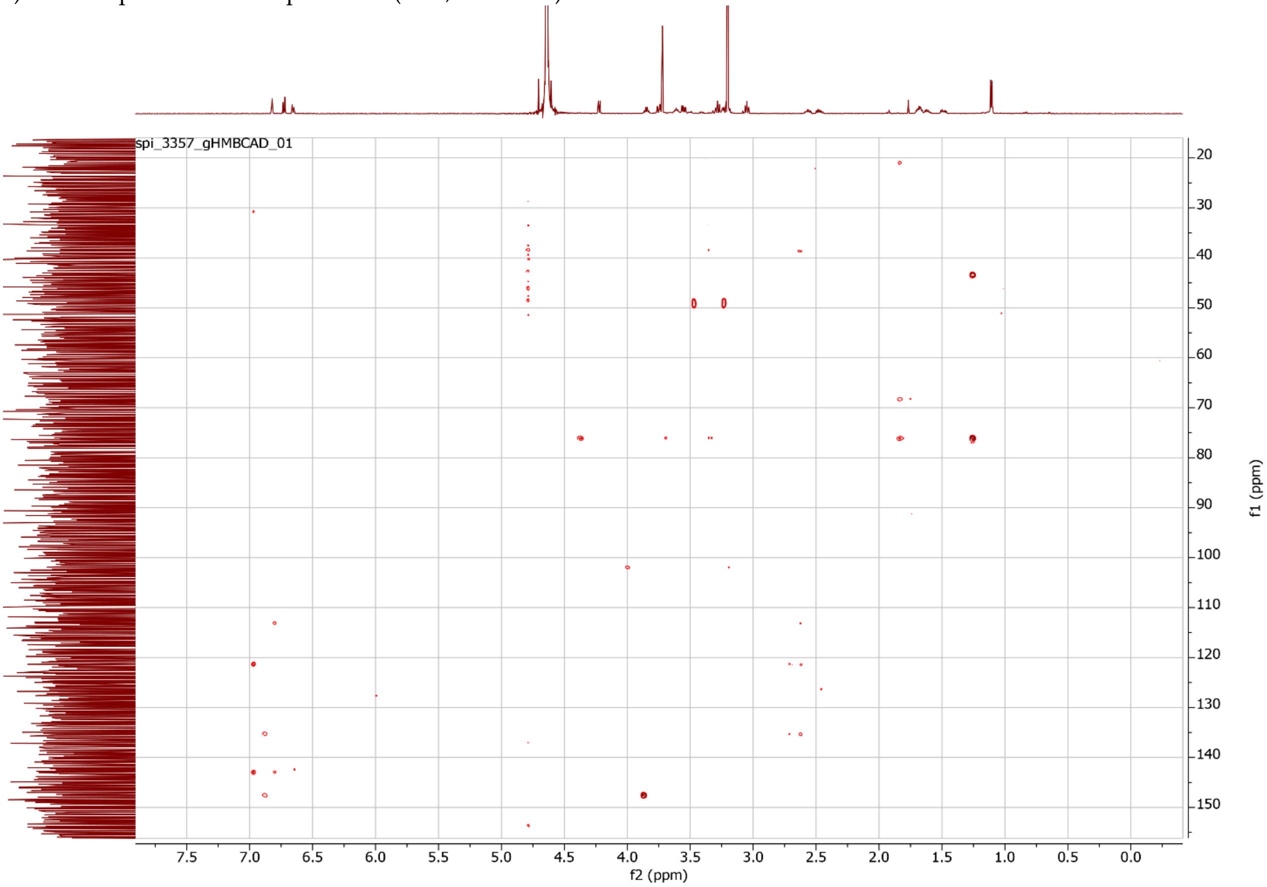

d) COSY spectrum of compound **19** (D_2O, 600 MHz).

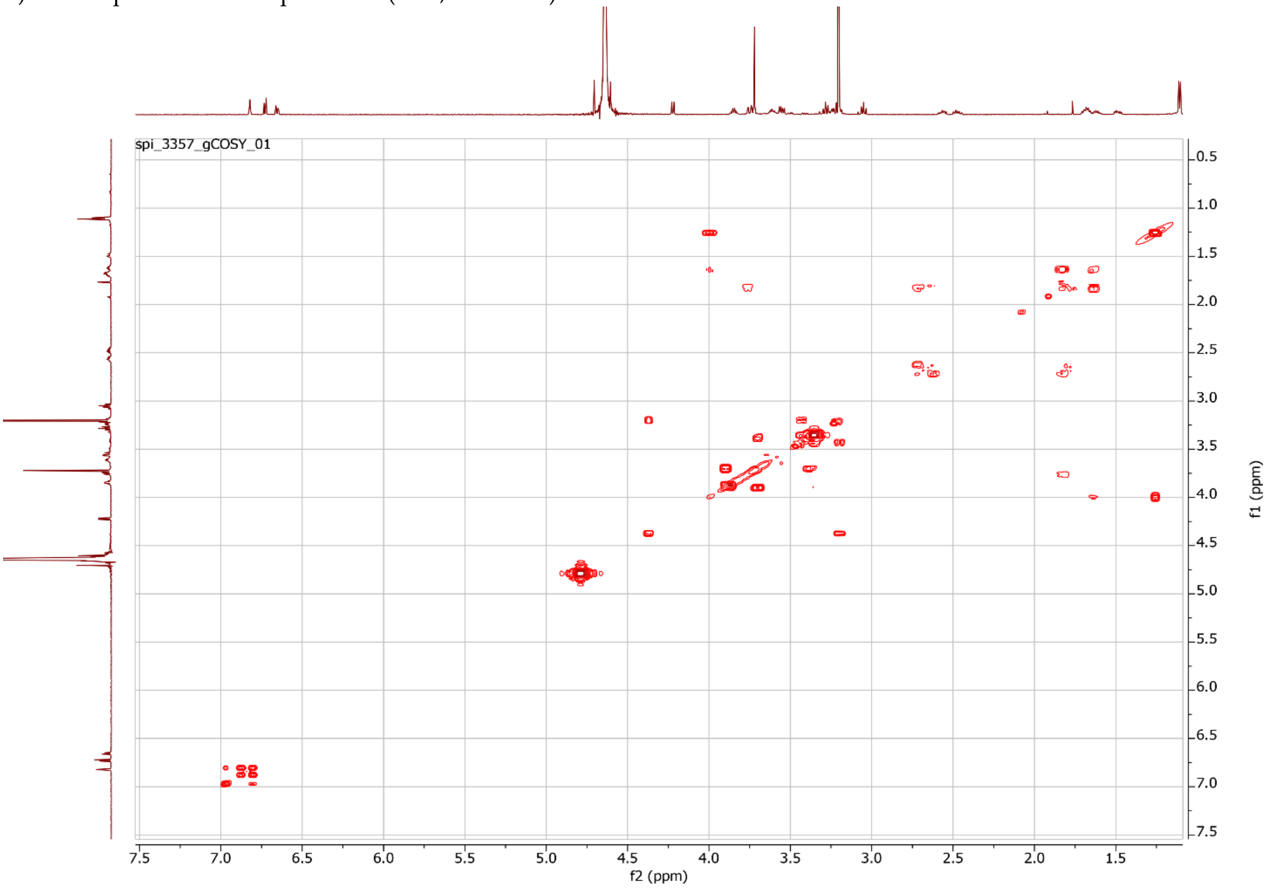

Figure S7: ^{1}H NMR spectrum of compound **1** (CD_3OD, 600 MHz, 280 K). Peak assignment was performed for major rotamers only.

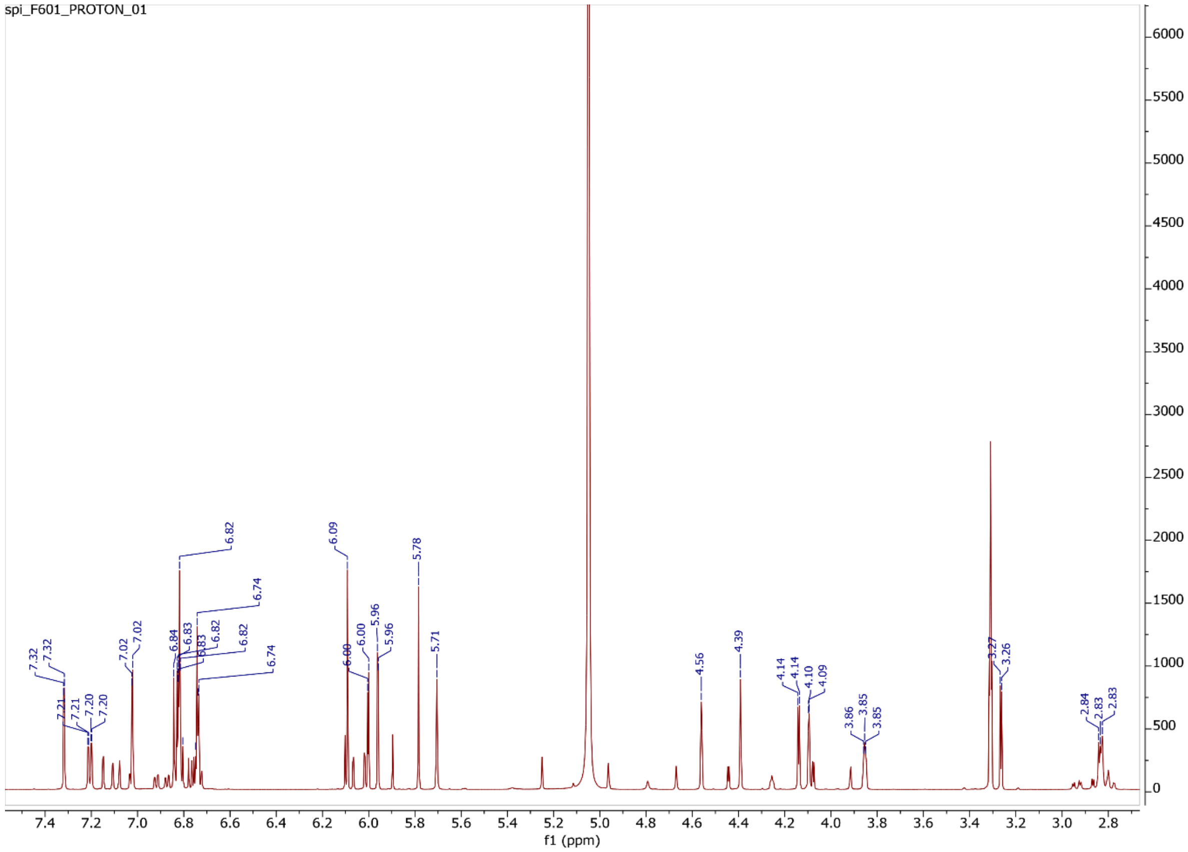

Figure S8: ^{1}H NMR spectrum of compound **2** (CD_3OD, 600 MHz, 280 K). Peak assignment was performed for major rotamers only.

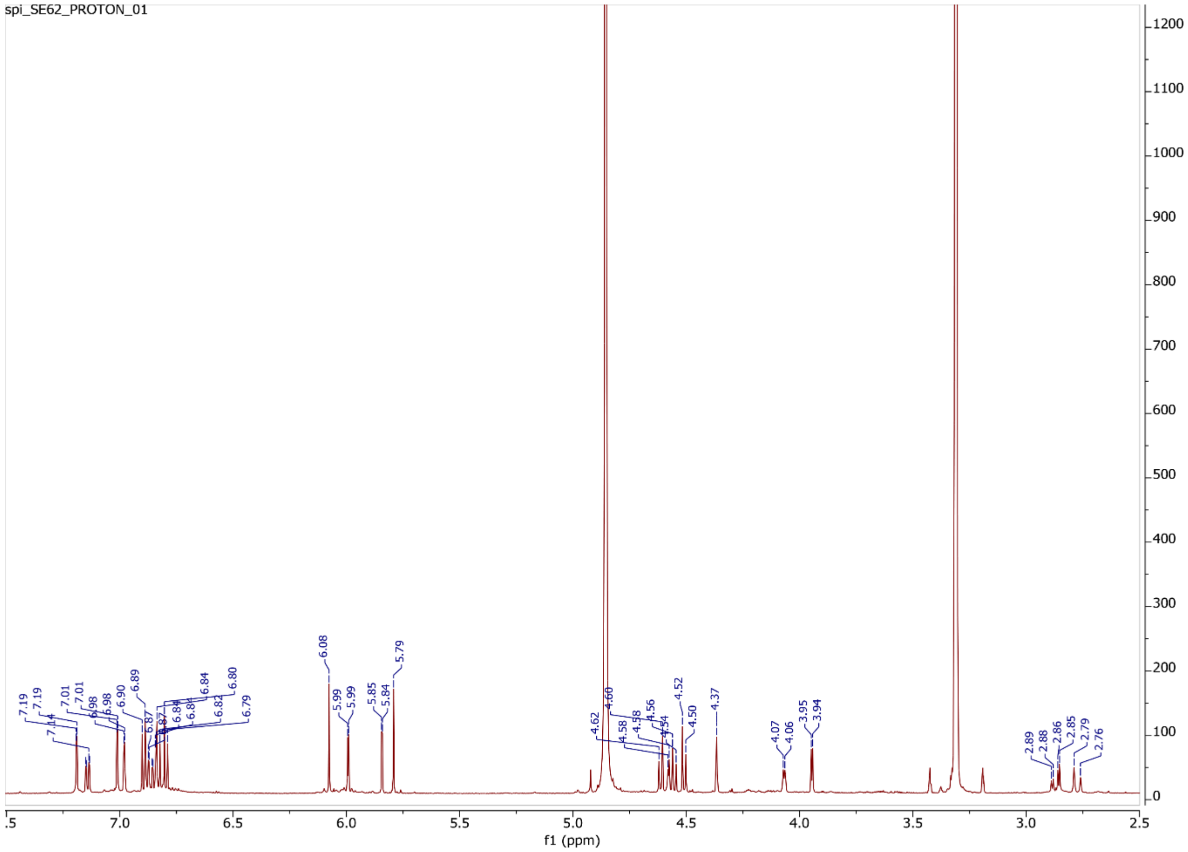

Figure S9: ^{1}H NMR spectrum of compound **3** (CD_3OD, 600 MHz, 280 K). Peak assignment was performed for major rotamers only.

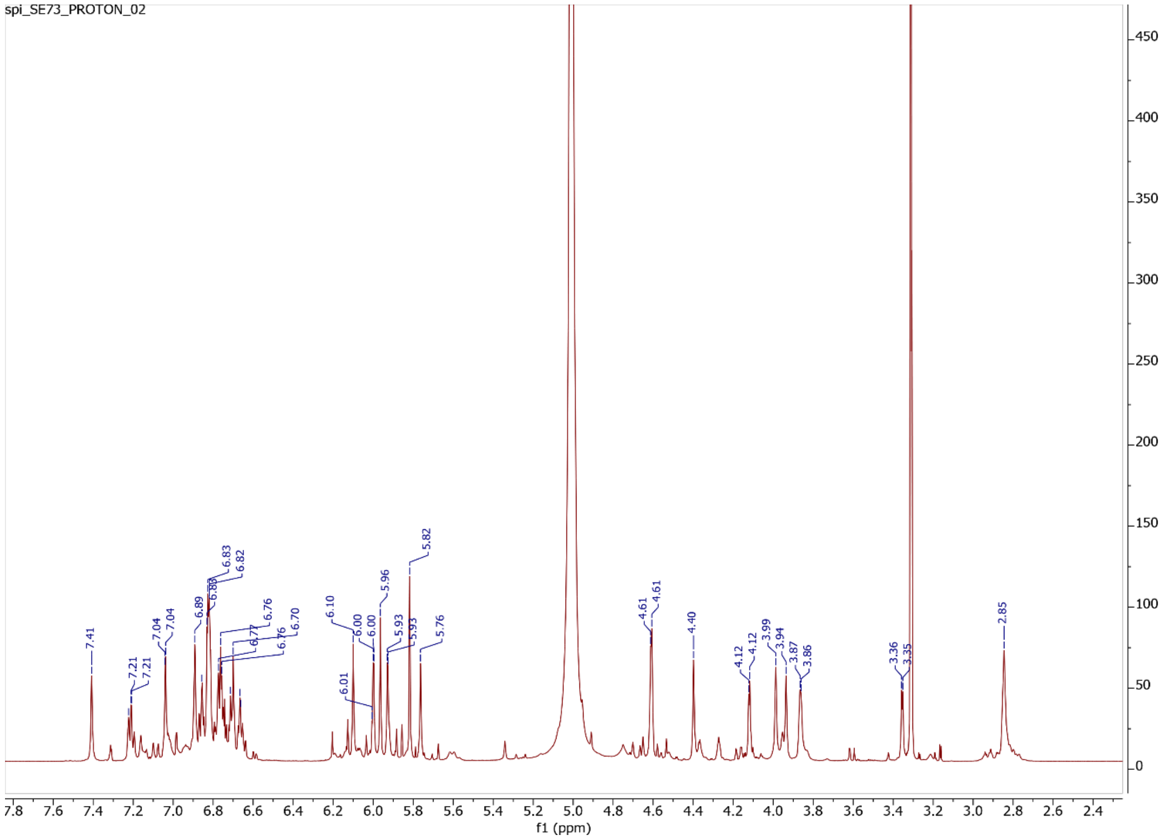

Figure S10: ^{1}H NMR spectrum of compound **3a** ($CDCl_3$, 600 MHz, 299 K). Peak assignment was performed for major rotamers only. 2.34 – 1.22 ppm: signals of acetate groups.

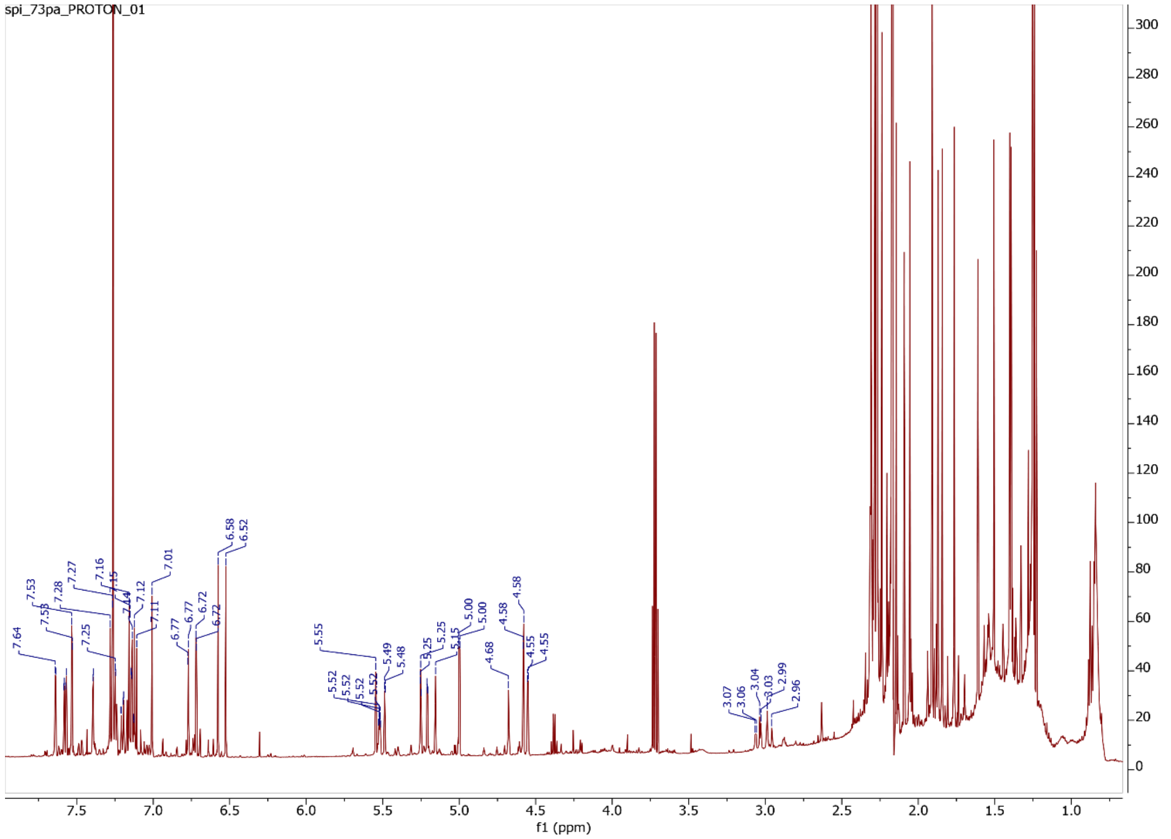

Figure S11: ^{1}H NMR spectrum of compound **4** (CD_3OD, 600 MHz, 280 K). Peak assignment was performed for major rotamers only.

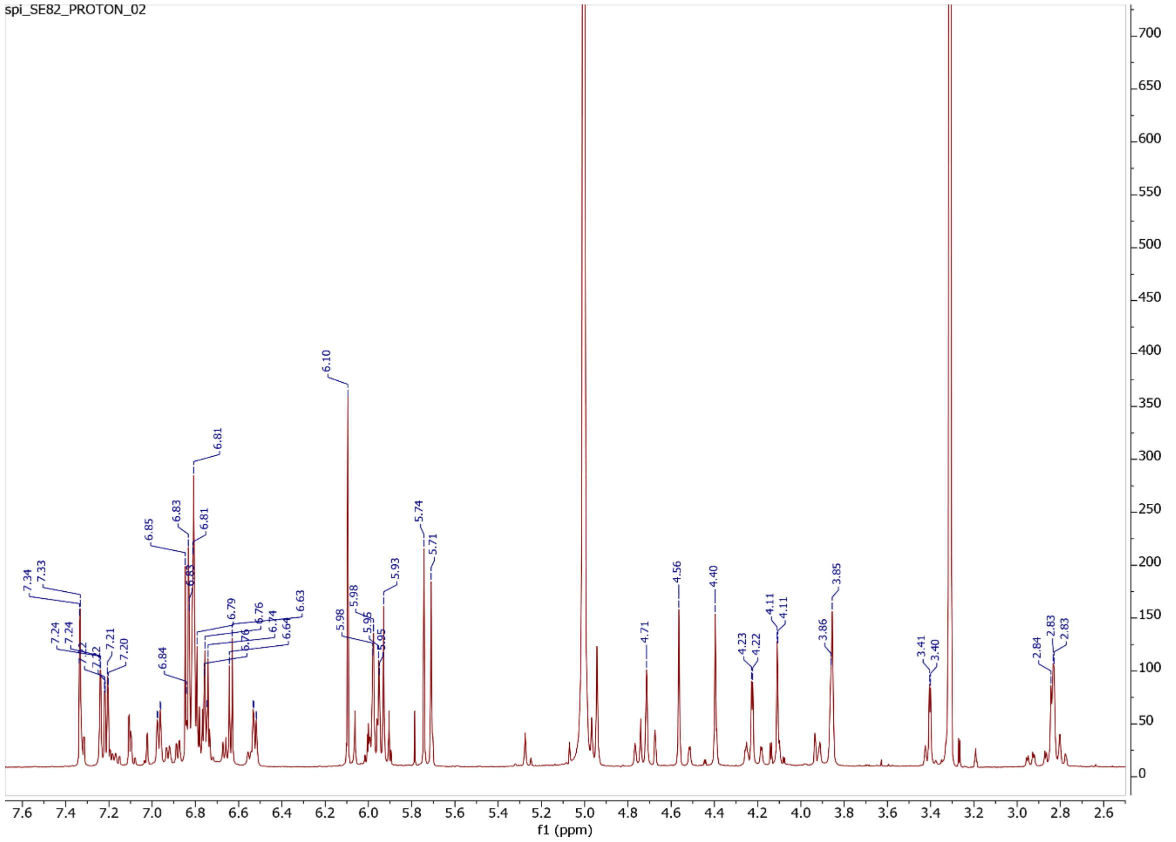

Figure S12: ^{1}H NMR spectrum of compound **5** (CD_3OD, 600 MHz, 280 K). Peak assignment was performed for major rotamers only.

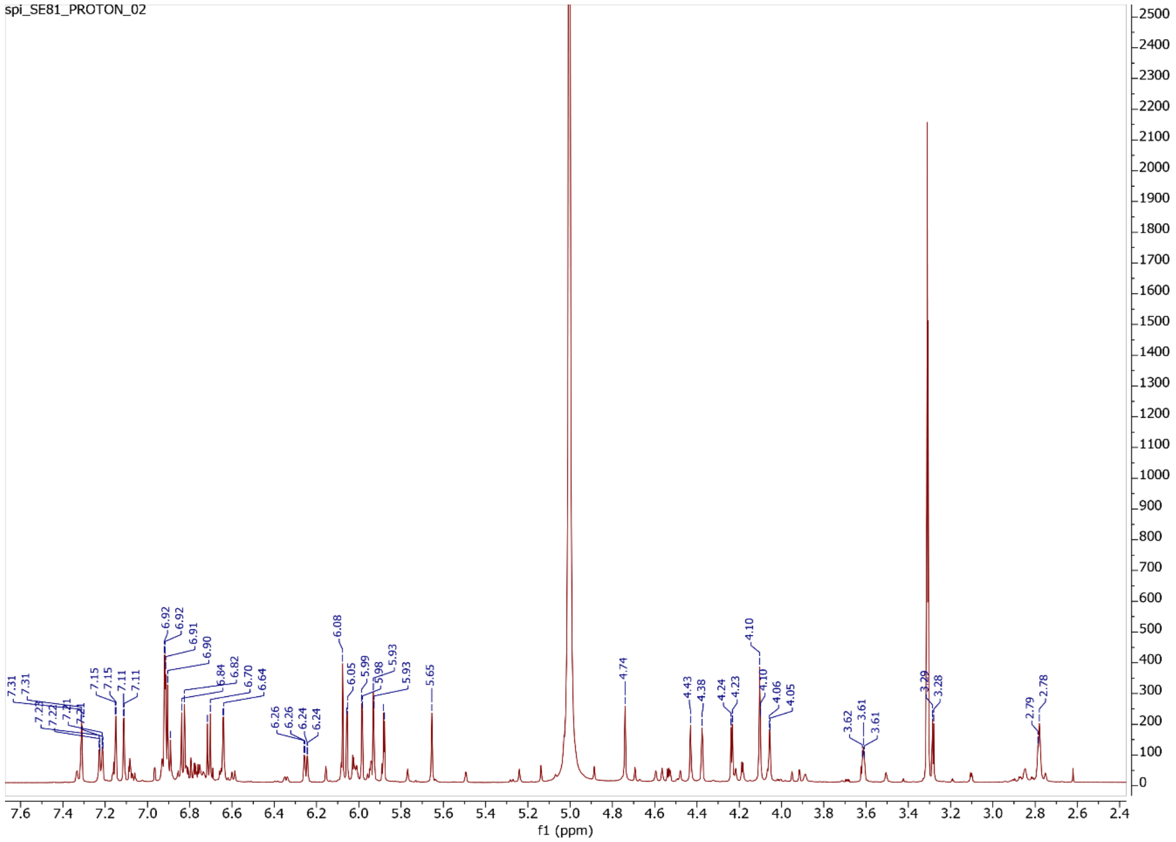

Figure S13: ^{1}H NMR spectrum of compound **6** (CD_3OD, 600 MHz, 280 K).

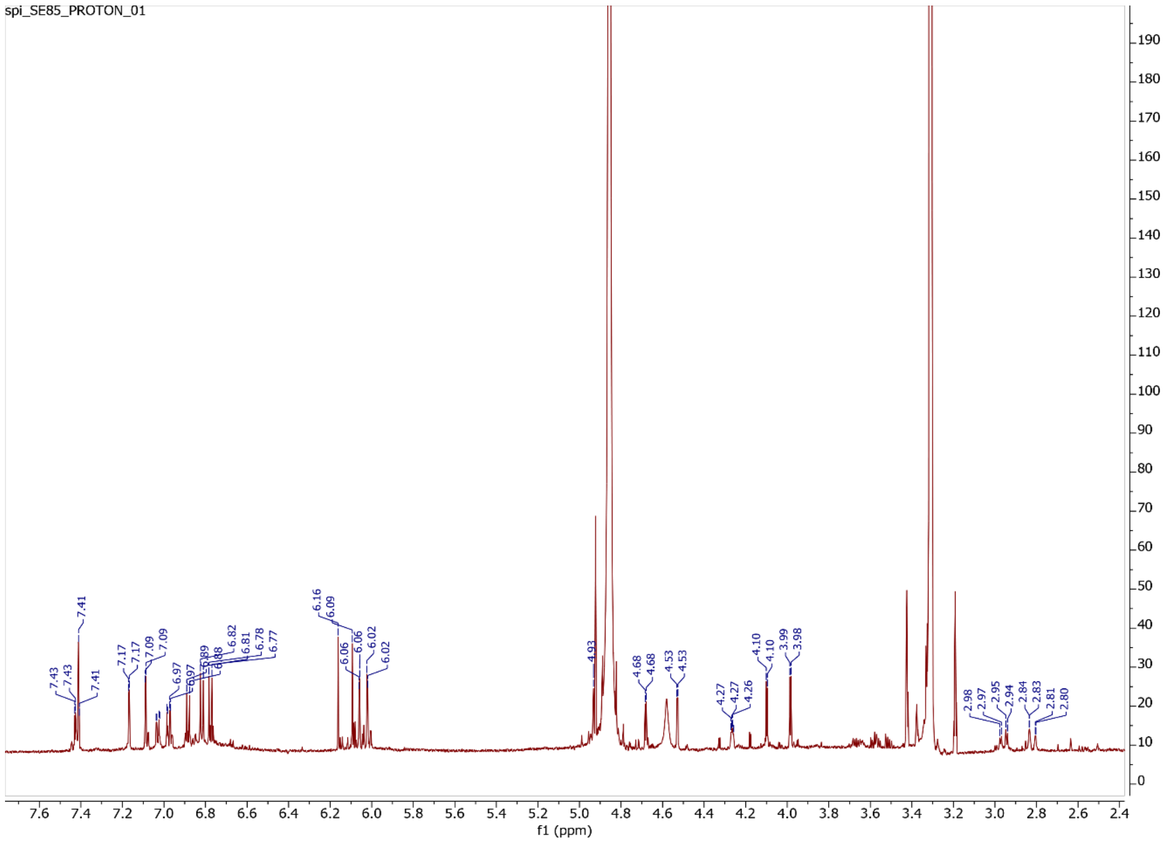

Figure S14: ^{1}H NMR spectrum of compound **7** (spectrum 2, top) compared to a spectrum of the reference compound (spectrum 1, bottom), ($CDCl_3$, 600 MHz).

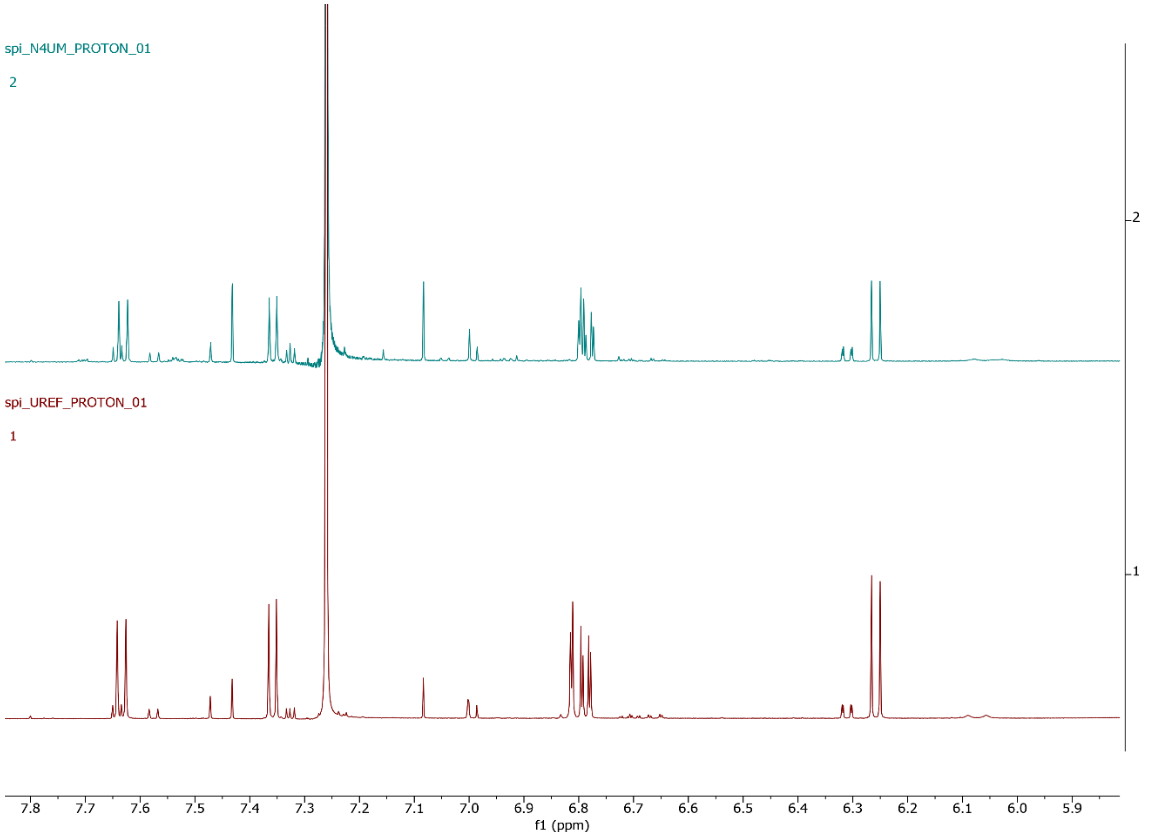

Figure S15: ^{1}H NMR spectrum of compound **8** ($CDCl_3$, 600 MHz).

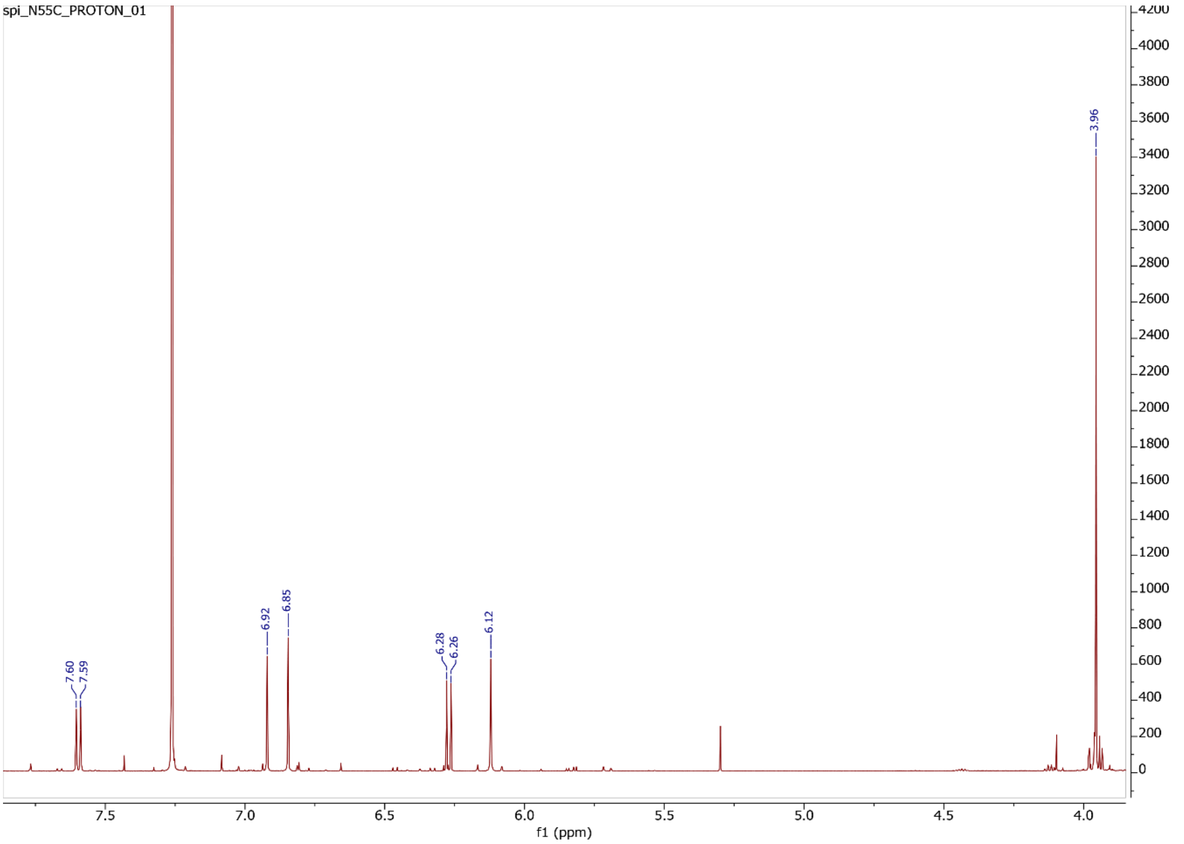

Figure S16: ^{1}H NMR spectrum of compound **9** (CD_3OD, 600 MHz).

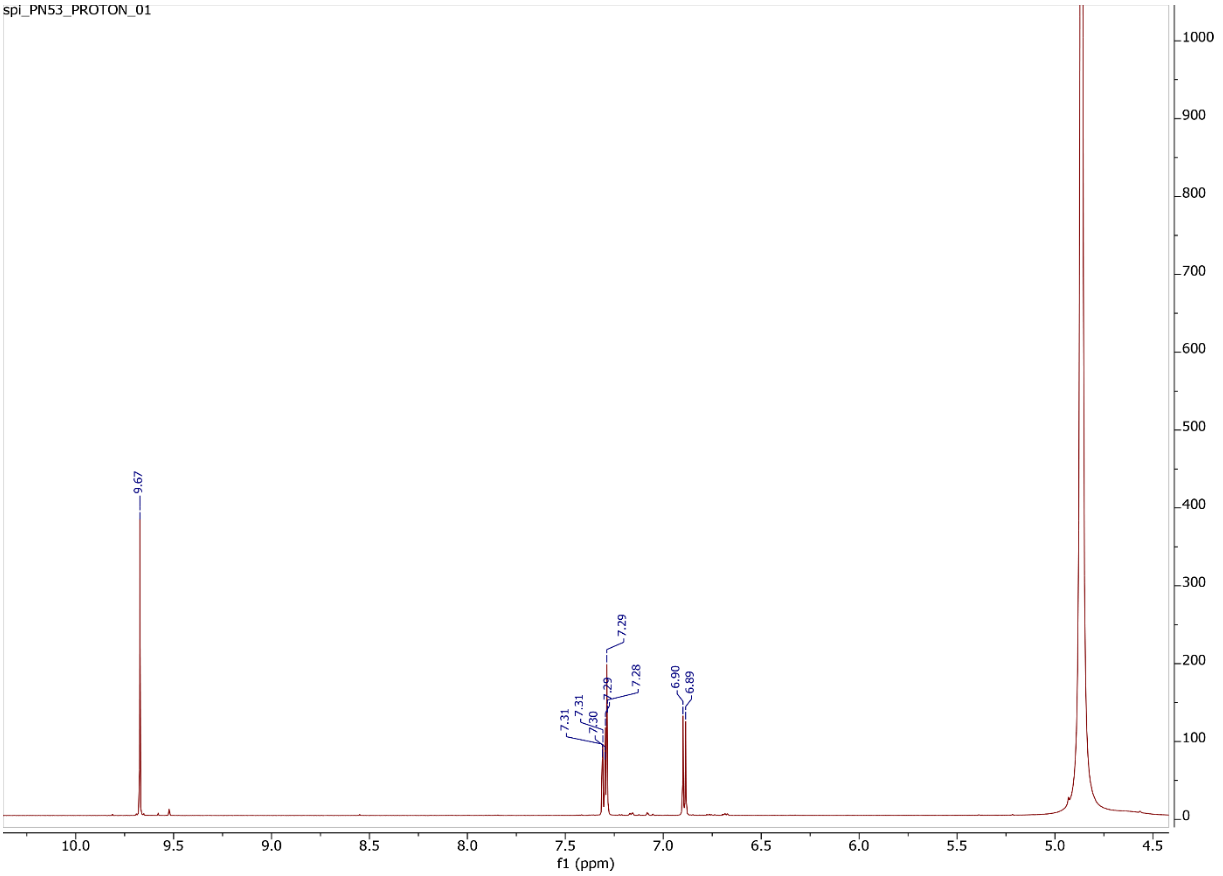

Figure S17: ^{1}H NMR spectrum of compound **10** (acetone-d_6, 600 MHz).

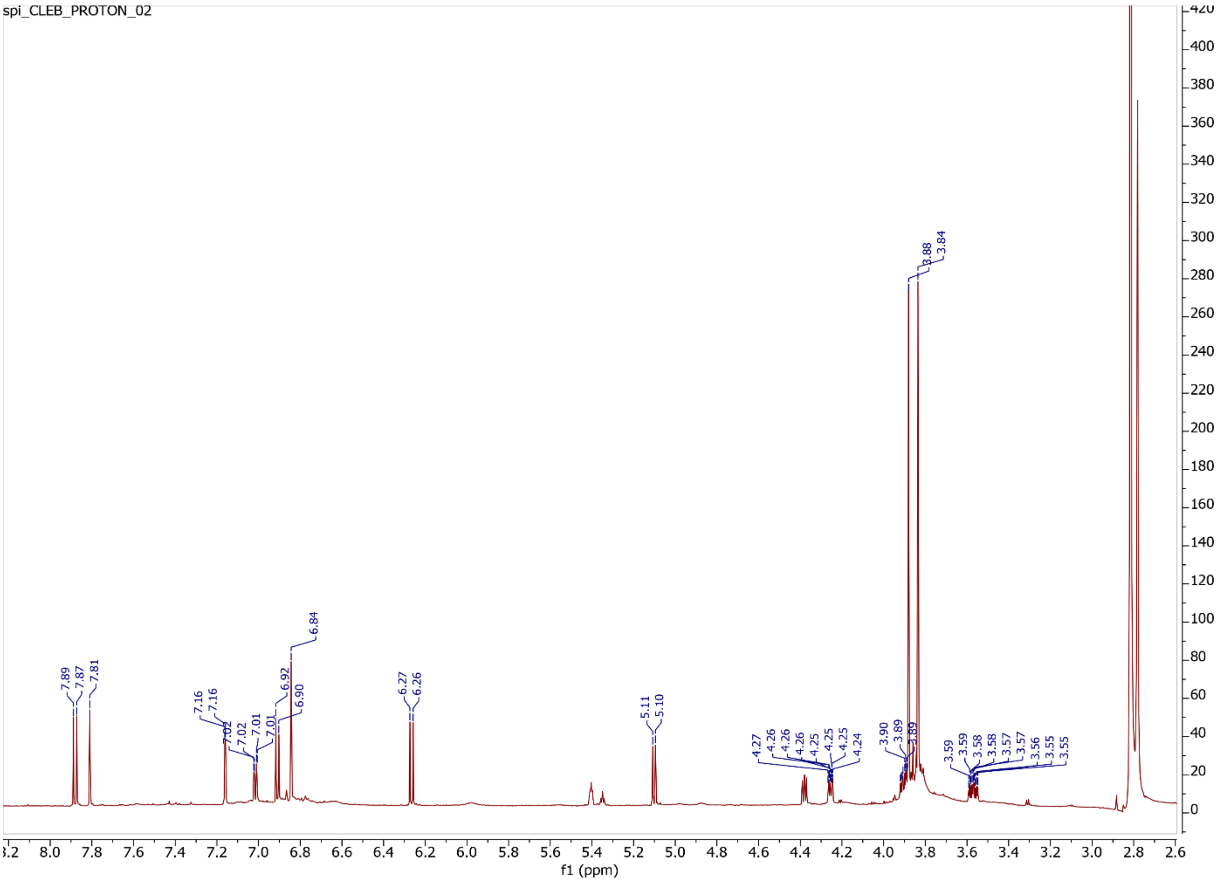

Figure S18: ^{1}H NMR spectrum of compound **11** (acetone-d_6, 600 MHz).

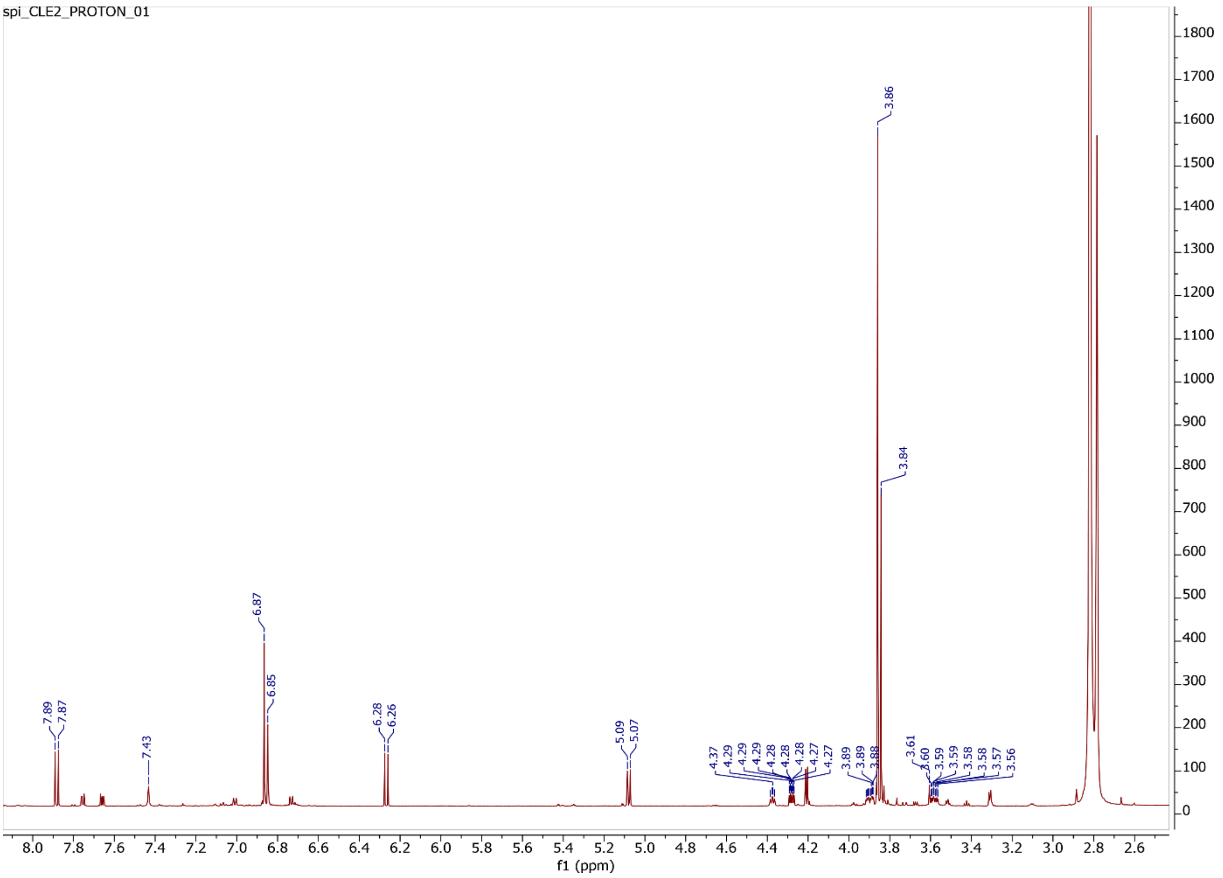

Figure S19: ^{1}H NMR spectrum of compound **12** (acetone-d_6, 600 MHz).

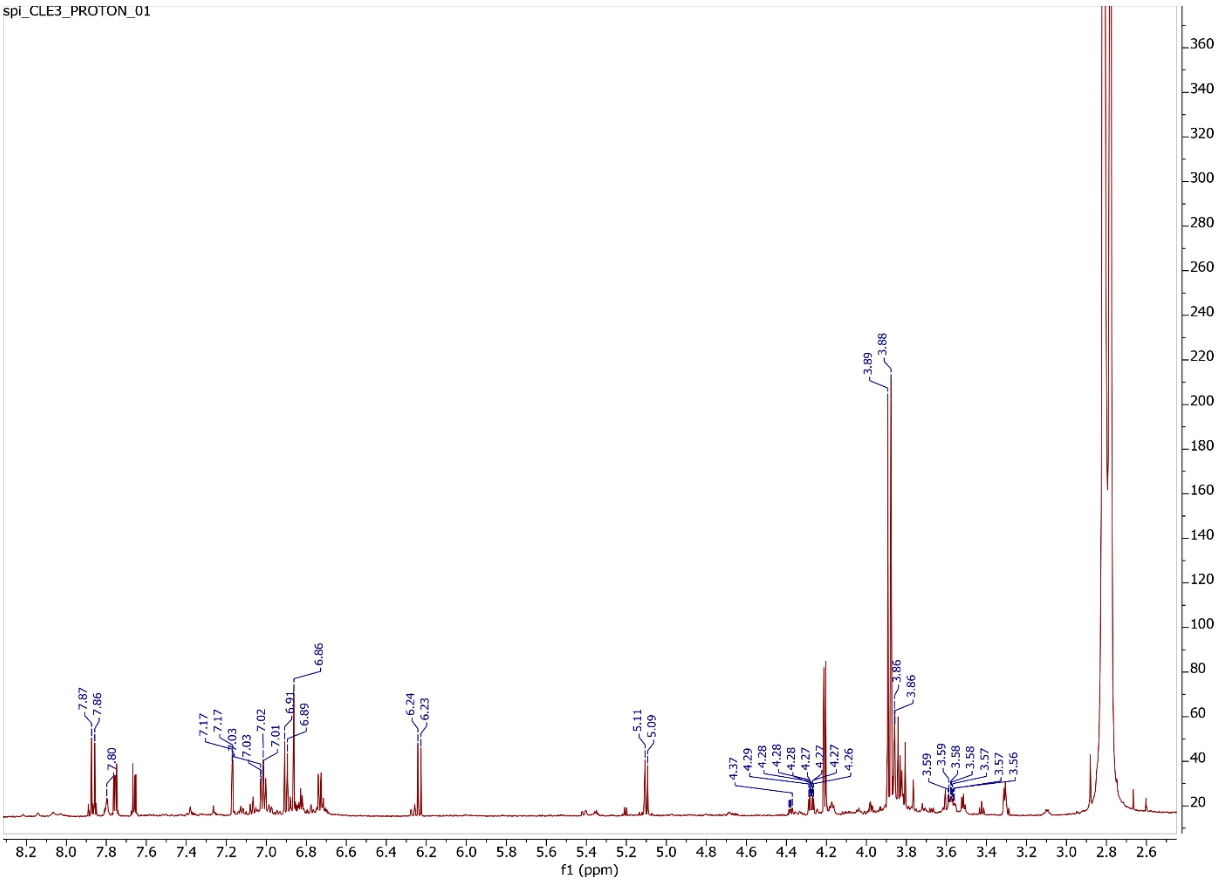

Figure S20: ^{1}H NMR spectrum of compound **13** (acetone-d_6, 600 MHz).

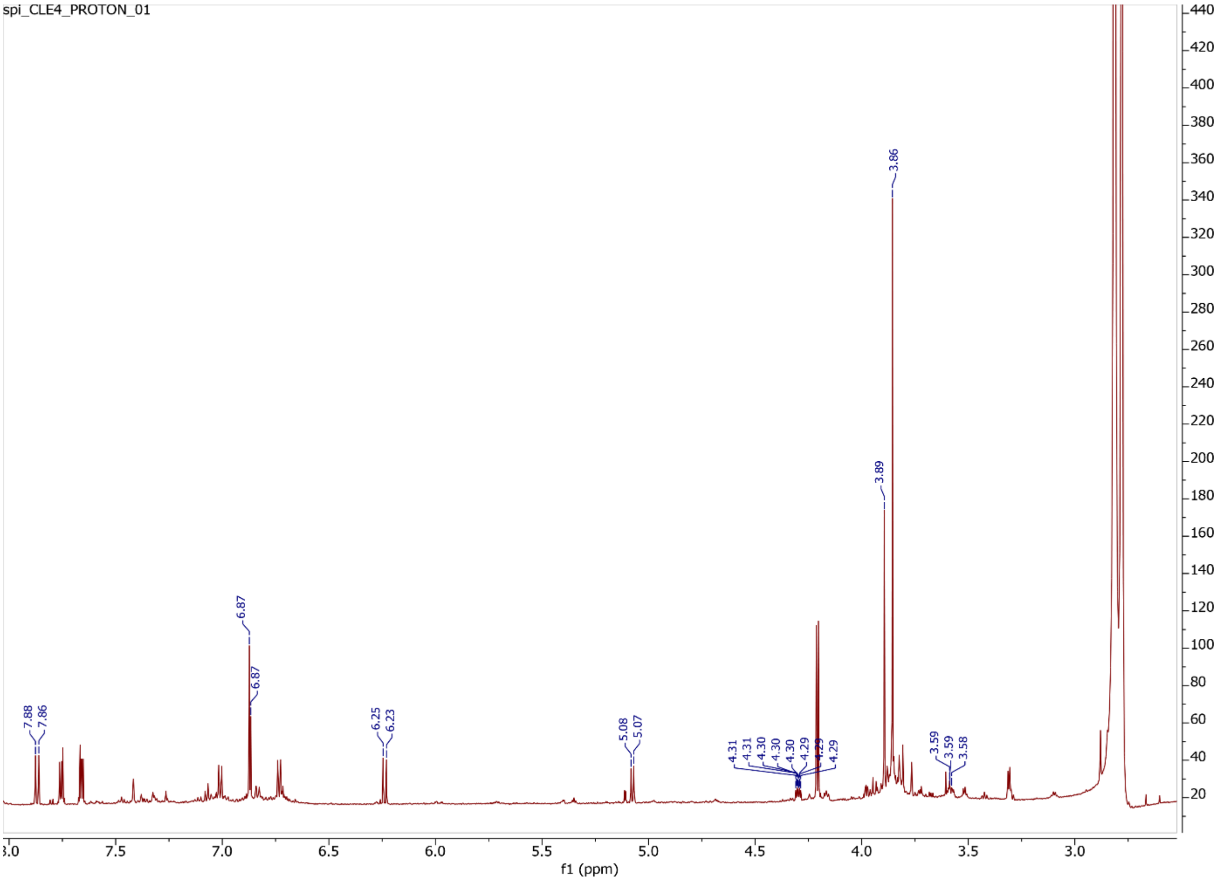

Figure S21: ^{1}H NMR spectrum of compound **14** (CD_3OD, 600 MHz).

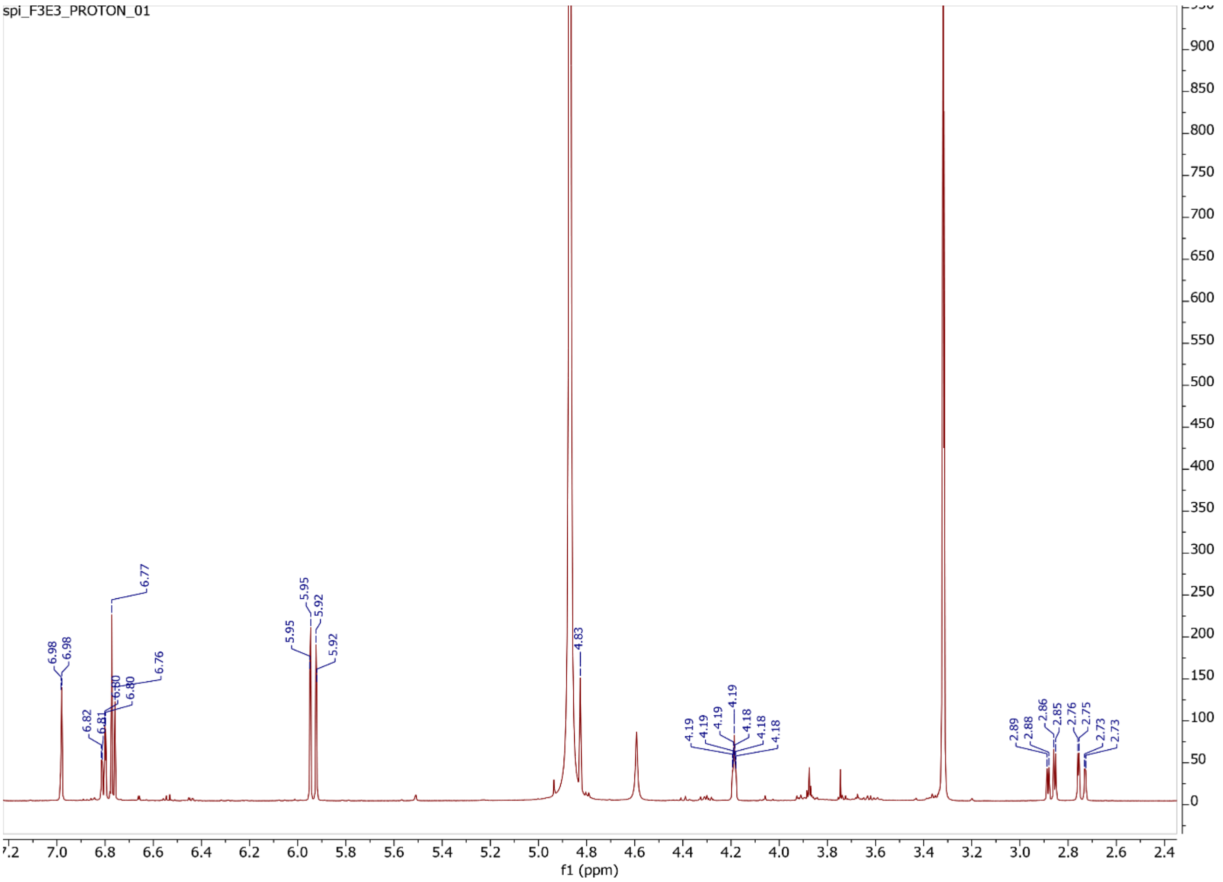

Figure S22: ^{1}H NMR spectrum of compound **16** (D_2O, 600 MHz).

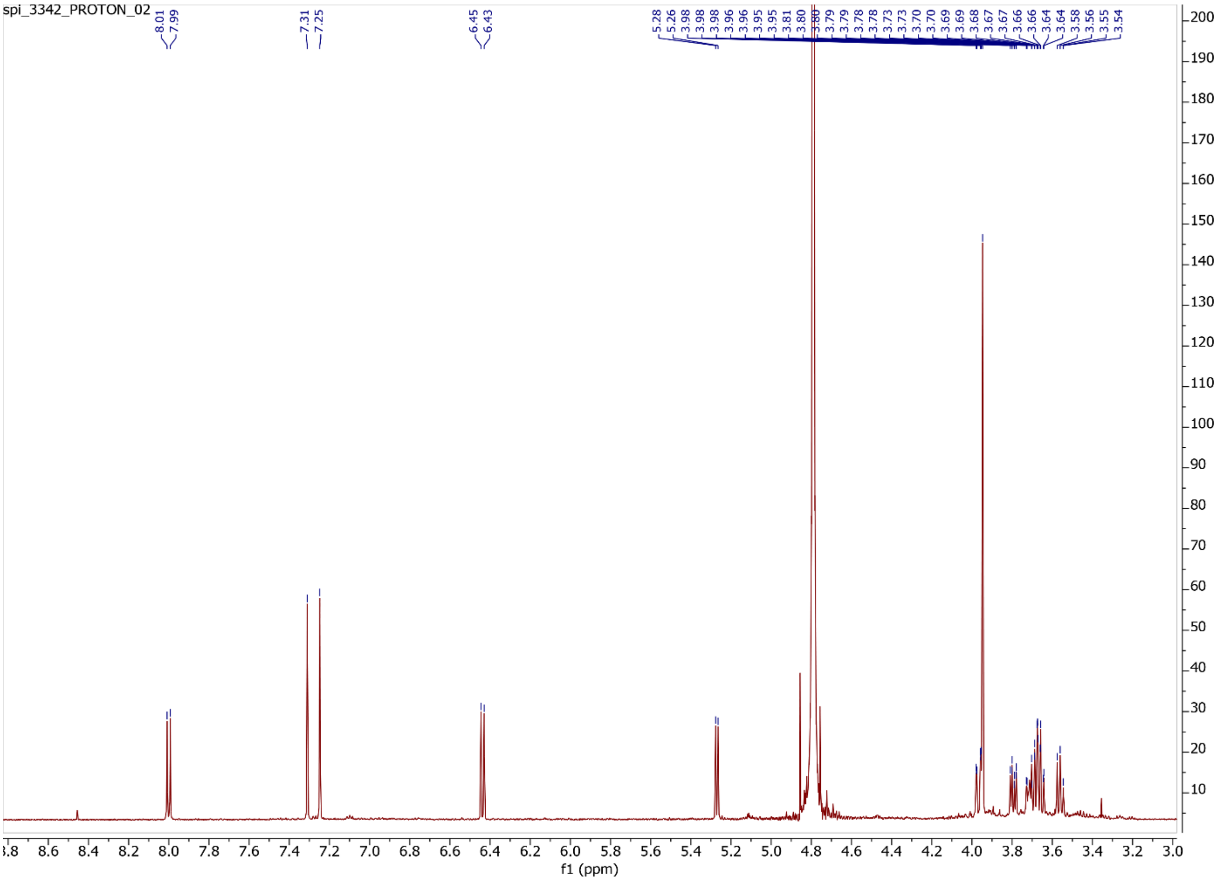

Supplement: Supplementary file 1 [file molecules-25-02287-s001.pdf]
